# Global, regional, and national prevalence of asthma in 2019: a systematic analysis and modelling study

**DOI:** 10.7189/jogh.12.04052

**Published:** 2022-06-29

**Authors:** Peige Song, Davies Adeloye, Hani Salim, Jhonathan PR Dos Santos, Harry Campbell, Aziz Sheikh, Igor Rudan

**Affiliations:** 1 School of Public Health and Women’s Hospital, Zhejiang University School of Medicine, Hangzhou, China; 2 Usher Institute, University of Edinburgh, Edinburgh, UK; 3 Department of Family Medicine, Faculty of Medicine and Health Sciences, University Putra Malaysia, Serdang, Malaysia; 4 Cornell University, Ithaca, New York, United States

## Abstract

**Background:**

Asthma has a significant impact on people of all ages, particularly children. A lack of universally accepted case definition and confirmatory tests and a poor understanding of major risks interfere with a global response. We aimed to provide global estimates of asthma prevalence and cases in 2019 across four main epidemiological case definitions – current wheezing, ever wheezing, current asthma, and ever asthma. We further investigated major associated factors to determine regional and national distributions of prevalence and cases for current wheezing and ever asthma.

**Methods:**

We identified relevant population-based studies published between January 1, 1990, and December 31, 2019. Using a multilevel multivariable mixed-effects meta-regression model, we assessed the age- and sex-adjusted associations of asthma with study-level variables, including year, setting, region and socio-demographic index (SDI). Using a random-effects meta-analysis, we then identified risk factors for current wheezing and asthma. From a “risk factor-based model”, which included current smoking, and biomass exposure for current wheezing, and rural setting, current smoking, biomass exposure, and SDI for ever asthma, we estimated case numbers and prevalence across regions and 201 countries and territories in 2019.

**Results:**

220 population-based studies conducted in 88 countries were retained. In 2019, the global prevalence estimates of asthma in people aged 5-69 years by various definitions, namely current wheezing, ever wheezing, current asthma, and ever asthma were 11.5% (95% confidence interval (CI) = 9.1-14.3), 17.9% (95% CI = 14.2-22.3), 5.4% (95% CI = 3.2-9.0) and 9.8% (95% CI = 7.8-12.2), respectively. These translated to 754.6 million (95% CI = 599. 7-943.4), 1181.3 million (95% CI = 938.0-1,471.0), 357.4 million (95% CI = 213.0-590.8), 645.2 million (95% CI = 513.1-806.2) cases, respectively. The overall prevalence of current wheezing among people aged 5-69 years was the highest in the African Region at 13.2% (95% CI = 10.5-16.5), and the lowest in the Americas Region at 10.0% (95% CI = 8.0-12.5). For ever asthma, the estimated prevalence in those aged 5-69 years was also the highest in the African Region at 11.3% (95% CI = 9.0-14.1), but the lowest in South-East Asia Region (8.8, 95% CI = 7.0-11.0).

**Conclusions:**

Although varying approaches to case identification in different settings make epidemiological estimates of asthma very difficult, this analysis reaffirms asthma as a common global respiratory condition before the COVID-19 pandemic in 2019, with higher prevalence than previously reported in many world settings.

Asthma is a major chronic respiratory disease substantially affecting the quality of life of people of all ages, particularly children who experience more years of poorer life quality compared to adults [1]. In 2019, Global Burden of Disease (GBD) collaborators estimated that over 260 million people globally had poorly controlled asthma (diagnosed asthma with wheezing within past 12 months) [1], with a high count of disabilities and premature deaths across many low- and middle-income countries (LMICs) [1,2].

To date, there is no universally accepted case definition or a combination of tests that are confirmatory for asthma. The varying approaches to defining asthma reflect a different understanding of the aetiology and inform epidemiological estimates across settings. Based on the responses to questions relating to self-reported diagnosis and respiratory symptoms over a set period, four main definitions have been reported, including “current wheezing”, “ever wheezing”, “newly-diagnosed asthma”, and “ever asthma”. According to reports from major international respiratory groups, including the International Study of Asthma and Allergies in Childhood (ISAAC), the Global Asthma Network (GAN), and the European Community Respiratory Health Survey (ECRHS), “current wheezing” (within the past 12 months) was found to be a sensitive epidemiological definition for active asthma, while the “ever-diagnosed asthma” definition was specific for lifetime asthma [3,4].

The challenges of combining prevalence data across asthma definitions, with inconsistencies in many settings, have been previously highlighted [4-6]. Guided by the above definitions, this study looks to improve the understanding of the global epidemiology of asthma, necessary to inform policy, research, and practice. First, we aimed to provide the global estimates of current wheezing, ever wheezing, current asthma, and ever asthma prevalence and cases in 2019, before the COVID-19 pandemic. We then investigated major associated factors of current wheezing and ever asthma across high-income countries (HICs) and LMICs to explore the regional and national distributions of asthma prevalence and cases in 2019.

## METHODS

This study was conducted in compliance with the Preferred Reporting Items for Systematic Reviews and Meta-Analyses (PRISMA) reporting guidelines [7].

### Search strategy

We combined various Medical Subject Headings (MeSH) terms on the epidemiology of asthma separately in four bibliographic databases (CINAHL, Embase, Global Health, and MEDLINE) to identify relevant population-based studies published between January 1, 1990, and December 31, 2019. Additional searches were conducted on Google Scholar. The reference lists of included articles and relevant systematic reviews on the epidemiology of asthma were further hand-searched. No language or geographical restrictions were applied. Full search strategies are provided in Table S1 in the **Online Supplementary Document**.

### Review and data extraction

Studies were included if they were conducted in the general population and reported the asthma prevalence or associated factors (see **Box 1** for eligibility criteria).

Box 1 **Eligibility criteria**

*Inclusion criteria*

1. Population-based studies that reported the prevalence and/or associated factors (adjusted odds ratio) of asthma in the general population;

2. Studies in 1 that defined asthma on the basis of wheezing symptoms or asthma diagnosis (ie, a self-reported history of wheeze symptoms in the preceding 12 months (current wheezing”) or asthma diagnosis by a physician (“ever asthma”));

3. Studies in 2 that employed a protocol or questionnaire that had been validated and/or implemented in large-scale epidemiology studies; and

4. Studies in 3 that were published between January 1, 1990 and December 31, 2019.

*Exclusion criteria*

1. Studies that were not population-based, that were derived from hospital or administrative data, or that were conducted in a specific population group which was not representative of the general population;

2. Studies that did not provide the prevalence and associated factors of asthma;

3. Studies where study design and case ascertainment of asthma were unclear; and

4. Reviews, case reports, opinion-based articles, and viewpoints.

Title and abstract screening, followed by a full-text review and data extraction were conducted independently by DA, HS, and PS. Discrepancies were resolved by consensus. We extracted data on first author, year of publication, study location, country, study design, sampling method, study setting, year of investigation, asthma case definition, protocol employed, sample size, proportion of female participants, average (or median) age of participants, number of asthma cases, and prevalence. The year-specific socio-demographic index (SDI) for each country was also extracted [8]. The geographic region was categorized according to the World Health Organization (WHO) and included the African Region (AFR), the Region of the Americas (AMR), the South-East Asia Region (SEAR), the European Region (EUR), the Eastern Mediterranean Region (EMR) and the Western Pacific Region (WPR). The socio-economic region was categorized by the World Bank (WB) as HICs and LMICs. More details are provided in Table S2 in the **Online Supplementary Document**.

When provided, stratified prevalence data were extracted by age group, sex, or setting. For studies with censored age group band, we assumed the same interval as reported for the other age groups within the same study. Associated factors (and definitions) and the corresponding fully-adjusted odds ratios (ORs) were extracted from articles that estimated the risk of asthma using multivariable logistic regressions.

### Statistical analysis

**Figure 1:**
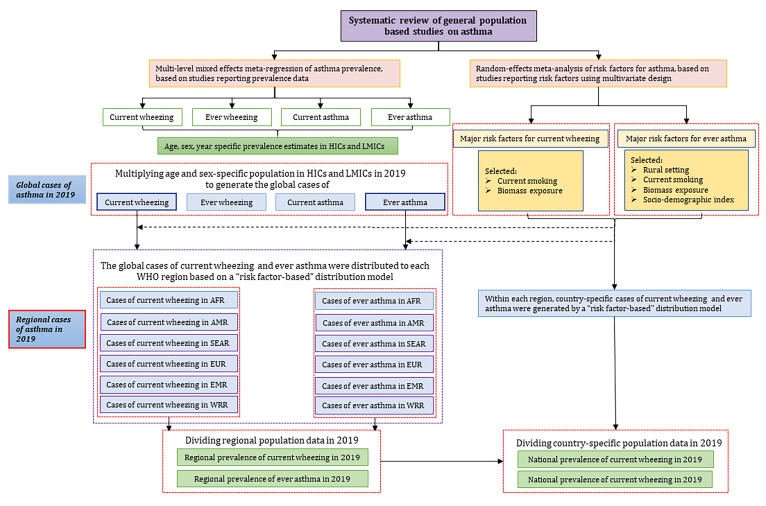
Step-wise approach to modelling the global prevalence of asthma. HICs – high-income countries, LMICs – low- and middle-income countries, WHO – World Health Organization, AFR – African Region, AMR – Region of the Americas, SEAR – South-East Asia Region, EUR – European Region, EMR – Eastern Mediterranean Region, WPR – Western Pacific Region.

To reduce heterogeneity, we sorted data and estimates by: 1) current wheezing: wheezing in the past 12 months; and 2) ever wheezing: ever wheezing across the lifespan; 3) current asthma: newly-diagnosed asthma in the past 12 months; 4) ever asthma: ever diagnosed asthma across lifespan. The detailed analytic approach is described in **Figure 1** as well as Appendix 2 in the **Online Supplementary Document**.

### Modelling the global prevalence of asthma

To accommodate for the hierarchical data structure, a multilevel multivariable mixed-effects meta-regression model was fitted to account for the uncertainty of reported prevalence estimates and their clustering and relations to various factors. A restricted cubic spline basis for age was used to model the prevalence of asthma as a function of age. Due to limited data sources at young (<5 years) and older ages (≥70 years), we restricted estimates to 5-69 years. Modelling was separately conducted for HICs and LMICs and details are described in Table S4 in the **Online Supplementary Document**.

### Estimation of the global numbers of people with asthma in 2019

Based on the estimated age- and sex-specific prevalence of current wheezing, ever wheezing, current asthma and ever asthma in HICs and LMICs in 2019, we generated the numbers of people affected by current wheezing, ever wheezing, current asthma, and ever asthma respectively, by multiplying the age- and sex-specific de-facto population in 2019, as reported by the United Nations Population Division (UNPD) [9].

### Estimation of the regional and national numbers of people with asthma in 2019

To account for economics and geography simultaneously, we purposely classified the world into ten WB-WHO regions. We estimated the regional cases of current wheezing and ever asthma using a “risk factor-based model”. Associated factors assessed in at least three individual studies were included for a random-effects (DerSimonian and Laird method) meta-analysis [10], based on which two major associated factors, namely current smoking and biomass exposure, were included in the “risk factor-based model” for current wheezing. Four associated factors, including rural setting, current smoking and biomass exposure and SDI were selected for ever asthma.

Using the same “risk factor-based model” approach when distributing the global cases of current wheezing and ever asthma, we estimated the number of affected people for 201 countries and territories in 2019.

All analyses were conducted with STATA version 14.0 (STATA Corporation, College Station, TX, USA) and R version 3.3.0 (R Foundation for Statistical Computing, Vienna, Austria) using the “metafor” (version 1.9-7) package.

## RESULTS

**Figure 2:**
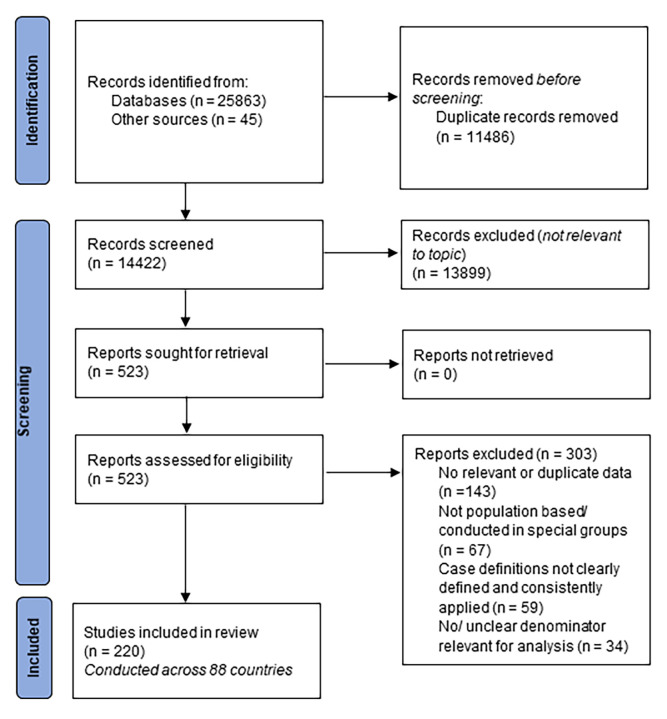
PRISMA flow diagram of selection of studies.

From 14 422 eligible records after removing duplicates, 523 full texts were assessed. Following the application of the eligibility criteria, 220 population-based articles across 88 countries reporting the prevalence and/or associated factors of asthma were retained. Details of study selection process are presented in **Figure 2** and the contribution of data sources across world regions is provided in Figure S1 in the **Online Supplementary Document**. The prevalence of current wheezing, ever wheezing, current asthma and ever asthma, by age and sex group, based on contributing data points from included articles are shown in Figure S2 in the **Online Supplementary Document****.** Characteristics and references of all included studies are listed in Tables S5-S6 in the **Online Supplementary Document**.

The estimated prevalence of current wheezing, ever wheezing, current asthma, and ever asthma by age in HICs and LMICs among people aged 5-69 years in 2019 is shown in **Table 1** and Figure S3 in the **Online Supplementary Document**. The prevalence was consistently higher in HICs than in LMICs across the whole age span of 5-69 years, and the disparity was pronounced for current wheezing and ever asthma- the most sensitive and most specific definitions of asthma.

**Table 1:** Estimated prevalence of current wheezing, ever wheezing, current asthma, and ever asthma in HICs, LMICs and worldwide in 2019, by age group and sex

Age group	Male			Female			Overall		
**HIC**	**LMIC**	**Worldwide**	**HIC**	**LMIC**	**Worldwide**	**HIC**	**LMIC**	**Worldwide**
**Current wheezing (%, 95% CI)**									
5-9 y	13.17	12.17	12.28	12.04	11.12	11.22	12.62	11.66	11.77
	(10.72-16.07)	(9.75-15.10)	(9.85-15.20)	(9.78-14.74)	(8.88-13.83)	(8.98-13.93)	(10.26-15.42)	(9.33-14.48)	(9.43-14.58)
10-14 y	13.89	12.85	12.96	12.71	11.74	11.85	13.31	12.31	12.43
	(11.32-16.93)	(10.30-15.91)	(10.41-16.03)	(10.33-15.54)	(9.39-14.59)	(9.50-14.70)	(10.84-16.25)	(9.86-15.27)	(9.97-15.38)
15-19 y	13.36	12.35	12.47	12.22	11.28	11.39	12.80	11.83	11.95
	(10.88-16.31)	(9.89-15.32)	(10.00-15.43)	(9.92-14.96)	(9.01-14.04)	(9.12-14.14)	(10.41-15.65)	(9.47-14.70)	(9.58-14.81)
20-24 y	12.93	11.95	12.07	11.82	10.91	11.03	12.39	11.44	11.57
	(10.46-15.88)	(9.51-14.91)	(9.63-15.03)	(9.54-14.56)	(8.66-13.66)	(8.77-13.77)	(10.01-15.24)	(9.10-14.30)	(9.21-14.42)
25-29 y	12.67	11.70	11.84	11.58	10.69	10.81	12.15	11.21	11.34
	(10.19-15.64)	(9.27-14.68)	(9.40-14.82)	(9.29-14.33)	(8.44-13.44)	(8.56-13.56)	(9.76-15.02)	(8.87-14.07)	(8.99-14.21)
30-34 y	12.53	11.58	11.71	11.45	10.57	10.69	12.01	11.08	11.21
	(10.06-15.50)	(9.15-14.54)	(9.28-14.68)	(9.17-14.20)	(8.33-13.31)	(8.45-13.44)	(9.64-14.88)	(8.75-13.94)	(8.88-14.07)
35-39 y	12.47	11.52	11.67	11.39	10.51	10.65	11.94	11.02	11.17
	(10.01-15.42)	(9.10-14.47)	(9.25-14.62)	(9.13-14.13)	(8.29-13.25)	(8.42-13.39)	(9.58-14.79)	(8.70-13.86)	(8.84-14.01)
40-44 y	12.43	11.48	11.65	11.36	10.48	10.63	11.91	10.99	11.15
	(9.99-15.37)	(9.08-14.42)	(9.24-14.59)	(9.10-14.09)	(8.27-13.21)	(8.41-13.36)	(9.56-14.74)	(8.68-13.82)	(8.83-13.98)
45-49 y	12.40	11.45	11.63	11.33	10.46	10.61	11.88	10.96	11.12
	(9.96-15.34)	(9.05-14.39)	(9.22-14.57)	(9.08-14.06)	(8.24-13.18)	(8.39-13.34)	(9.53-14.71)	(8.65-13.79)	(8.81-13.96)
50-54 y	12.37	11.42	11.61	11.30	10.43	10.59	11.84	10.92	11.10
	(9.92-15.32)	(9.01-14.38)	(9.19-14.56)	(9.04-14.04)	(8.21-13.16)	(8.37-13.33)	(9.48-14.69)	(8.61-13.77)	(8.78-13.94)
55-59 y	12.34	11.39	11.60	11.27	10.40	10.59	11.80	10.89	11.09
	(9.87-15.31)	(8.97-14.37)	(9.17-14.57)	(9.00-14.03)	(8.17-13.15)	(8.35-13.34)	(9.43-14.67)	(8.57-13.76)	(8.75-13.95)
60-64 y	12.31	11.36	11.59	11.24	10.37	10.58	11.76	10.86	11.07
	(9.82-15.32)	(8.92-14.37)	(9.13-14.60)	(8.94-14.04)	(8.12-13.16)	(8.32-13.37)	(9.37-14.67)	(8.51-13.75)	(8.72-13.97)
65-69 y	12.27	11.33	11.57	11.21	10.35	10.56	11.72	10.82	11.04
	(9.75-15.34)	(8.86-14.39)	(9.09-14.62)	(8.89-14.05)	(8.07-13.17)	(8.27-13.39)	(9.30-14.67)	(8.45-13.75)	(8.66-13.98)
Overall (5-69 y)	12.68	11.84	11.97	11.59	10.80	10.92	12.14	11.32	11.45
	(10.21-15.65)	(9.40-14.81)	(9.52-14.94)	(9.30-14.34)	(8.55-13.55)	(8.66-13.67)	(9.76-15.01)	(8.98-14.19)	(9.10-14.32)
**Ever wheezing (%, 95% CI)**									
5-9 y	26.51	24.19	24.44	24.11	21.95	22.18	25.34	23.11	23.35
	(21.45-32.26)	(19.65-29.40)	(19.84-29.71)	(19.40-29.56)	(17.73-26.84)	(17.91-27.14)	(20.45-30.94)	(18.72-28.17)	(18.91-28.46)
10-14 y	28.73	26.29	26.56	26.21	23.91	24.17	27.50	25.14	25.41
	(23.39-34.74)	(21.46-31.77)	(21.68-32.10)	(21.20-31.92)	(19.40-29.09)	(19.61-29.41)	(22.32-33.37)	(20.47-30.48)	(20.68-30.8)
15-19 y	22.48	20.42	20.66	20.35	18.44	18.66	21.44	19.46	19.69
	(17.99-27.71)	(16.41-25.11)	(16.59-25.41)	(16.19-25.25)	(14.75-22.80)	(14.92-23.09)	(17.11-26.51)	(15.61-24.00)	(15.79-24.29)
20-24 y	18.71	16.92	17.15	16.86	15.21	15.42	17.81	16.09	16.31
	(14.76-23.43)	(13.42-21.11)	(13.59-21.40)	(13.24-21.23)	(12.02-19.07)	(12.17-19.35)	(14.02-22.36)	(12.74-20.12)	(12.91-20.41)
25-29 y	16.95	15.30	15.53	15.24	13.73	13.94	16.13	14.53	14.76
	(13.26-21.42)	(12.04-19.25)	(12.21-19.56)	(11.87-19.36)	(10.76-17.35)	(10.91-17.63)	(12.59-20.44)	(11.42-18.33)	(11.58-18.62)
30-34 y	16.44	14.83	15.06	14.77	13.30	13.51	15.64	14.08	14.30
	(12.82-20.84)	(11.63-18.71)	(11.81-19.03)	(11.47-18.83)	(10.40-16.86)	(10.55-17.14)	(12.17-19.88)	(11.03-17.8)	(11.19-18.10)
35-39 y	16.71	15.08	15.34	15.02	13.53	13.76	15.89	14.31	14.57
	(13.04-21.17)	(11.84-19.01)	(12.03-19.37)	(11.67-19.13)	(10.58-17.14)	(10.75-17.45)	(12.38-20.18)	(11.22-18.09)	(11.40-18.42)
40-44 y	17.40	15.71	16.01	15.65	14.11	14.37	16.55	14.92	15.20
	(13.61-21.98)	(12.36-19.77)	(12.58-20.16)	(12.19-19.89)	(11.05-17.83)	(11.25-18.19)	(12.91-20.96)	(11.71-18.81)	(11.92-19.19)
45-49 y	18.19	16.44	16.76	16.38	14.78	15.06	17.30	15.61	15.92
	(14.26-22.93)	(12.96-20.65)	(13.20-21.06)	(12.78-20.77)	(11.59-18.65)	(11.81-19.03)	(13.53-21.87)	(12.28-19.65)	(12.51-20.05)
50-54 y	19.01	17.20	17.55	17.14	15.47	15.79	18.09	16.33	16.67
	(14.92-23.92)	(13.57-21.57)	(13.83-22.02)	(13.38-21.69)	(12.15-19.50)	(12.38-19.92)	(14.16-22.82)	(12.85-20.53)	(13.10-20.97)
55-59 y	19.86	17.99	18.39	17.92	16.19	16.56	18.89	17.08	17.47
	(15.59-24.96)	(14.19-22.54)	(14.49-23.06)	(14.00-22.66)	(12.71-20.40)	(12.99-20.89)	(14.79-23.81)	(13.44-21.46)	(13.73-21.97)
60-64 y	20.74	18.8	19.26	18.73	16.94	17.37	19.71	17.85	18.29
	(16.28-26.04)	(14.82-23.56)	(15.17-24.15)	(14.62-23.68)	(13.29-21.35)	(13.60-21.90)	(15.43-24.84)	(14.03-22.43)	(14.37-23.00)
65-69 y	21.64	19.64	20.14	19.57	17.72	18.18	20.56	18.64	19.12
	(16.97-27.18)	(15.46-24.62)	(15.84-25.26)	(15.26-24.74)	(13.88-22.35)	(14.22-22.95)	(16.08-25.91)	(14.63-23.44)	(15.00-24.06)
Overall (5-69 y)	19.99	18.64	18.85	18.09	16.76	16.97	19.06	17.72	17.93
	(15.80-24.97)	(14.86-23.13)	(15.01-23.41)	(14.22-22.73)	(13.30-20.92)	(13.44-21.20)	(15.02-23.87)	(14.09-22.04)	(14.24-22.32)
**Current asthma (%, 95% CI)**									
5-9 y	15.24	13.58	13.76	14.46	12.87	13.04	14.86	13.24	13.41
	(11.34-20.19)	(8.00-22.12)	(8.36-21.91)	(10.73-19.20)	(7.56-21.07)	(7.90-20.86)	(11.04-19.70)	(7.79-21.61)	(8.14-21.41)
10-14 y	12.49	11.09	11.24	11.82	10.49	10.64	12.16	10.80	10.95
	(9.25-16.65)	(6.46-18.38)	(6.77-18.19)	(8.74-15.80)	(6.09-17.46)	(6.40-17.27)	(9.00-16.23)	(6.28-17.94)	(6.59-17.75)
15-19 y	6.09	5.36	5.45	5.74	5.06	5.14	5.92	5.21	5.30
	(4.44-8.30)	(3.06-9.22)	(3.22-9.12)	(4.18-7.84)	(2.88-8.71)	(3.04-8.61)	(4.31-8.08)	(2.98-8.98)	(3.13-8.87)
20-24 y	4.33	3.81	3.87	4.08	3.59	3.65	4.21	3.70	3.77
	(3.10-6.03)	(2.15-6.66)	(2.27-6.58)	(2.92-5.68)	(2.02-6.28)	(2.14-6.21)	(3.01-5.86)	(2.09-6.48)	(2.20-6.40)
25-29 y	4.01	3.52	3.59	3.78	3.32	3.38	3.90	3.42	3.49
	(2.85-5.62)	(1.98-6.20)	(2.10-6.11)	(2.68-5.30)	(1.86-5.84)	(1.98-5.77)	(2.77-5.47)	(1.92-6.02)	(2.04-5.95)
30-34 y	4.18	3.67	3.74	3.93	3.46	3.52	4.06	3.56	3.63
	(2.96-5.86)	(2.06-6.45)	(2.19-6.36)	(2.79-5.52)	(1.94-6.08)	(2.06-6.00)	(2.88-5.70)	(2.00-6.27)	(2.13-6.19)
35-39 y	4.31	3.79	3.87	4.06	3.57	3.65	4.19	3.68	3.76
	(3.05-6.05)	(2.13-6.66)	(2.28-6.56)	(2.87-5.71)	(2.00-6.28)	(2.14-6.19)	(2.97-5.89)	(2.06-6.48)	(2.21-6.38)
40-44 y	4.32	3.80	3.89	4.07	3.58	3.66	4.20	3.69	3.78
	(3.06-6.08)	(2.13-6.69)	(2.30-6.58)	(2.88-5.73)	(2.01-6.31)	(2.16-6.21)	(2.97-5.91)	(2.07-6.50)	(2.23-6.40)
45-49 y	4.23	3.72	3.81	3.99	3.50	3.59	4.11	3.61	3.70
	(2.99-5.96)	(2.08-6.55)	(2.25-6.44)	(2.82-5.62)	(1.96-6.18)	(2.12-6.08)	(2.91-5.79)	(2.02-6.37)	(2.18-6.26)
50-54 y	4.07	3.57	3.67	3.83	3.36	3.45	3.95	3.47	3.56
	(2.87-5.72)	(2.00-6.30)	(2.17-6.18)	(2.71-5.39)	(1.88-5.94)	(2.04-5.84)	(2.79-5.56)	(1.94-6.12)	(2.10-6.01)
55-59 y	3.85	3.38	3.48	3.62	3.18	3.28	3.74	3.28	3.38
	(2.72-5.41)	(1.89-5.96)	(2.07-5.84)	(2.56-5.10)	(1.78-5.62)	(1.95-5.51)	(2.64-5.26)	(1.84-5.79)	(2.01-5.68)
60-64 y	3.61	3.17	3.27	3.40	2.98	3.08	3.50	3.07	3.17
	(2.55-5.08)	(1.77-5.59)	(1.96-5.47)	(2.40-4.78)	(1.67-5.27)	(1.84-5.16)	(2.47-4.93)	(1.72-5.43)	(1.90-5.31)
65-69 y	3.36	2.95	3.05	3.17	2.78	2.88	3.26	2.86	2.96
	(2.37-4.74)	(1.65-5.22)	(1.83-5.10)	(2.23-4.47)	(1.55-4.92)	(1.72-4.81)	(2.30-4.60)	(1.60-5.07)	(1.77-4.95)
Overall (5-69 y)	5.56	5.62	5.61	5.22	5.23	5.23	5.39	5.43	5.42
	(4.01-7.65)	(3.22-9.56)	(3.35-9.26)	(3.76-7.19)	(3.00-8.93)	(3.11-8.66)	(3.89-7.42)	(3.11-9.25)	(3.23-8.97)
**Ever asthma (%, 95% CI)**									
5-9 y	24.71	16.00	16.93	27.02	17.69	18.70	25.84	16.82	17.79
	(20.28-29.73)	(12.85-19.76)	(13.64-20.82)	(22.31-32.32)	(14.26-21.74)	(15.13-22.89)	(21.27-30.99)	(13.53-20.72)	(14.36-21.82)
10-14 y	22.44	14.38	15.28	24.61	15.93	16.92	23.50	15.13	16.07
	(18.32-27.17)	(11.50-17.83)	(12.26-18.88)	(20.20-29.62)	(12.79-19.67)	(13.63-20.81)	(19.24-28.37)	(12.12-18.72)	(12.92-19.81)
15-19 y	16.40	10.23	10.94	18.13	11.39	12.18	17.24	10.79	11.54
	(13.21-20.19)	(8.10-12.83)	(8.69-13.68)	(14.65-22.21)	(9.04-14.24)	(9.71-15.18)	(13.91-21.18)	(8.56-13.51)	(9.18-14.41)
20-24 y	13.32	8.19	8.85	14.78	9.15	9.87	14.03	8.66	9.34
	(10.65-16.54)	(6.46-10.34)	(6.99-11.13)	(11.85-18.28)	(7.23-11.51)	(7.82-12.38)	(11.23-17.38)	(6.83-10.91)	(7.39-11.74)
25-29 y	11.61	7.09	7.74	12.91	7.93	8.62	12.24	7.50	8.17
	(9.25-14.49)	(5.57-8.97)	(6.10-9.77)	(10.31-16.05)	(6.24-10.01)	(6.81-10.85)	(9.76-15.24)	(5.90-9.48)	(6.45-10.29)
30-34 y	10.55	6.41	7.01	11.74	7.17	7.81	11.12	6.78	7.40
	(8.38-13.20)	(5.03-8.13)	(5.52-8.87)	(9.35-14.64)	(5.64-9.07)	(6.16-9.86)	(8.84-13.89)	(5.33-8.59)	(5.83-9.35)
35-39 y	9.92	6.01	6.65	11.05	6.73	7.41	10.47	6.36	7.02
	(7.87-12.44)	(4.71-7.63)	(5.23-8.42)	(8.79-13.81)	(5.29-8.53)	(5.84-9.36)	(8.31-13.10)	(5.00-8.07)	(5.53-8.88)
40-44 y	9.62	5.82	6.49	10.72	6.52	7.24	10.16	6.17	6.86
	(7.62-12.07)	(4.56-7.39)	(5.11-8.22)	(8.52-13.41)	(5.12-8.26)	(5.71-9.15)	(8.06-12.72)	(4.84-7.83)	(5.40-8.68)
45-49 y	9.56	5.78	6.48	10.66	6.48	7.23	10.10	6.13	6.85
	(7.58-12.00)	(4.53-7.35)	(5.09-8.21)	(8.47-13.33)	(5.09-8.22)	(5.69-9.13)	(8.02-12.66)	(4.81-7.78)	(5.39-8.67)
50-54 y	9.70	5.87	6.61	10.81	6.57	7.37	10.25	6.22	6.99
	(7.69-12.16)	(4.60-7.46)	(5.20-8.37)	(8.59-13.51)	(5.16-8.33)	(5.81-9.31)	(8.13-12.83)	(4.88-7.90)	(5.50-8.84)
55-59 y	9.97	6.04	6.90	11.11	6.76	7.69	10.54	6.41	7.30
	(7.91-12.50)	(4.74-7.67)	(5.43-8.72)	(8.83-13.88)	(5.31-8.57)	(6.07-9.71)	(8.37-13.19)	(5.03-8.13)	(5.75-9.22)
60-64 y	10.34	6.27	7.24	11.51	7.02	8.08	10.93	6.66	7.67
	(8.21-12.94)	(4.92-7.96)	(5.71-9.15)	(9.16-14.36)	(5.52-8.89)	(6.38-10.19)	(8.69-13.67)	(5.23-8.44)	(6.05-9.68)
65-69 y	10.74	6.53	7.59	11.96	7.31	8.48	11.38	6.94	8.05
	(8.54-13.44)	(5.13-8.28)	(5.98-9.57)	(9.53-14.90)	(5.75-9.25)	(6.70-10.67)	(9.06-14.20)	(5.45-8.79)	(6.36-10.15)
Overall (5-69 y)	12.74	8.69	9.32	14.06	9.58	10.28	13.39	9.13	9.79
	(10.21-15.77)	(6.88-10.91)	(7.40-11.67)	(11.31-17.34)	(7.61-11.99)	(8.18-12.82)	(10.75-16.54)	(7.24-11.44)	(7.79-12.24)

HIC – high-income country, LMIC – low- and middle-income country, CI – confidence interval

The prevalence estimates of current wheezing and ever wheezing both peaked at the ages of 10-14 years. For current wheezing, the prevalence increased from 11.77% (95% CI = 9.43-14.58) in children aged 5-9 years to 12.43% (95% CI = 9.97-15.38) in those aged 10-14 years, and then slowly decreased to 11.04% (95% CI = 8.66-13.98) among elderly persons aged 65-69 years. In 2019, the overall prevalence of current wheezing was 11.45% (95% CI = 9.10-14.32) worldwide, and was slightly higher in HICs (12.14%, 95% CI = 9.76-15.01) than in LMICs (11.32%, 95% CI = 8.98-14.19). For ever wheezing, the prevalence estimates increased from 23.35% (95% CI = 18.91-28.46) in children aged 5-9 years to 25.41% (95% CI = 20.68-30.80) in children aged 10-14 years, then decreased to 14.30% (95% CI = 11.19-18.10) in people aged 30-34 years, but then started to gradually increase to 19.12% (95% CI = 15.00-24.06) in those aged 65-69 years. In 2019, the prevalence of ever wheezing was 17.93% (95% CI = 14.24-22.32) worldwide, and it was higher in HICs (19.06%, 95% CI = 15.02-23.87) than in LMICs (17.72%, 95% CI = 14.09-22.04).

The prevalence of current asthma and ever asthma was the highest in younger children aged 5-9 years. For current asthma, the prevalence was 13.41% (95% CI = 8.14-21.41) in children aged 5-9 years and 10.95% (95% CI = 6.59-17.75) in those aged 10-14 years. Thereafter, the prevalence continuously decreased to 2.96% (95% CI = 1.77-4.95) in the elderly aged 65-69 years. In 2019, the prevalence of current asthma was 5.42% (95% CI = 3.23-8.97) globally and marginally higher in LMICs (5.43%, 95% CI = 3.11-9.25) than in HICs (5.39%, 95% CI = 3.89-7.42). Similarly, the prevalence of ever asthma peaked at 17.79% (95% CI = 14.36-21.82) in children aged 5-9 years and then decreased to 6.85% (95% CI = 5.39-8.67) in people aged 45-49 years, only to gradually increase to 8.05% (95% CI = 6.36-10.15) in persons aged 65-69 years. In 2019, the prevalence of ever asthma was 9.79% (95% CI = 7.79-12.24) globally and was much higher in HICs (13.39%, 95% CI = 10.75-16.54) than in LMICs (9.13%, 95% CI = 7.24-11.44).

### Global cases of current wheezing, ever wheezing, current asthma and ever asthma in 2019

By applying the global demographic data, we estimated that there were 754.6 million aged 5-69 years (95% CI = 599. 7-943.4) with current wheezing, 1181.3 million (95% CI = 938.0-1471.0) with ever wheezing, 357.4 million (95% CI = 213.0-590.8) with current asthma, and 645.2 million (95% CI = 513.1-806.2) with ever asthma in 2019. More than four-fifths of people with current wheezing (83.5%), ever wheezing (83.4%), and current asthma (84.5%) were living in LMICs, while 78.7% of people that had ever been diagnosed with asthma were in LMICs (see Table S7 and Figure S3 in the **Online Supplementary Document**).

### The regional and national distribution of current wheezing and ever asthma in 2019

**Figure 3:**
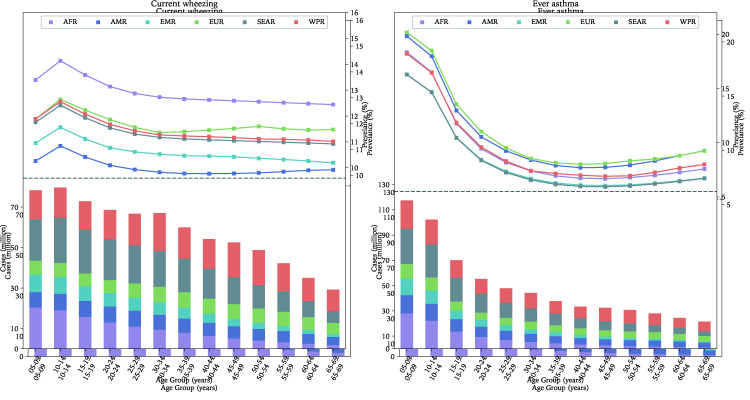
The age-specific prevalence (points) and number of cases (bar) of current wheezing or ever asthma in 2019, by geographic region. AFR – African Region, AMR – Region of the Americas, SEAR – South-East Asia Region, EUR – European Region, EMR – Eastern Mediterranean Region, WPR – Western Pacific Region.

**Figure 4:**
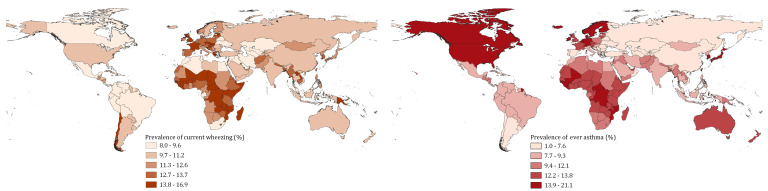
The national prevalence of current wheezing or ever asthma in 2019.

As shown in **Figures 3-4** and detailed in Table S8 in the **Online Supplementary Document**, the SEAR had the largest global cases of current wheezing (200.57million, 95% CI = 159.02-251.29) in 2019, while the EMR had the least (65.91 million, 95% CI = 52.39-82.37). The age group that contributed the most cases of current wheezing was 5-9 years in the AFR and EMR, 10-14 years in the AMR and SEAR, and 30-34 years in the EUR and WPR. The overall prevalence of current wheezing in people aged 5-69 years was the highest in the AFR at 13.18% (95% CI = 10.47-16.47) and the lowest in the AMR at 10.03% (95% CI: 8.01-12.49). The ten countries with the most cases of current wheezing in descending order were China, India, United States of America, Indonesia, Brazil, Pakistan, Nigeria, Bangladesh, Russian Federation, and Ethiopia, where the total cases were 439.2 million and accounted for more than half (58.2%) of the global cases of current wheezing in 2019 (see Table S9 in the **Online Supplementary Document**).

For ever asthma, the WPR had the most cases (156.55 million, 95% CI = 124.2-196.14), whereas the EMR had the smallest share (57.34 million, 95% CI = 45.59-71.65) in 2019. The age group that contributed the most cases of ever asthma was 5-9 years across the six WHO regions. The estimated prevalence of ever asthma in people aged 5-69 years was the highest in the AFR (11.31%, 95% CI = 8.99-14.12) and the lowest in the SEAR (8.80%, 95% CI = 6.98-11.03). In 2019, more than half (56.4%, 363.6 million) of the global cases of ever asthma were in ten countries, namely India, China, United States of America, Nigeria, Indonesia, Pakistan, Japan, Brazil, Bangladesh, and Germany, in descending order.

Pooled ORs of associated factors of current wheezing and ever asthma across HICs and LMICs are shown in Table S10 in the **Online Supplementary Document** and additional notes on associated factors are in **Box 2**.

Box 2 **Asthma risk factors**

Our findings confirm an association of asthma symptoms with a personal or family history of atopy or allergies, including allergic rhinitis and eczema. This would continue to be an important risk across HICs and LMICs and an important consideration for clinical management. Although not specific to any settings in particular, reduced exposures to unhygienic areas, commensals and non-pathogenic microbes in early life are believed to prompt low microbial diversity in the respiratory tract – a likely factor for developing IgE-mediated immune response following exposures to airborne triggers like grass pollens, animal dander, and fungal spores [20,21].

Tobacco smoking is a major risk factor for asthma across all age groups globally, one study observed [22]. While the burden of asthma attributable to tobacco smoking may have been declining over the last three decades, it still accounts for over 9% of the global disability-adjusted life years (DALYs) from asthma [22,23]. The high burden of asthma (15% of global DALYs) reported in Europe over the years [24] has been thought to be smoking-related, particularly to second-hand smoking, which is an important risk among women and children [23]. Although data gaps have limited the understanding of the contribution of second-hand smoking to asthma burden across HICs and LMICs in recent studies, it was estimated that there were about 40 000 asthma deaths due to second-hand smoking in 2004m with 28% occurring in children and 47% in women [24]. Parental smoking is closely related, with estimates suggesting over 50% of exposed children developing asthma symptoms, and this persisting up to the age of six for those exposed during pregnancy and/or the first year of life [25] (additional notes on associated factors are in the **Online Supplement Document**, page 66).

## DISCUSSION

To the best of our knowledge, this study provides the first comprehensive global, regional, and national analysis of asthma based on four common epidemiological definitions before the COVID-19 pandemic. This study offers a broad perspective on the disease necessary to inform clinical management, research priorities, intervention focus, and health investments across world settings.

Asthma is generally prevalent among children and our findings are aligned with emerging reports that the prevalence of asthma symptoms in young adults and through middle-aged is gradually increasing [11,12]. In the 2021 GAN Phase I study (a follow-up to the ISAAC phase 3), the global prevalence of current wheezing in 2017 was 9.9% among children aged 6-7 years and 11.0% among adolescents aged 13-14 years, while the prevalence for ever asthma was 9.0% among the former and 11.0% among the latter, respectively [13]. Our estimates of current wheezing in 2019 among 5-9 years (11.8%, 95% CI = 9.4-14.6) and 10-14 years (12.4%, 95% CI = 10.0-15.4) age groups are comparable with the reported GAN 1 prevalence rates among children and adolescents, respectively. Although our estimates of ever asthma in both age groups were relatively higher, likely explanations include 1) a global increase in population size of 5-year age cohorts over time, 2) improvement of access to care worldwide, 3) improved medical care for respiratory conditions, 4) increased awareness and diagnosis of wheeze, asthma and asthma-chronic obstructive pulmonary disease (COPD) syndrome, 5) changes in environmental risk factors over time, and 6) the stochastic nature of global data on asthma prevalence.

Conducting long-term cohort studies among children is challenging, which makes studying trends in asthma prevalence in children relatively difficult, limiting understanding of the global time trends [14]. With socio-demographic changes and worsening exposure to risks over time, the prevalence and absolute number of cases may be growing. Previous history of allergies and asthma in childhood persisting through adulthood may have also translated to this increase in prevalence among young adults and the middle-aged [14]. It should be noted that the possibility of asthma-COPD symptoms overlapping, or even the misdiagnosis of asthma with advancing age, cannot be ruled out as a further source of variation in estimates. For example, the GBD collaborators used data from numerous sources to report that there were 358 million cases of asthma in 2015 [15] and 339 million in 2016 [16]. However, a substantial reduction in global cases to 273 million in 2017 and 262 million in 2019 appears to be an important decreasing trend [1]. Some authors have raised concerns that the large variations between the GBD estimates may be mainly due to changes in analytical methods rather than contributions from new large-scale studies [17]. Furthermore, we opine that the GBD definition of asthma as “reported diagnosis combined with wheezing in the past 12 months” may have excluded asthma cases with stand-alone asthmatic symptoms, which in part explains why their estimates are lower than ours.

The geographic pattern of asthma prevalence observed in various ecological-economic analyses has consistently implied a higher prevalence in HICs compared to LMICs [18]. We also observed such disparity, especially for current wheezing and ever asthma. We would expect that mild to moderate symptoms of asthma would tend to be underdiagnosed across many LMICs. A relatively higher presence of poor housing conditions, with over-crowding, damp environments, and second-hand smoking among children in many houses, would imply a rising prevalence of asthma symptoms in LMICs. Moreover, severe symptoms and complications from asthma appear to be more prominent in LMICs, especially among the elderly [18], possibly reflecting missed diagnosis, under-treatment, and poorer overall response to the disease in these settings.

When exploring the specific regional variations in prevalence, current wheezing rates appear to be quite similar across geographic regions except for the AFR (13.2%), ranging from 10.0% in the AMR to 11.6% in the EUR. In addition, while we observed that the EUR (20.2%) and AMR (19.8%) returned the highest prevalence of ever asthma mainly among children, the overall prevalence of ever asthma among persons aged 5-69 years was still the highest in the AFR at 11.3%. The higher estimates of current wheezing and ever asthma in AFR may be explained by several episodes of respiratory infections in many settings, which are often very severe and poorly treated, resulting in repeated lung injuries that manifest in later life with breathing difficulties such as asthma and other chronic respiratory conditions.

This study offers opportunities to better understand the epidemiology of a rather poorly researched disease in many settings, and is complementary to the existing ISAAC studies [18], GBD reports [1], and recently, the GAN estimation of asthma [13]. However, limitations are still present. First, the under- or over- estimation may be an important limitation. The agreement between the definitions of asthma employed in some meta-analyses was just above 60%, with many asthma cases fitting as controls [14]. Moreover, we acknowledge that the older age group may be subject to recall bias for ever asthma, as persons may not recall having asthma in early childhood, especially if the episodes were not severe. Differentiating asthma from other respiratory comorbidities (like COPD) may lead to an overestimation of ever asthma in adults. Establishing national asthma prevalence estimates was also quite challenging; there is considerable uncertainty associated with our national-level estimates, as data, particularly on associated factors, were not available from all countries and across different age groups and sexes. We also acknowledge the limitations of applying the UNPD demographic data, which is often questioned for its accuracy in LMICs.

The need to acquire standardised data over time from well-conducted studies across countries tops the list of priorities towards improving the global, regional, and national estimates of asthma burden in the future [2]. Besides agreeing on definitions and protocols, standardised collation of hospital and administrative data across different settings will help in assessing the impact of asthma on health care utilisation. The WHO recognised the role of health care services in optimising asthma control by providing relevant information about disease progression and treatment, including those related to professional advice, home monitoring of symptoms and self-management [19]. It will also be important for LMIC health systems to invest more in primary care respiratory services to meet the needs of this large segment of the population, especially for Africa and SEAR.

The contributions of environmental factors to asthma is poorly understood in many countries. Research on various environmental factors, particularly air pollution in different contexts, could both explore genetic interactions and assist our understanding of the determinants of asthma. Given its variable nature, diagnosing asthma using lung function tests remains an issue in epidemiologic and clinical settings. It also highlights a need to improve diagnostic skills across primary care settings, particularly in LMICs.

## CONCLUSIONS

Asthma, based on whichever definition is used, was more common than previously estimated in the period before the COVID-19 pandemic. Our study should be particularly useful for planning a national and sub-national response to asthma and will complement other international research efforts.

## Additional material


Online Supplementary Document
