# Supplementary material for: Global, regional, and national prevalence of asthma in 2019: a systematic analysis and modelling study
Source: J Glob Health. 2022 Jun 29;12:04052. doi: 10.7189/jogh.12.04052 (PMC9239324; doi:10.7189/jogh.12.04052)
Supplement: Online Supplementary Document [file jogh-12-04052-s001.pdf]

Supplement

Appendix 1. Searches, world regions, countries and territories 2

Table S1. Search strategy to identify studies reporting the prevalence of asthma in the general population 2

Table S2. Country or territory list in World Health Organization and World Bank regions 3

Appendix 2. eMethods: Detailed description of the global, regional and national estimation of asthma prevalence ....

4 Figure S1. Contribution of data sources across world regions 7

Figure S2. The prevalence of current wheezing, ever wheezing, current asthma and ever asthma, by age and sex group, based on contributing datapoints from included articles 8

Table S3. The age- and sex-adjusted effects of study-level variables on current wheezing, ever wheezing, current asthma and ever asthma 9

Appendix 3. Modelling the global prevalence of asthma 12

Table S4. Models for constructing the prevalence of current wheezing, ever wheezing, current asthma and ever asthma 12

Appendix 4. Characteristics of the included studies 13

Table S5. Characteristics of included studies with prevalence data for sample population 13

Table S6. List of the included articles 37

Appendix 5. Estimation of the regional and national numbers of people with asthma in 2019 47

Figure S3. The age-specific prevalence (points) and number of cases (bar) of asthma in 2019, by income 47

Table S7. The estimated numbers of people with current wheezing, ever wheezing, current asthma and ever asthma in HICs, LMICs and worldwide in 2019, by age group and sex 48

Table S8. Estimated numbers of people with current wheezing or ever asthma in World Bank income and World Health Organization combined regions in 2019, by age group 53

Table S9. Estimated prevalence and number of people aged 0-79 years living with current wheezing or ever asthma in 201 countries and territories in 2019 55

Table S10. Pooled odds ratios of associated factors of current wheezing and ever asthma by World Bank region 63

Additional notes on associated factors of asthma 67

References 68

Appendix 1. Searches, world regions, countries and territories

Table S1. Search strategy to identify studies reporting the prevalence of asthma in the general population

	**Medline (n=7,877)**
1	exp asthma/ or exp asthma, aspirin-induced/ or exp asthma, exercise-induced/ or exp asthma, occupational/ or exp status asthmaticus/ or *bronchial hyperreactivity/ or *bronchial spasm/
2	exp morbidity/ or exp incidence/ or exp prevalence/ or *mortality/
3	*risk factors/
4	(burden adj3 disease).mp. [mp=title, abstract, original title, name of substance word, subject heading word, floating sub-heading word, keyword heading word, organism supplementary concept word, protocol supplementary concept word, rare disease supplementary concept word, unique identifier, synonyms]
5	2 or 3 or 4
6	1 and 5
7	limit 6 to (humans and yr="1990 -Current")
	**EMBASE (n= 10,044)**
1	exp asthma/ or exp wheezing/
2	exp prevalence/ or *epidemiology/
3	exp morbidity/ or exp mortality/
4	(burden adj3 disease).mp. [mp=title, abstract, original title, name of substance word, subject heading word, floating sub-heading word, keyword heading word, organism supplementary concept word, protocol supplementary concept word, rare disease supplementary concept word, unique identifier, synonyms]
5	2 or 3 or 4
6	1 and 5
7	limit 6 to (humans and yr="1990 -Current")
	**Global Health (n=4,451)**
1	exp epidemiology/ or exp morbidity/ or exp mortality/
2	exp risk factors/
3	exp asthma/
4	exp wheezing/
5	1 or 2
6	3 or 4
7	7 and 8
8	limit 7 to yr="1990 -Current"
	**CINAHL (n=3,491)**
	(MM asthma* OR MM wheezing*) AND ((MM "Prevalence") OR (MM "Epidemiology") OR (MH "Morbidity+") OR (MH "Mortality+")) Limiters: Publication Year: 1990-2019

Table S2. Country or territory list in World Health Organization and World Bank regions

**High-income countries (HIC)**

Region of the Americas (AMR)	Antigua and Barbuda, Aruba, Bahamas, Barbados, Curaçao, Guadeloupe, Martinique, Puerto Rico, Trinidad and Tobago, United States Virgin Islands, Argentina, Chile, French Guiana, Uruguay, Canada, United States of America
European Region (EUR)	Cyprus, Israel, Czechia, Hungary, Poland, Slovakia, Channel Islands, Denmark, Estonia, Finland, Iceland, Ireland, Latvia, Lithuania, Norway, Sweden, United Kingdom, Croatia, Greece, Italy, Malta, Portugal, Slovenia, Spain, Austria, Belgium, France, Germany, Luxembourg, Netherlands, Switzerland
Eastern Mediterranean Region (EMR)	Bahrain, Kuwait, Oman, Qatar, Saudi Arabia, State of Palestine, United Arab Emirates
Western Pacific Region (WPR)	China Hong Kong SAR, China Macao SAR, China Taiwan Province, Japan, Republic of Korea, Brunei Darussalam, Singapore, Australia, New Zealand, New Caledonia, Guam
**Low-income and middle-income countries (LMIC)**	
African Region (AFR)*	Burundi, Comoros, Eritrea, Ethiopia, Kenya, Madagascar, Malawi, Mauritius, Mayotte, Mozambique, Réunion, Rwanda, South Sudan, Uganda, United Republic of Tanzania, Zambia, Zimbabwe, Angola, Cameroon, Central African Republic, Chad, Congo, Democratic Republic of the Congo, Equatorial Guinea, Gabon, Sao Tome and Principe, Algeria, Western Sahara, Botswana, Lesotho, Namibia, South Africa, Swaziland, Benin, Burkina Faso, Cabo Verde, Côte d'Ivoire, Gambia, Ghana, Guinea, GuineaBissau, Liberia, Mali, Mauritania, Niger, Nigeria, Senegal, Seychelles*, Sierra Leone, Togo
Region of the Americas (AMR)	Cuba, Dominican Republic, Grenada, Haiti, Jamaica, Saint Lucia, Saint Vincent and the Grenadines, Belize, Costa Rica, El Salvador, Guatemala, Honduras, Mexico, Nicaragua, Panama, Bolivia (Plurinational State of), Brazil, Colombia, Ecuador, Guyana, Paraguay, Peru, Suriname, Venezuela (Bolivarian Republic of)
South-East Asia Region (SEAR)	Dem. People's Republic of Korea, Bangladesh, Bhutan, India, Maldives, Nepal, Sri Lanka, Indonesia, Myanmar, Thailand, Timor-Leste
European Region (EUR)	Kazakhstan, Kyrgyzstan, Tajikistan, Turkmenistan, Uzbekistan, Armenia, Azerbaijan, Georgia, Turkey, Belarus, Bulgaria, Republic of Moldova, Romania, Russian Federation, Ukraine, Albania, Bosnia and Herzegovina, Montenegro, Serbia, TFYR Macedonia
Eastern Mediterranean Region (EMR)	Djibouti, Somalia, Egypt, Libya, Morocco, Sudan, Tunisia, Afghanistan, Iran (Islamic Republic of), Pakistan, Iraq, Jordan, Lebanon, Syrian Arab Republic, Yemen
Western Pacific Region (WPR)	China, Mongolia, Cambodia, Lao People's Democratic Republic, Malaysia, Philippines, Viet Nam, Fiji, Papua New Guinea, Solomon Islands, Vanuatu, Kiribati, Micronesia (Fed. States of), French Polynesia, Samoa, Tonga

Appendix 2. eMethods: Detailed description of the global, regional and national estimation of asthma prevalence

This section is a supplement to the Methods part in the main text.

According to our systematic review and data extraction approach, multiple data points (agespecific or sex-specific prevalence) were extracted from the same individual articles. To account for the clustering of multiple data points from the same article, and multiple articles from the same country, we used a multilevel multivariable mixed-effects meta-regression and took study and country identification as the random-effects. Given that the prevalence of asthma

= number of asthma cases/number of participants, we stabilized the prevalence with the logit link as follows:

$$\text{prevalence of asthma} = P = (asthma\ cases)/(sample\ size)$$

$$\text{logit}(P) = \ln(P/[1-P]) = \ln(odds) = \alpha + \beta_1 * x_1 + \beta_2 * x_2 + \cdots + u_i$$

where α is the intercept term, β is coefficient, and x is variable.

First, we assessed the age- and sex-adjusted effects of cluster-level variables $x_1 - x_n$, namely year of investigation, WB income region, setting (urban, rural and mixed) stratified by WB region, WHO region, and SDI by WB region (see **Table S3** for more details). For current wheezing, we found that the prevalence significantly correlated with age, was higher in males (than in females), urban areas in LMICs (than in rural areas in HICs), AMR and EMR (than in WPR). For ever wheezing, we found that it was associated with age, more common in males (than in females) and in AMR (than in WPR). In LMICs, the prevalence of ever wheezing was higher in rural areas than in urban settings, decreased with higher SDI; while in HICs, the prevalence of ever wheezing increased with higher SDI. For current asthma, the prevalence significantly correlated with age, was higher in males, increased over time. In HICs, current asthma was more common in urban areas (than in rural areas), and increased with higher SDI. Similarly, the prevalence of ever asthma was significantly associated with age, higher in males (than in females), in SEAR, EUR and EMR (than in WPR). In both HICs and LMICs, ever asthma was more common in urban areas than in rural areas. At higher SDIs, the prevalence of ever asthma increased in HICs, but decreased in LMICs.

Based on the abovementioned explorations, we fitted a multilevel multivariable mixed-effects meta-regression, with age, sex and calendar year as fixed-effect variables and with study and country identification as the random-effect (μ_i). The nonlinear relation between age and asthma prevalence was modelled using a restricted cubic spline, with knots being selected by visual inspection at the inflection points of the curve. The sex-specific relation of age and asthma prevalence is demonstrated in **Figure S2**. Based on data points from all included articles, we constructed four models for generating the prevalence of current wheezing, ever wheezing, current asthma and ever asthma. Those models were constructed separately for HICs and LMICs.

$$\text{logit}(P) = \alpha + \beta_1 * Age_1 + \beta_2 * Age_2 + \beta_3 * Age_3 + \beta_4 * Age_4 + \beta_5 * Female\ proportion + \beta_6 * Investigation\ year + \beta_6 * WB + u_i$$

then,

$$P = \frac{e^{\alpha+\beta_1*Age_1+\beta_2*Age_2+\beta_3*Age_3+\beta_4*Age_4+\beta_5*Female\ proportion+\beta_6*Investigation\ year+\beta_6*WB+u_i}}{1 + e^{\alpha+\beta_1*Age_1+\beta_2*Age_2+\beta_3*Age_3+\beta_4*Age_4+\beta_5*Female\ proportion+\beta_6*Investigation\ year+\beta_6*WB+u_i}}$$

Where Age_1- Age_4 are variables generated in the process of fitting cubic splines; WB refers to income regions of HICs and LMICs, and u_i represents the variance of random-effects.

Based on the above models (**Table S4**), the age- and sex- prevalence of current wheezing, ever wheezing, current asthma and ever asthma in HICs, LMICs and worldwide for the year 2019 was estimated (**Table 1** in the maintext).

Statistical step two: Estimation of the global numbers of people with asthma in 2019

The numbers of people aged 5-69 years who were affected by of current wheezing, ever wheezing, current asthma and ever asthma were established by applying the estimated age-, sex-prevalence

to the corresponding demographic data in 2019 (**Table S7**). The overall cases of current wheezing and ever asthma in HICs and LMICs were called "current wheezing HIC envelope", "current wheezing LMIC envelope", "ever asthma HIC envelope" and "ever asthma LMIC envelope" respectively, and were used as the basses for the following regional and national estimations of current wheezing and ever asthma cases.

Statistical step three: Estimation of the regional numbers of people with current wheezing or ever asthma in 2019

We used a "risk factor-based model" distributing the global cases of current wheezing and ever asthma into ten WB-WHO regions (**Table S8**).

$$N_{WHO-WB\,region} = (Pop_{WHO-WB\,region}) * (Prev_{asthmaHIC/LMIC}) * (1 + \sum_{RF_1}^{RF_n} [(Prev_{RFWHO-WB\,region} - Prev_{RFHIC/LMIC}) * (OR_{RFHIC/LMIC} - 1)])$$

Where $N_{WHO-WB\,region}$ refers to the cases of current wheezing or ever asthma in each of the ten WHO-WB regions, $Pop_{WHO-WB\,region}$ is the age- and sex-specific de-facto population of people in 2019 in each WHO-WB region, $Prev_{asthmaHIC/LMIC}$ indicates the estimated age- and sexspecific prevalence of current wheezing or ever asthma in 2019 in HICs or LMICs where the corresponding WHO-WB region belongs. RF_1-RF_n are the selected risk factors. Current smoking and biomass exposure were included in the model for current wheezing, while rural setting, current smoking, biomass exposure and SDI were included in the model for ever asthma.The percentage of population residing in rural areas in 2020 was obtained from UNPD.(1) The prevalence of current smoking in 2018, the proportion of population with primary reliance on polluting fuels and technologies for cooking in 2018 were obtained from the WHO Global Health Observatory data repository;(2) SDI in 2019 was obtained from the GBD 2019 Study.(3)

$Prev_{RFWHO-WB\,region}$ is the prevalence of selected risk factors in each WHO-WB region and $Prev_{RFHIC/LMIC}$ is the prevalence of selected risk factors in HICs or LMICs where this WHO-WB region stands. $OR_{RFHIC/LMIC}$ is the estimated/synthesized OR of a specific risk factor in HICs or LMICs where the selected WHO-WB region locates.

Finally, the regional cases of current wheezing and ever asthma were adjusted by multiplying an "adjustment index" to make sure that the sum of regional cases exactly fitted the "current wheezing HIC envelope", "current wheezing LMIC envelope", "ever asthma HIC envelope" and "ever asthma LMIC envelope". The regional prevalence of current wheezing and ever asthma was respectively calculated by dividing the estimated numbers of people with current wheezing and ever asthma by the corresponding population in each region in 2019.

Statistical step four: Estimation of the national numbers of people with current wheezing or ever asthma

Similarly, the national numbers of people with current wheezing and ever asthma were estimated with the below formula (**Table S9**):

$$N_{country} = Pop_{country} * (Prev_{asthmaHIC/LMIC}) * (1 + \sum_{RF_1}^{RF_n} [(Prev_{RFcountry} - Prev_{RFWHO-WB\,region}) * (OR_{RFHIC/LMIC} - 1)])$$

Where $N_{country}$ is the cases of current wheezing or ever asthma, and $Pop_{country}$ is the de-facto population size in 201 countries and territories in 2019. $Prev_{asthmaHIC/LMIC}$ indicates the prevalence of current wheezing or ever asthma in HICs or LMICs where the target country belongs. RF_1-RF_n

are the same risk factors adopted in the regional case estimations for current wheezing or ever asthma. $Prev_{RFcountry}$ and $Prev_{RFWHO-WB\ region}$ are the prevalence rates of risk factors in each country and territory and in its corresponding WHO-WB region. $OR_{RFHIC/LMIC}$ is the estimated OR of the selected risk factors in HIC or LMIC.

Finally, the national cases of current wheezing and ever asthma were also adjusted by multiplying an “adjustment index” to make sure that the sum of national cases equalled to the abovementioned WHO-WB “envelopes”. The national prevalence of current wheezing and ever asthma was generated by dividing the estimated cases of current wheezing and ever asthma by the corresponding national population in 2019.

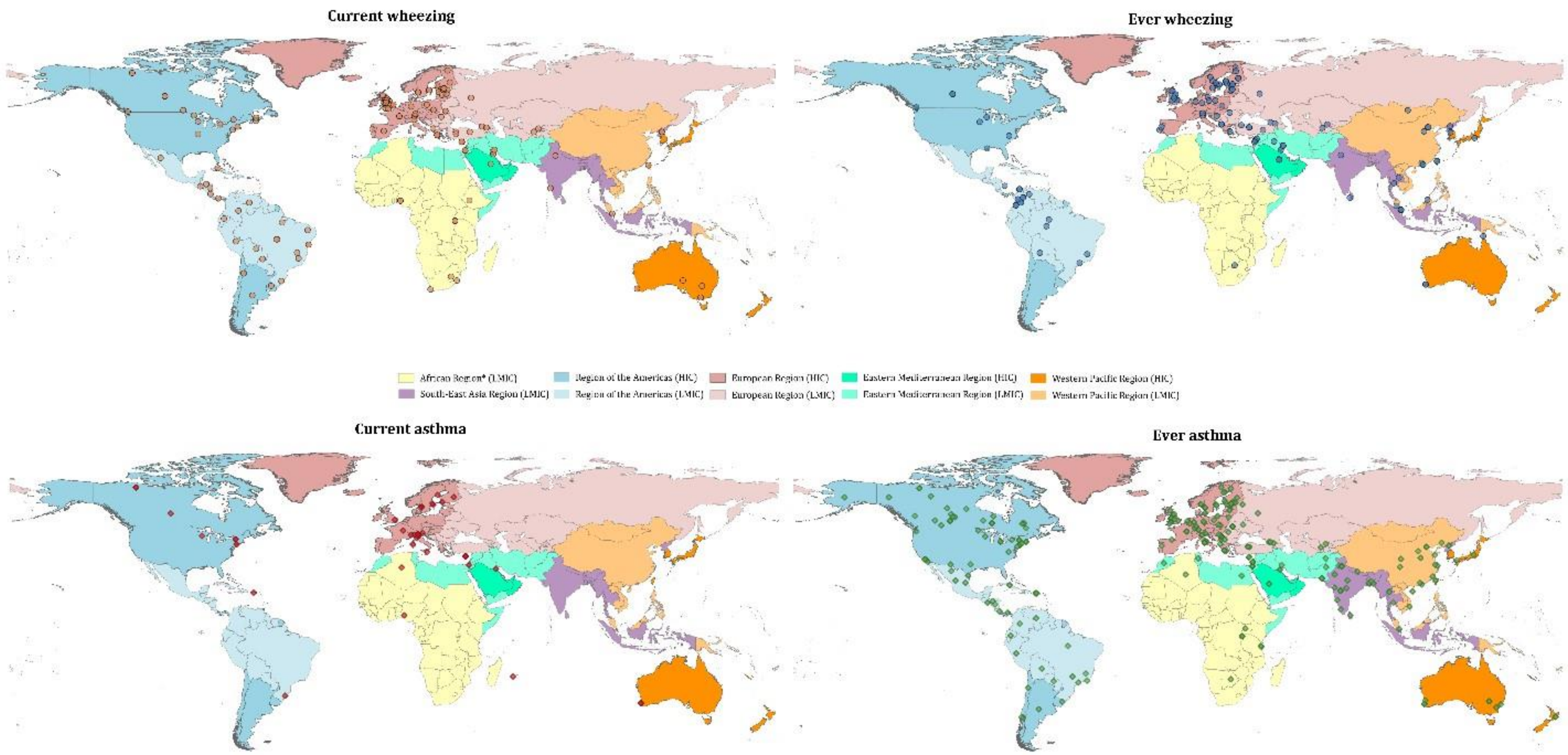

Figure S1. Contribution of data sources across world regions

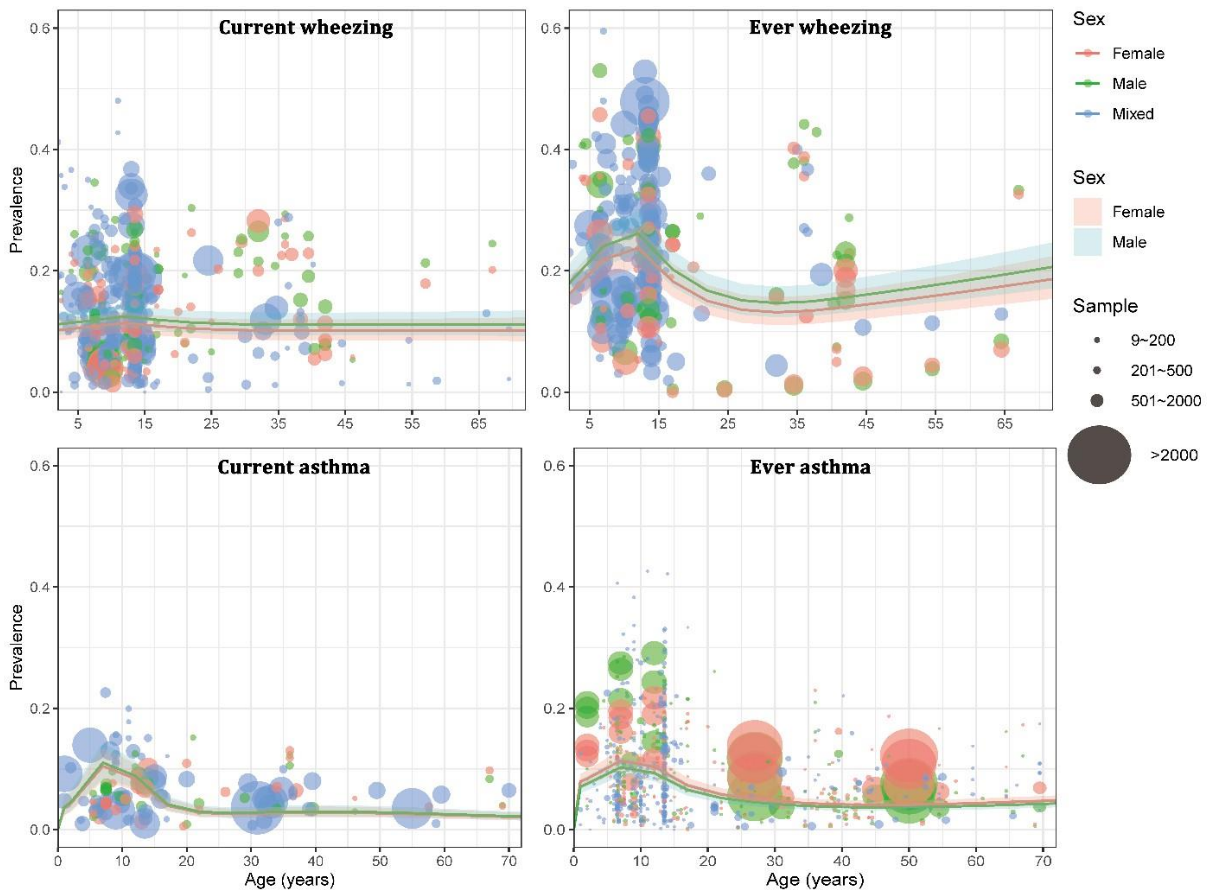

Figure S2. The prevalence of current wheezing, ever wheezing, current asthma and ever asthma, by age and sex group, based on contributing datapoints from included articles

Table S3. The age- and sex-adjusted effects of study-level variables on current wheezing, ever wheezing, current asthma and ever asthma

Variable		No. of articles	No. of data points	β (95% CI)	*P*-value
			**Current wheezing**		
**Age**					
	Age_1	146	449	0.012 ([-0.011]-0.035)	0.3070
	Age_2	146	449	0.086 ([-0.433]-0.604)	0.7459
	Age_3	146	449	-0.839 ([-2.419]-0.741)	0.2979
	Age_4	146	449	5.920 ([0.072]-11.769)	0.0472
**Female proportion**		146	449	-0.102 ([-0.121]-[-0.084])	<.0001
**Sex**#					
	Male	59	91		
	Female	59	90	-0.106 ([-0.124]-[-0.087])	<.0001
**Investigation year (per year increase)**		146	446	0.006 (0.003-0.009)	0.0002
**WB income region**					
	HICs	94	261		
	LMICs	58	188	-0.052 ([-0.383]-0.279)	0.7586

**Setting by WB**					
Overall	Urban	93	253		
	Rural	31	69	-0.099 ([-0.158]-[-0.039])	0.0011
	Urban HICs	54	143		
	Rural	17	33	-0.051 ([-0.134]-0.032)	0.2244
LMICs	Urban	41	110		
	Rural	15	36	-0.144 ([-0.229]-[-0.060])	0.0008
**WHO region**	WPR	26	75		
	AFR	11	38	0.227 ([-0.405]-0.858)	0.4821
	AMR	38	113	0.631 (0.086-1.175)	0.0232
	SEAR	8	16	0.174 ([-0.581]-0.928)	0.6519
	EUR	55	178	0.250 ([-0.198]-0.699)	0.2739
	EMR	10	29	0.739 (0.136-1.342)	0.0163
**SDI by WB (per 1% increase)**					
Overall	SDI	146	470	0.002 ([-0.401]-0.008)	0.5096
HICs	SDI	93	270	0.003 ([-0.004]-0.010)	0.4125
LMICs	SDI	58	200	0.003 ([-0.013]-0.020)	0.6878
**Ever wheezing**					
**Age**					
	Age_1	89	268	0.075 ([0.057]-0.094)	<.0001
	Age_2	89	268	-1.716 ([-2.322]-[-1.111])	<.0001
	Age_3	89	268	2.463 (0.248-4.678)	0.0293
	Age_4	89	268	13.347 (2.721-23.973)	0.0138
**Female proportion**		89	268	-0.127 ([-0.152]-[-0.102])	<.0001
**Sex**#					
	Male	34	53		
	Female	34	53	-0.133 ([-0.158]-[-0.109])	<.0001
**Investigation year (per year increase)**		89	267	0.004 ([-0.001]-0.010)	0.1344
**WB income region**					

**Variable**		**No. of articles**	**No. of data points**	**β (95% CI)**	***P*-value**
	HICs	51	132		
	LMICs	41	136	-0.091 ([-0.451]-0.270)	0.6216
**Setting**					
Overall	Urban	61	178		
	Rural	17	42	2.078 (1.909-2.246)	<.0001
HICs	Urban	33	90		
	Rural	7	14	0.040 ([-0.717]-0.797)	0.9178
LMICs	Urban	30	88		
	Rural	11	28	2.127 (1.958-2.295)	<.0001
**WHO regi n**					
	WPR	21	62		
	AFR	4	6	0.846 ([-0.275]-1.967)	0.1390
	AMR	20	61	0.835 ([0.282]-1.389)	0.0031
	SEAR	6	11	0.133 ([-0.590]-0.856)	0.7184

	EUR	31	104	0.374 ([-0.083]-0.831)	0.1091
	EMR	8	24	0.551 ([-0.049]-1.151)	0.0720
**SDI by WB**	**(per 1% increase)**				
Overall	SDI	89	267	0.005 ([-0.004]-0.013)	0.2650
HICs	SDI	51	131	0.024 (0.013-0.035)	<.0001
LMICs	SDI	41	136	-0.017 ([-0.032]-[-0.001])	0.0347
			**Current asthma**		
**Age**					
	Age_1	50	128	0.200 (0.141-0.259)	<.0001
	Age_2	50	128	-18.682 ([-22.497]-[-14.868])	<.0001
	Age_3	50	128	40.203 (32.228-48.178)	<.0001
	Age_4	50	128	-22.339 ([-26.623]-[-18.056])	<.0001
**Female proportion**		50	128	-0.059 ([-0.111]-[-0.008])	0.0249
**Sex#**					
	Male	20	32		
	Female	20	32	-0.066 ([-0.118]-[-0.014])	0.0122
**Investigation year (per year increase)**		50	126	0.021 (0.015-0.027)	<.0001
**WB incom ɛ region**					
	HICs	40	105		
	LMICs	11	23	-0.040 ([-0.700]-0.619)	0.9043
**Setting**					
Overall	Urban	32	72		
	Rural	9	21	-0.175 ([-0.321]-[-0.029])	0.0189
HICs	Urban	23	53		
	Rural	8	18	-0.156 ([-0.303]-[-0.009])	0.0380
LMICs	Urban	9	19		
	Rural	1	3	-1.360 ([-3.096]-0.376)	0.1248
**WHO region**					
	WPR	6	15		
	AFR	3	9	-0.673 ([-1.846]-0.500)	0.2605
	AMR	13	25	0.089 ([-0.716]-0.895)	0.8278
	SEAR	1	1	-1.212 ([-2.922]-0.498)	0.1649
	EUR	26	74	-0.622 ([-1.423]-0.179)	0.1278

Variable		No. of articles	No. of data points	β (95% CI)	*P*-value
	EMR	2	4	0.774 ([-0.548]-2.095)	0.2511
**SDI by WB (per 1% increase)**					
Overall	SDI	50	128	0.047 (0.030-0.063)	<.0001
HICs	SDI	40	105	0.059 (0.040-0.077)	<.0001
LMICs	SDI	11	23	0.055 ([-0.027]-0.136)	0.1910
			**Ever asthma**		
**Age**					
	Age_1	189	776	0.068 (0.068-0.069)	<.0001
	Age_2	189	776	-7.098 ([-7.172]-[-7.024])	<.0001
	Age_3	189	776	13.623 (13.469-13.777)	<.0001
	Age_4	189	776	-6.822 ([-6.913]-[-6.731])	<.0001
**Female proportion**		189	776	0.114 (0.112-0.116)	<.0001
**Sex#**					
	Male	77	188		
	Female	77	188	0.114 (0.112-0.116)	<.0001
**Investigation year (per year increase)**		188	768	0.054 (0.053-0.054)	<.0001
**WB incom ɜ region**					
	HICs	120	425		
	LMICs	74	351	-0.242 ([-0.567]-0.082)	0.1436
**Setting**					
Overall	Urban	122	429		
	Rural	37	92	-0.174 ([-0.229]-[-0.119])	<.0001
HICs	Urban	71	258		
	Rural	16	29	-0.132 ([-0.225]-[-0.039])	0.0055
LMICs	Urban	53	171		
	Rural	22	63	-0.279 ([-0.348]-[-0.210])	<.0001
**WHO region**					
	WPR	34	129		
	AFR	11	47	0.485 ([-0.274]-1.244)	0.2106
	AMR	52	191	-0.292 ([-0.686]-0.102)	0.1460
	SEAR	11	73	1.327 (0.917-1.738)	<.0001
	EUR	72	296	0.523 (0.133-0.913)	0.0086
	EMR	14	40	1.010 (0.619-1.400)	<.0001
**SDI by WB (per 1% increase)**					
Overall	SDI	188	768	0.157 (0.156-0.158)	<.0001
HICs	SDI	119	417	0.157 (0.156-0.158)	<.0001
LMICs	SDI	74	351	-0.019 ([-0.033]-[-0.005])	0.0082

Note: Age_1-Age_4 were variables generated in the process of fitting cubic splines, and the knots were 4.5, 8.7, 13.0, 13.6, 40.4 years for current wheezing, 6, 10, 13.5, 13.9, 42.325 years for ever wheezing, 5.175, 9, 13, 32, 67 years for current asthma and 5.5, 9, 13.5, 24.9375, 64.5 years for ever asthma. #The effect of sex was

estimated based on studies where sex-specific prevalence was available.

Appendix 3. Modelling the global prevalence of asthma

Table S4. Models for constructing the prevalence of current wheezing, ever wheezing, current asthma and ever asthma

**asthma**	$1+e^{(-45.3451+0.1988*Age_1-18.4582*Age_2+39.6855*Age_3-22.0276*Age_4-0.0621*female\ proportion+0.0209*Investigation\ year-0.1346*WB_{LMIC}+u_i}$
**Ever asthma**	$\frac{e^{(-109.7344+0.0603*Age_1-6.7893*Age_2+13.077*Age_3-6.5845*Age_4+0.1207*female\ proportion+0.0536*Investigation\ year-0.5438*WB_{LMIC}+u_i)}}{1+e^{(-109.7344+0.0603*Age_1-6.7893*Age_2+13.077*Age_3-6.5845*Age_4+0.1207*female\ proportion+0.0536*Investigation\ year-0.5438*WB_{LMIC}+u_i)}}$

Category	Model for constructing the prevalence
**Current wheezing**	$\frac{e^{(-13.2772+0.01*Age1+0.1141*Age2-0.8725*Age3+5.6997*Age4-0.1024*female\ proportion+0.0056*Investigation\ year-0.0902*WBLMIC+ui)}}{1+e^{(-13.2772+0.01*Age1+0.1141*Age2-0.8725*Age3+5.6997*Age4-0.1024*female\ proportion+0.0056*Investigation\ year-0.0902*WBLMIC+ui)}}$
**Ever wheezing**	$\frac{e^{(-10.8033+0.0738*Age_1-1.6693*Age_2+2.3376*Age_3+13.5855*Age_4-0.1266*female\ proportion+0.0046*Investigation\ year-0.1223*WB_{LMIC}+u_i)}}{1+e^{(-10.8033+0.0738*Age_1-1.6693*Age_2+2.3376*Age_3+13.5855*Age_4-0.1266*female\ proportion+0.0046*Investigation\ year-0.1223*WB_{LMIC}+u_i)}}$
**Current**	$\frac{e^{(-45.3451+0.1988*Age_1-18.4582*Age_2+39.6855*Age_3-22.0276*Age_4-0.0621*female\ proportion+0.0209*Investigation\ year-0.1346*WB_{LMIC}+u_i)}}{}$

Note: Age_1-Age_4 were variables generated in the process of fitting cubic splines, and the knots were 4.5, 8.7, 13.0, 13.6, 40.4 years for current wheezing, 6, 10, 13.5, 13.9, 42.325 years for ever wheezing, 5.175, 9, 13, 32, 67 years for current asthma and 5.5, 9, 13.5, 24.9375, 64.5 years for ever asthma. u_i was the variance of random effect.

Appendix 4. Characteristics of the included studies

Table S5. Characteristics of included studies with prevalence data for sample population

Author	Year Published	Country	Study site	WHO Region	WB Income Region	Investig ation Date	Sampling Strategy	Age range	Group
Hedman J, et al.	1999	Finalnd	Päijät-Häme, Southern Finland	EUR	HIC	May 1996	Random	18-65	Ever diagnosed asthma
Salem, M. B., et al.	2002	Iraq	Basra, Southern Iraq	EMR	LMIC	March-April 2001	Random	0-4	Current wheezing
Obaseki, D. O., et al.	2014	Nigeria	Ile-Ife, Southwest Nigeria	AFR	LMIC	May-Dec 2011	Stratified three-stage sampling	15+	Newly diagnosed asthma
Horak, E., et al.	2014	Austria	Innsbruck, Tyrol	EUR	HIC	2013	Random	7.5-8.5	Current wheezing
Anderson, H. R., et al.	1994	UK	Croydon, London	EUR	HIC	1978-1991	Random	NA	Current wheezing
Crain, E. F., et al.	1994	US	Bronx County, Ney York	AMR	HIC	1991	Random	NA	Ever diagnosed asthma
Kirenga, B. J., et al.	2019	Uganda	National	AFR	LMIC	2014	Random	12+	Ever diagnosed asthma
Seneviratne	2018	Sri Lanka	Colombo Municipal Council	SEAR	LMIC	May 2015 - Dec 2015	Cluster sampling	3-5	Ever wheezing
Yawn	2002	US	Rochester, Minnesota	AMR	HIC	36527	Cluster	5.3-22.1	Ever diagnosed asthma
Bjorksten	1998	Sweden	Linköping	EUR	HIC	1998	Random	13-14; 6-7	Ever wheezing
Bjorksten	1998	Sweden	Stockholm/Uppsala	EUR	HIC	1998	Random	13-14; 6-8	Ever wheezing
Bjorksten	1998	Finland	Kuopio	EUR	HIC	1998	Random	13-14; 6-9	Ever wheezing

Author	Year Published	Country	Study site	WHO Region	WB Income Region	Investigation Date	Sampling Strategy	Age range	Group
Bjorksten	1998	Finland	Lapland	EUR	HIC	1998	Random	13-14; 6-10	Ever wheezing
Bjorksten	1998	Finland	Helsinki	EUR	HIC	1998	Random	13-14; 6-11	Ever wheezing
Bjorksten	1998	Finland	Turku/Pori	EUR	HIC	1998	Random	13-14; 6-12	Ever wheezing
Bjorksten	1998	Estonia	Tallinn	EUR	HIC	1998	Random	13-14; 6-13	Ever wheezing
Bjorksten	1998	Latvia	Riga	EUR	HIC	1998	Random	13-14; 6-14	Ever wheezing
Bjorksten	1998	Latvia	Rural	EUR	HIC	1998	Random	13-14; 6-15	Ever wheezing
Bjorksten	1998	Poland	Krakow	EUR	HIC	1998	Random	13-14; 6-16	Ever wheezing
Bjorksten	1998	Poland	Poznan	EUR	HIC	1998	Random	13-14; 6-17	Ever wheezing
Bjorksten	1998	Albania	Tirané	EUR	LMIC	1998	Random	13-14; 6-18	Ever wheezing
Bjorksten	1998	Romania	Cluj	EUR	LMIC	1998	Random	13-14; 6-19	Ever wheezing
Bjorksten	1998	Russia	Moscow	EUR	LMIC	1998	Random	13-14; 6-20	Ever wheezing
Bjorksten	1998	Georgia	Kutaisi	EUR	LMIC	1998	Random	13-14; 6-21	Ever wheezing
Bjorksten	1998	Georgia	Tbilisi	EUR	LMIC	1998	Random	13-14; 6-22	Ever wheezing
Bjorksten	1998	Uzbekistan	Samarkand	EUR	LMIC	1998	Random	13-14; 6-23	Ever wheezing
Bjorksten	1998	Uzbekistan	Tashkent	EUR	LMIC	1998	Random	13-14; 6-24	Ever wheezing
Bjorksten	1998	Sweden	Stockholm/uppsala	EUR	HIC	1998	Random	13-14; 6-25	Ever wheezing
Bjorksten	1998	Estonia	Tallinn	EUR	HIC	1998	Random	13-14; 6-26	Ever wheezing
Bjorksten	1998	Latvia	Riga	EUR	HIC	1998	Random	13-14; 6-27	Ever wheezing
Bjorksten	1998	Poland	Krakow	EUR	HIC	1998	Random	13-14; 6-28	Ever wheezing
Bjorksten	1998	Poland	Poznan	EUR	HIC	1998	Random	13-14; 6-29	Ever wheezing
Bjorksten	1998	Georgia	Kutaisi	EUR	LMIC	1998	Random	13-14; 6-30	Ever wheezing
Bjorksten	1998	Georgia	Tbilisi	EUR	LMIC	1998	Random	13-14; 6-31	Ever wheezing
Bjorksten	1998	Albania	Tirané	EUR	LMIC	1998	Random	13-14; 6-32	Ever wheezing

Bjorksten	1998	Sweden	Stockholm/uppsala	EUR	HIC	1998	Random	13-14; 6-33	Current wheezing
Bjorksten	1998	Estonia	Tallinn	EUR	HIC	1998	Random	13-14; 6-34	Current wheezing
Bjorksten	1998	Latvia	Rural	EUR	HIC	1998	Random	13-14; 6-35	Current wheezing
Bjorksten	1998	Poland	Krakow	EUR	HIC	1998	Random	13-14; 6-36	Current wheezing
Bjorksten	1998	Poland	Poznan	EUR	HIC	1998	Random	13-14; 6-37	Current wheezing

Author	Year Published	Country	Study site	WHO Region	WB Income Region	Investigation Date	Sampling Strategy	Age range	Group
Bjorksten	1998	Georgia	Kutaisi	EUR	LMIC	1998	Random	13-14; 6-38	Current wheezing
Bjorksten	1998	Georgia	Tbilisi	EUR	LMIC	1998	Random	13-14; 6-39	Current wheezing
Bjorksten	1998	Albania	Tirané	EUR	LMIC	1998	Random	13-14; 6-40	Current wheezing
Bjorksten	1998	Sweden	Stockholm/uppsala	EUR	HIC	1998	Random	13-14; 6-41	Ever diagnosed asthma
Bjorksten	1998	Estonia	Tallinn	EUR	HIC	1998	Random	13-14; 6-42	Ever diagnosed asthma
Bjorksten	1998	Latvia	Rural	EUR	HIC	1998	Random	13-14; 6-43	Ever diagnosed asthma
Bjorksten	1998	Poland	Krakow	EUR	HIC	1998	Random	13-14; 6-44	Ever diagnosed asthma
Bjorksten	1998	Poland	Poznan	EUR	HIC	1998	Random	13-14; 6-45	Ever diagnosed asthma
Bjorksten	1998	Georgia	Kutaisi	EUR	LMIC	1998	Random	13-14; 6-46	Ever diagnosed asthma
Bjorksten	1998	Georgia	Tbilisi	EUR	LMIC	1998	Random	13-14; 6-47	Ever diagnosed asthma
Bjorksten	1998	Albania	Tirané	EUR	LMIC	1998	Random	13-14; 6-48	Ever diagnosed asthma
Bjorksten	1998	Sweden	Stockholm/uppsala	EUR	HIC	1998	Random	13-14; 6-49	Current wheezing
Bjorksten	1998	Sweden	Stockholm/uppsala	EUR	HIC	1998	Random	13-14; 6-50	Current wheezing
Bjorksten	1998	Finland	Kuopio	EUR	HIC	1998	Random	13-14; 6-51	Current wheezing
Bjorksten	1998	Finland	Lapland	EUR	HIC	1998	Random	13-14; 6-52	Current wheezing
Bjorksten	1998	Finland	Helsinki	EUR	HIC	1998	Random	13-14; 6-53	Current wheezing
Bjorksten	1998	Finland	Turku/Pori	EUR	HIC	1998	Random	13-14; 6-54	Current wheezing
Bjorksten	1998	Estonia	Tallinn	EUR	HIC	1998	Random	13-14; 6-55	Current wheezing

Author	Year Published	Country	Study site	WHO Region	WB Income Region	Investigation Date	Sampling Strategy	Age range	Group
Bjorksten	1998	Latvia	Riga	EUR	HIC	1998	Random	13-14; 6-56	Current wheezing
Bjorksten	1998	Latvia	Rural	EUR	HIC	1998	Random	13-14; 6-57	Current wheezing
Bjorksten	1998	Poland	Krakow	EUR	HIC	1998	Random	13-14; 6-58	Current wheezing
Bjorksten	1998	Poland	Poznan	EUR	HIC	1998	Random	13-14; 6-59	Current wheezing
Bjorksten	1998	Albania	Tirané	EUR	LMIC	1998	Random	13-14; 6-60	Current wheezing
Bjorksten	1998	Romania	Cluj	EUR	LMIC	1998	Random	13-14; 6-61	Current wheezing
Bjorksten	1998	Russia	Moscow	EUR	LMIC	1998	Random	13-14; 6-62	Current wheezing
Bjorksten	1998	Georgia	Kutaisi	EUR	LMIC	1998	Random	13-14; 6-63	Current wheezing
Bjorksten	1998	Georgia	Tbilisi	EUR	LMIC	1998	Random	13-14; 6-64	Current wheezing
Bjorksten	1998	Uzbekistan	Samarkand	EUR	LMIC	1998	Random	13-14; 6-65	Current wheezing
Bjorksten	1998	Uzbekistan	Tashkent	EUR	LMIC	1998	Random	13-14; 6-66	Current wheezing
Bjorksten	1998	Sweden	Stockholm/uppsala	EUR	HIC	1998	Random	13-14; 6-67	Ever diagnosed asthma
Bjorksten	1998	Sweden	Stockholm/uppsala	EUR	HIC	1998	Random	13-14; 6-68	Ever diagnosed asthma
Bjorksten	1998	Finland	Kuopio	EUR	HIC	1998	Random	13-14; 6-69	Ever diagnosed asthma
Bjorksten	1998	Finland	Lapland	EUR	HIC	1998	Random	13-14; 6-70	Ever diagnosed asthma
Bjorksten	1998	Finland	Helsinki	EUR	HIC	1998	Random	13-14; 6-71	Ever diagnosed asthma
Bjorksten	1998	Finland	Turku/Pori	EUR	HIC	1998	Random	13-14; 6-72	Ever diagnosed asthma
Bjorksten	1998	Estonia	Tallinn	EUR	HIC	1998	Random	13-14; 6-73	Ever diagnosed asthma
Bjorksten	1998	Latvia	Riga	EUR	HIC	1998	Random	13-14; 6-74	Ever diagnosed asthma
Bjorksten	1998	Latvia	Rural	EUR	HIC	1998	Random	13-14; 6-75	Ever diagnosed asthma
Bjorksten	1998	Poland	Krakow	EUR	HIC	1998	Random	13-14; 6-76	Ever diagnosed asthma
Bjorksten	1998	Poland	Poznan	EUR	HIC	1998	Random	13-14; 6-77	Ever diagnosed asthma
Bjorksten	1998	Albania	Tirané	EUR	LMIC	1998	Random	13-14; 6-78	Ever diagnosed asthma
Bjorksten	1998	Romania	Cluj	EUR	LMIC	1998	Random	13-14; 6-79	Ever diagnosed asthma

Author	Year Published	Country	Study site	WHO Region	WB Income Region	Investigation Date	Sampling Strategy	Age range	Group
Bjorksten	1998	Russia	Moscow	EUR	LMIC	1998	Random	13-14; 6-80	Ever diagnosed asthma
Bjorksten	1998	Georgia	Kutaisi	EUR	LMIC	1998	Random	13-14; 6-81	Ever diagnosed asthma
Bjorksten	1998	Georgia	Tbilisi	EUR	LMIC	1998	Random	13-14; 6-82	Ever diagnosed asthma
Bjorksten	1998	Uzbekistan	Samarkand	EUR	LMIC	1998	Random	13-14; 6-83	Ever diagnosed asthma
Bjorksten	1998	Uzbekistan	Tashkent	EUR	LMIC	1998	Random	13-14; 6-84	Ever diagnosed asthma
Barry	1991	New Zealand	Hastings & Borough of Havelock North	EUR	HIC	Summer Months, 1991	random	12	Ever diagnosed asthma
Barry	1991	UK	North Cardiff & Vale of Glamorgan	EUR	HIC	Summer Months, 1991	random	12	Ever diagnosed asthma
Akkurt	2003	Turkey	Sivas, Anatonia	EUR	LMIC	2003	random	20-107	Current wheezing
**Author**	**Year Published**	**Country**	**Study site**	**WHO Region**	**WB Income Region**	**Investig ation Date**	**Sampling Strategy**	**Age range**	**Group**
Isoaho	1994	Finland	Lieto	EUR	HIC	October 1990 Decemb er 1991	Random	NA	Newly diagnosed asthma
Papageorgiou	1997	Greece	Athens	EUR	HIC	1997	random	20-44	Ever wheezing
Brozek	2015	Poland	Chorzow	EUR	HIC	1993	Clusterrandom	7-10	Ever diagnosed asthma
Bechtold	2013	Italy	Northeast Italy	EUR	HIC	2000-2004	Random	13	Ever diagnosed asthma
Nriagu	1999	South Africa	Merebank, Durban	AFR	LMIC	1999	Random	18+	Current wheezing
Anderson	2004	UK	British Ishes	EUR	HIC	1995	Random	12-14	Current wheezing
Mallol	2010	Brazil	Latin America	AMR	LMIC	2005-2007	Random	13 -15 months	Current wheezing
Mallol	2010	Spain	Europe	EUR	HIC	2005-2007	Random	13 -15 months	Current wheezing

Author	Year Published	Country	Study site	WHO Region	WB Income Region	Investigation Date	Sampling Strategy	Age range	Group
Johnson	2005	US	Detroit	AMR	HIC	August 2001 - January 2002	Random	<18 - 70+	Ever diagnosed asthma
Solis Soto	2014	Bolivia	Oropeza	AMR	LMIC	July 2011 - December 2011	One-staged cluster	9-15	Ever wheezing
Mattei	2007	Italy	L'Aquila	EUR	HIC	January-March 2004	Random	13-14	Ever diagnosed asthma
Lee	2001	Korea, Rep	Seoul and Provincial center, Korea	WPR	HIC	September-November 1995	Stratified random	6-12	Ever wheezing
Lima	2012	Brazil	National	AMR	LMIC	July 2008 - May 2009	NA	NA	Current wheezing
Hansen	2000	Denmark	Copenhagen	EUR	HIC	1976-1978	Random	20-35	Ever diagnosed asthma
Fukutomi	2011	Japan	Fujieda	WPR	HIC	1985-2006	Random	15+	Ever wheezing
Al Ghobain	2012	Saudi Arabia	Riyadh	EMR	HIC	2010	Two-stage cluster	16-18	Ever wheezing
Kausel	2013	Chile	Region de los Rios and Valdivia	AMR	HIC	2009	Random	10-16	Ever diagnosed asthma
von Mutius	1992	Germany	Leipzig	EUR	HIC	1989-1991	Random	9-11	Ever diagnosed asthma

Alves	1994	Portugal	Oporto	EUR	HIC	1992	Random	20-44	Current wheezing
Liao	2005	Taiwan	Changhwa, Central Taiwan	WPR	HIC	September 2002 - December 2002	Stratified Random	6-8	Ever wheezing
Ehrlich	1995	South Africa	Cape Town	AFR	LMIC	1993	Cluster sampling	7-8	Current wheezing
Arnedo-Pana	2011	Spain	Bilbao	EUR	HIC	2002-2003	Random	6-7	Ever diagnosed asthma
Arnedo-Pana	2011	Spain	Asturias	EUR	HIC	2002-2003	Random	6-7	Ever diagnosed asthma
Arnedo-Pana	2011	Spain	San Sebastian	EUR	HIC	2002-2003	Random	6-7	Ever diagnosed asthma
Arnedo-Pana	2011	Spain	La Coruna	EUR	HIC	2002-2003	Random	6-7	Ever diagnosed asthma
Arnedo-Pana	2011	Spain	Cartagena	EUR	HIC	2002-2003	Random	6-7	Ever diagnosed asthma
Arnedo-Pana	2011	Spain	Barcelona	EUR	HIC	2002-2003	Random	6-7	Ever diagnosed asthma
Arnedo-Pana	2011	Spain	Valencia	EUR	HIC	2002-2003	Random	6-7	Ever diagnosed asthma

Author	Year Published	Country	Study site	WHO Region	WB Income Region	Investigation Date	Sampling Strategy	Age range	Group
Arnedo-Pana	2011	Spain	Madrid	EUR	HIC	2002-2003	Random	6-7	Ever diagnosed asthma
Arnedo-Pana	2011	Spain	Bilbao	EUR	HIC	2002-2003	Random	13-14	Ever diagnosed asthma
Arnedo-Pana	2011	Spain	Asturias	EUR	HIC	2002-2003	Random	13-14	Ever diagnosed asthma
Arnedo-Pana	2011	Spain	San Sebastian	EUR	HIC	2002-2003	Random	13-14	Ever diagnosed asthma

Author	Year Published	Country	Study site	WHO Region	WB Income Region	Investigation Date	Sampling Strategy	Age range	Group
Arnedo-Pana	2011	Spain	La Coruna	EUR	HIC	2002-2003	Random	13-14	Ever diagnosed asthma
Arnedo-Pana	2011	Spain	Cartagena	EUR	HIC	2002-2003	Random	13-14	Ever diagnosed asthma
Arnedo-Pana	2011	Spain	Barcelona	EUR	HIC	2002-2003	Random	13-14	Ever diagnosed asthma
Arnedo-Pana	2011	Spain	Valencia	EUR	HIC	2002-2003	Random	13-14	Ever diagnosed asthma
Arnedo-Pana	2011	Spain	Madrid	EUR	HIC	2002-2003	Random	13-14	Ever diagnosed asthma
Arnedo-Pana	2011	Spain	Valladolid	EUR	HIC	2002-2003	Random	13-14	Ever diagnosed asthma
Nugmanova	2018	Ukraine	Kiev	EUR	LMIC	2013-2015	Two-step cluster random	18+	Ever diagnosed asthma
Nugmanova	2018	Kazakhstan	Almaty	EUR	LMIC	2013-2015	Two-step cluster random	18+	Ever diagnosed asthma
Nugmanova	2018	Azerbaijan	Baku	EUR	LMIC	2013-2015	Two-step cluster random	18+	Ever diagnosed asthma
Nugmanova	2018	Ukraine	Kiev	EUR	LMIC	2013-2015	Two-step cluster random	18+	Ever wheezing
Nugmanova	2018	Kazakhstan	Almaty	EUR	LMIC	2013-2015	Two-step cluster random	18+	Ever wheezing
Nugmanova	2018	Azerbaijan	Baku	EUR	LMIC	2013-2015	Two-step cluster random	18+	Ever wheezing
Cazzola	2011	Italy	Italy	EUR	HIC	40148	Random	14+	Ever diagnosed asthma

Heinrich	2002	Germany	Sachsen-Anhalt	EUR	HIC	1992-1993	Random	5-14	Ever diagnosed asthma
James	2010	Australia	Busselton	WPR	HIC	1990	Random	18-54	Ever diagnosed asthma
Ronchetti	2001	Italy	Rome	EUR	HIC	1974-1998	Random	6-14	Ever diagnosed asthma
Wang	2004	Singapore	Singapore	WPR	HIC	1994-2001	Cluster sampling	6-7	Ever wheezing
Bemanin	2015	Afghanistan	Kabul	EMR	LMIC	2010-2011	Random	NA	Ever diagnosed asthma
Robertson	1991	Australia	Melbourne	WPR	HIC	1990	Two stage random	7-15	Current wheezing
Roos	2018	Netherlands	Rotterdam	EUR	HIC	2018	Random	NA	Ever diagnosed asthma
Toelle	2004	Australia	Belmont	WPR	HIC	1992-2002	Random	8-11	Ever diagnosed asthma
Brunner	2005	US	Minnesota	AMR	HIC	2001	Stratified random	13-16	Current wheezing
Hasnain	2009	Pakistan	Karachi	EMR	LMIC	2007	Random	3-16	Ever diagnosed asthma
Reis	2015	Brazil	Salvador	AMR	LMIC	2006	Random	< 4	Current wheezing
Nguyen	2015	US	Orange County	AMR	HIC	June 2011 and April 2012	Random	3-12	Ever diagnosed asthma
Huure	2004	Finland	Southern Finland	EUR	HIC	1983-1999	Random	16-32	Ever diagnosed asthma
Ozdemir	2000	Turkey	Eskisehir, Turkey	EUR	LMIC	1997-1998	Two-step sampling	16-30	Current wheezing
Liao	2009	Taiwan	Changhwa, Central Taiwan	WPR	LMIC	2002-2007	Cluster random	6-8	Ever wheezing

Author	Year Published	Country	Study site	WHO Region	WB Income Region	Investigation Date	Sampling Strategy	Age range	Group

Strachan	1994	UK	Great Britain	EUR	HIC	March and April, 1992	Two stage stratified random	5-17	Current wheezing
Weiland	1999	Germany	East and West of Germany	EUR	HIC	1995-1996	Random	5-11	Current wheezing
Ronmark	2002	Sweden	Northern Sweden	EUR	HIC	1996-1998	Random	7-8	Ever wheezing
Saraclar	1997	Turkey	Ankara	EUR	LMIC	1994	Random	18+	Ever wheezing
Nishima	2009	Japan	Japan	WPR	HIC	NA	Random	6-12	Ever diagnosed asthma
Lee	2003	US	Boston, Chinatown	AMR	HIC	2003	Random	5-10	Ever diagnosed asthma
Vanfleteren	2012	Netherlands	Maastricht	EUR	HIC	Oct 2007-Mar 2009	Random	40+	Ever diagnosed asthma
Renwick	1996	UK	Leeds	EUR	HIC	Jan 1992 - Feb 1994	Random	NA	Current wheezing
Matsumoto	2015	Japan	Hisayama	SEAR	HIC	2008	Random	40+	Ever diagnosed asthma
Carlsson	2013	Sweden	Stockholm	EUR	HIC	40878	Random	0-85+	Ever diagnosed asthma
Maia	2004	Brazil	Montes Claros, Brazil	AMR	LMIC	2004	Random	13-14	Current wheezing
Kiboneka	2016	Botswana	Gaborone, Botswana	AFR	LMIC	October 2014 to November 2015	Random	6-7; 13-14	Ever wheezing
Hu	1997	US	Los Angeles and San Diego	AMR	HIC	1993	Random	20-22	Ever diagnosed asthma
Pakhale	2008	India	Maharashtra, West India	SEAR	LMIC	November - December, 2004	Random	13-14	Ever diagnosed asthma

Author	Year Published	Country	Study site	WHO Region	WB Income Region	Investig ation Date	Sampling Strategy	Age range	Group
Lawson	2017	Canada	Saskatchewan	AMR	HIC	2013	Random	5-14	Ever diagnosed asthma
Rizwan	2004	UK	Bootle, Merseyside	EUR	HIC	1991	Random	5-11	Ever diagnosed asthma
Freitas	2012	Brazil	Brazilian Amazon islands, Para	AMR	LMIC	2007-2009	Convenienc e	5-8	Ever wheezing
Findley	2003	US	East, Harlem	AMR	HIC	NA	Random	NA	Ever diagnosed asthma
Simon	2003	US	Los Angeles	AMR	HIC	Septemb er 1999-April 2000	Random	< 18	Ever diagnosed asthma
Hoffmann	2007	Germany	East and West, Germany	EUR	HIC	2003	Two-staged	18+	Ever diagnosed asthma
Claudio	2006	US	New York City	AMR	HIC	2002-2003	Stratified Random	4-13	Newly diagnosed asthma
Silva	2013	Brazil	Salvador, Northeast Brazil	AMR	LMIC	AugustDecemb er 2010	Random	6-12	Current wheezing
Bryant-Stephens	2012	US	West and North Philadelphia	AMR	HIC	2006-2008	Random	5-14	Ever diagnosed asthma
Goh	1996	Singapore	Singapore	WPR	HIC	Februar y - Novemb er, 1994	Random	6-7	Ever wheezing
Carillo	2018	US	Hidalgo, County Texas	AMR	HIC	October 2014-May 2016	Random	2-18	Ever diagnosed asthma
Mallol	2010	Argentina	Argentina	AMR	HIC	2010	Random	13-14	Current wheezing
Mallol	2010	Bolivia	Bolivia	AMR	LMIC	2010	Random	13-14	Current wheezing

Author	Year Published	Country	Study site	WHO Region	WB Income Region	Investigation Date	Sampling Strategy	Age range	Group
Mallol	2010	Brazil	Brazil	AMR	LMIC	2010	Random	13-14	Current wheezing
Mallol	2010	Chile	Chile	AMR	HIC	2010	Random	13-14	Current wheezing
Mallol	2010	Colombia	Colombia	AMR	LMIC	2010	Random	13-14	Current wheezing
Mallol	2010	Costa Rica	Costa Rica	AMR	LMIC	2010	Random	13-14	Current wheezing
Mallol	2010	Cuba	Cuba	AMR	LMIC	2010	Random	13-14	Current wheezing
Mallol	2010	Ecuador	Ecuador	AMR	LMIC	2010	Random	13-14	Current wheezing
Mallol	2010	El Savador	El Savador	AMR	LMIC	2010	Random	13-14	Current wheezing
Mallol	2010	Honduras	Honduras	AMR	LMIC	2010	Random	13-14	Current wheezing
Mallol	2010	Mexico	Mexico	AMR	LMIC	2010	Random	13-14	Current wheezing
Mallol	2010	Nicaragua	Nicaragua	AMR	LMIC	2010	Random	13-14	Current wheezing
Mallol	2010	Panama	Panama	AMR	LMIC	2010	Random	13-14	Current wheezing
Mallol	2010	Paraguay	Paraguay	AMR	LMIC	2010	Random	13-14	Current wheezing
Mallol	2010	Peru	Peru	AMR	LMIC	2010	Random	13-14	Current wheezing
Mallol	2010	Uruguay	Uruguay	AMR	HIC	2010	Random	13-14	Current wheezing
Mallol	2010	Venezuela	Venezuela	AMR	LMIC	2010	Random	13-14	Current wheezing
Mallol	2010	Argentina	Argentina	AMR	HIC	2010	Random	13-14	Ever diagnosed asthma
Mallol	2010	Bolivia	Bolivia	AMR	LMIC	2010	Random	13-14	Ever diagnosed asthma
Mallol	2010	Brazil	Brazil	AMR	LMIC	2010	Random	13-14	Ever diagnosed asthma
Mallol	2010	Chile	Chile	AMR	HIC	2010	Random	13-14	Ever diagnosed asthma
Mallol	2010	Colombia	Colombia	AMR	LMIC	2010	Random	13-14	Ever diagnosed asthma
Mallol	2010	Costa Rica	Costa Rica	AMR	LMIC	2010	Random	13-14	Ever diagnosed asthma
Mallol	2010	Cuba	Cuba	AMR	LMIC	2010	Random	13-14	Ever diagnosed asthma
Mallol	2010	Ecuador	Ecuador	AMR	LMIC	2010	Random	13-14	Ever diagnosed asthma
Mallol	2010	El Savador	El Savador	AMR	LMIC	2010	Random	13-14	Ever diagnosed asthma

Mallol	2010	Honduras	Honduras	AMR	LMIC	2010	Random	13-14	Ever diagnosed asthma
Mallol	2010	Mexico	Mexico	AMR	LMIC	2010	Random	13-14	Ever diagnosed asthma
Mallol	2010	Nicaragua	Nicaragua	AMR	LMIC	2010	Random	13-14	Ever diagnosed asthma
Mallol	2010	Panama	Panama	AMR	LMIC	2010	Random	13-14	Ever diagnosed asthma
Mallol	2010	Paraguay	Paraguay	AMR	LMIC	2010	Random	13-14	Ever diagnosed asthma
Mallol	2010	Peru	Peru	AMR	LMIC	2010	Random	13-14	Ever diagnosed asthma
Mallol	2010	Uruguay	Uruguay	AMR	HIC	2010	Random	13-14	Ever diagnosed asthma

Author	Year Published	Country	Study site	WHO Region	WB Income Region	Investigation Date	Sampling Strategy	Age range	Group
Mallol	2010	Venezuela	Venezuela	AMR	LMIC	2010	Random	13-14	Ever diagnosed asthma
Romano-Zelekha	2007	Israel	Isreal	EUR	HIC	1997	Random	13-14	Ever diagnosed asthma
El Ftouh	2009	Algeria	Algeria	AFR	LMIC	January 2008-May 2008	Random stratified	NA	Ever diagnosed asthma
El Ftouh	2009	Morocco	Morocco	EMR	LMIC	January 2008-May 2008	Random stratified	NA	Ever diagnosed asthma
El Ftouh	2009	Tunisia	Tunisia	EMR	LMIC	January 2008-May 2008	Random stratified	NA	Ever diagnosed asthma
Stojanovic-Rist	2006	Serbia	Belgrade, Serbia	EUR	LMIC	1996-2001	Random	19-23	Ever diagnosed asthma
Shamssain	1999	UK	North East, England	EUR	HIC	1999	Random	6-7	Ever wheezing

Kim	2013	Korea, Rep	Korea	WPR	HIC	1998-2008	Multistaged Stratified Probability	20-59	Ever diagnosed asthma
Romanmtowski	2015	Poland	Tricity	EUR	HIC	April 2011 - December 2011	Random	1-18	Ever diagnosed asthma
Brugge	2008	US	Dorchester, Massachusetts	AMR	HIC	2005-2006	Convenience	18+	Current wheezing
Shimwela	2014	Tanzania	Ilala and Bagamoyo	AFR	LMIC	2007	Random	0+	Ever diagnosed asthma
Senthilselvan	2016	Canada	10 Canadian provinces, the Northwest Territories and the Yukon	AMR	HIC	2002-2010	Two-staged statified	0-11	Ever diagnosed asthma

Author	Year Published	Country	Study site	WHO Region	WB Income Region	Investigation Date	Sampling Strategy	Age range	Group
Ziyab	2017	Kuwait	Kuwait	EMR	HIC	January -May 2015	Random	18-26	Ever wheezing
Kainu	2013	Finland	Helsinki	EUR	HIC	1996-2006	Random	20-69	Ever diagnosed asthma
Gershon	2010	Canada	Ontario	AMR	HIC	1991-2005	Random	0+	Ever diagnosed asthma
Gershon	2010	Canada	Ontario	AMR	HIC	1991-2005	Random	0+	Ever diagnosed asthma
Singh	2016	India	Rajasthan, Punjab, Maharashtra, Kerala and Karnatak	SEAR	LMIC	2001-2003	Random	6-14	Ever diagnosed asthma

Peroni	2009	Italy	Verona, Northern Italy	EUR	HIC	2009	Random	3-5	Current wheezing
Kaur	1998	UK	England, Wales, and Scotland	EUR	HIC	1995	Statified Clustered	12-14	Ever wheezing
Kuehni	2000	UK	Leicester	EUR	HIC	1985-1998	Age-stratified random	8-13	Current wheezing
Delmas	2009	France	France	EUR	HIC	October 2002-October 2003	Random	11-14	Current wheezing
Werneck	1999	Brazil	Itabira or Santa Maria	AMR	LMIC	June - October 1994	Random	7-8	Ever diagnosed asthma
Norzila	2000	Malaysia	Kuala Lumpur	WPR	LMIC	2000	Random	7-12	Ever wheezing
Ngui	2011	Malaysia	Orang Asli	WPR	LMIC	November 2007 - July 2009	Random	0+	Current wheezing
Felix	2015	US	Puerto Rico	AMR	HIC	2008-2010	Random	18+	Ever diagnosed asthma

**Author**	**Year Published**	**Country**	**Study site**	**WHO Region**	**WB Income Region**	**Investigation Date**	**Sampling Strategy**	**Age range**	**Group**
Anthracopoulos	2007	Greece	Patras	EUR	HIC	1991-2003	Random	8-10	Current wheezing
Lamnisos	2013	Cyprus	Greek-Cypriot & Turkish-Cypriot	EUR	HIC	2007-2009	Random	7-8 & 13-14	Current wheezing
Bishwajit	2017	Bangladesh	South Asia	SEAR	LMIC	January 2002 December 2002	Random	18+	Ever diagnosed asthma

Author	Year Published	Country	Study site	WHO Region	WB Income Region	Investigation Date	Sampling Strategy	Age range	Group
Bishwajit	2017	India	South Asia	SEAR	LMIC	January 2002 December 2002	Random	18+	Ever diagnosed asthma
Bishwajit	2017	Nepal	South Asia	SEAR	LMIC	January 2002 December 2002	Random	18+	Ever diagnosed asthma
Bishwajit	2017	Pakistan	South Asia	EMR	LMIC	January 2002 December 2002	Random	18+	Ever diagnosed asthma
Bishwajit	2017	Sri Lanka	South Asia	SEAR	LMIC	January 2002 December 2002	Random	18+	Ever diagnosed asthma
Austin	1994	UK	Scotland	EUR	HIC	1992	Random	12-13	Ever diagnosed asthma
Moradi-Lakeh	2015	Saudi Arabia	Saudi	EMR	HIC	2015	Multistaged	15+	Ever diagnosed asthma
Fedortsiv	2012	Ukraine	Ternopil	EUR	LMIC	2010	Cluster	6-14	Ever diagnosed asthma
Trakultivakorn	2007	Thailand	Bangkok and Chiang Mai	SEAR	LMIC	2001	Random	6-7	Ever diagnosed asthma
Gupta	2001	India	Chandigarh, North India	SEAR	LMIC	July 1997- June 1999	Random	9-20	Ever diagnosed asthma

Musafiri	2011	Rwanda	Kigali and Huye, Southern Rwanda	AFR	LMIC	February 2008 and August 2009	Random	15-80	Current wheezing
Kim	2014	Sweden	Vastra Gotaland County	EUR	HIC	2000-2008	Random	15	Current wheezing
Greenlee	2008	US	Marshfield	AMR	HIC	2008	Random	5-17	Ever wheezing
Ma	2017	China	China	WPR	LMIC	2009 and 2011	Random	18+	Ever diagnosed asthma
Middleton	2014	Cyprus	Cyprus	EUR	HIC	2006-2007	Random	15-17	Newly diagnosed asthma
Shakurnia	2010	Iran	Ahvaz city, south-west	EMR	LMIC	2007	Random	6-7	Ever wheezing
Jarvis	1994	UK	Cambridge, Ipswich and Norwich	EUR	HIC	October 1990-June 1991	Random	20-44	Current wheezing
Glushkova	2008	Russia	Vasiljevki, Central St. Petersburg	EUR	LMIC	2003	Random	0-18	Ever diagnosed asthma
Nystad	1997	Norway	Oslo	EUR	HIC	1981 & 1994	Random	6-16	Ever diagnosed asthma
Rose	2006	US	Puerto Ricans and in the Northeast census region	AMR	HIC	1998-2000	Complex Multistaged	18+	Ever diagnosed asthma
Jindal	2000	India	Chandigarh	SEAR	LMIC	July 1995-June 1997	Random	18+	Ever diagnosed asthma

Author	Year Published	Country	Study site	WHO Region	WB Income Region	Investigation Date	Sampling Strategy	Age range	Group
Peat	1994	Australia	New South Wales	WPR	HIC	June 1982 - 1992	Random	8-10	Ever diagnosed asthma
Gao	2008	Canada	Northwest Territories	AMR	HIC	1994-1995	Random	0-11	Ever diagnosed asthma
Jerning	2013	Sweden	Stockholm, Gothenburg, Uppsala and Umea	EUR	HIC	2008	Random	16-75	Newly diagnosed asthma
Wehrmeister	2010	Brazil	South and Northeast regions	AMR	LMIC	2003	Random	<10	Ever diagnosed asthma
Al Ghobain, M. O., et al.	2012	Saudi Arabia	Riyadh, Saudi Arabia.	EMR	HIC	2009-2010	Random	16-18	Ever wheezing
Barraza Villarreal, A., et al.	2003	Mexico	Juarez, Chihuahua, Mexico,	AMR	LMIC	2003	Random	6-14	Ever diagnosed asthma
Tan, T. N., et al.	2006	Singapore	Singapore	WPR	HIC	2000	Random	4-6	Ever wheezing
Sole, D., et al.	2015	Brazil	Brazil	AMR	LMIC	2012	Random	13-14	Ever wheezing
Kabesch, M., et al.	1999	Germany	Munich, Germany	EUR	HIC	1989-1990	Random	9-11	Current wheezing
Broms, K., et al.	2012	Sweden	Sweden	EUR	HIC	2002-2007	Random	1-6	Ever wheezing
Chan-Yeung, M., et al.	2002	China	Beijing, China	WPR	LMIC	1996-1997	Random	=/>15	Ever wheezing
Mvula, M., et al.	2005	US	New Orleans, US	AMR	HIC	2000	Random	NA	Ever wheezing
Joseph, C. L., et al.	1996	US	Detroit, US	AMR	HIC	1993	Random	NA	Ever diagnosed asthma

Author	Year Published	Country	Study site	WHO Region	WB Income Region	Investigation Date	Sampling Strategy	Age range	Group
Pekkanen, J., et al.	1997	Finland	Helsinki area; Turku and Pori County; Kuo-pio County; and the southern part of Laplan	EUR	HIC	1994-1995	Random	13-14	Ever wheezing
Habbick, B. F., et al.	1999	Canada	Hamilton & Saskatoon	AMR	HIC	1994-1995	Random	6-7 & 13-14	Ever diagnosed asthma
Shohat, T., et al.	2000	Israel	Israel	EUR	HIC	1997	Random	13-14	Newly diagnosed asthma
Leung, R., et al.	1994	Hong Kong	Hong Kong	WPR	HIC	1992	Random	7, 12 & 15	Ever wheezing
Yeatts, K. B. and C. M. Shy	2001	US	North Carolina	AMR	HIC	1996	Random	13-15	Ever wheezing
Arif, A. A., et al.	2005	US	West Texas	AMR	HIC	2001	Random	=/>65	Ever diagnosed asthma
Meren, M., et al.	2005	Estonia	Tallinn, Narva and Saaremaa	EUR	HIC	1995-2000	Random	15-64	Current wheezing
Larsson, L.	1995	Sweden	Jamtland and Gastrikland.	EUR	HIC	1990-1993	Random	13-24	Ever wheezing
Rona, R. J., et al.	1995	UK	UK	EUR	HIC	1982 & 1992	Random	5-11	Current wheezing
Mellinger-Birdsong, A. K., et al.	2003	US	Georgia	AMR	HIC	2000	Random	0-17	Ever diagnosed asthma
Dow, L., et al.	2001	UK	South west England	EUR	HIC	2001	Random	=/>65	Ever diagnosed asthma
Unlu, M., et al.	2002	Turkey	Afyon	EUR	LMIC	2001	Random	13-18	Current wheezing
Al Ghobain, M. O., et al.	2018	Saudi Arabia	Riyadh, Saudi Arabia.	EMR	HIC	2016	Random	20-44	Current wheezing
McCann, D., et al.	2002	UK	South of England	EUR	HIC	2002	Random	7-9	Ever diagnosed asthma

Author	Year Published	Country	Study site	WHO Region	WB Income Region	Investigation Date	Sampling Strategy	Age range	Group
Pallasaho, P., et al.	2002	Sweden	Helsinki, Stockholm, and Tallinn	EUR	HIC	1996	Random	20-64	Current wheezing
Khan, A. A., et al.	2014	Pakistan	Karachi	EMR	LMIC	2012-2013	Random	3-17	Ever diagnosed asthma
Aberle, N., et al.	2018	Croatia	Brod-Posavina County	EUR	LMIC	2007-2008	Random	10-11	Ever wheezing
Valery, P. C., et al.	2001	Australia	Torres Strait and Northern Peninsula Area	WPR	HIC	1999	Random	0-17	Ever wheezing
Kurukulaaratchy, R. J., et al.	2002	UK	Isle of Wight	EUR	HIC	1989-1999	Random	10	Current wheezing
Leung, R. and P. Ho	1994	Malaysia	Kota Kinabalu	WPR	HIC	1992	Random	11-20	Ever wheezing
Letamo, G., et al.	2017	Botswana	Botswana	AFR	LMIC	2013	Random	10-64	Ever diagnosed asthma
Yoshihara, S., et al.	2016	Mongolia	Ulaanbaatar	WPR	LMIC	2009	Random	6-7	Ever wheezing
Levesque, B., et al.	2004	Canada	Quebec	AMR	HIC	1999	Random	9, 13 & 16	Ever diagnosed asthma
Schuh, C., et al.	2015	Brazil	Porto Alegre	AMR	LMIC	2013	Random	10-18	Newly diagnosed asthma
Cakir, E., et al.	2010	Turkey	Istanbul's Kartal County	EUR	LMIC	2010	Random	</=16 & >17	Ever wheezing
Zivkovic, Z., et al.	2010	Serbia	Belgrade, Nis, Novi Sad & Sombor	EUR	LMIC	2009	Random	6-7 &13-14	Ever diagnosed asthma
Zivkovic, Z., et al.	2010	Montenegro	Podgorica	EUR	LMIC	2009	Random	6-7 &13-14	Ever diagnosed asthma
Hessel, P. A., et al.	2001	Canada	Alberta (Red deer & Medicine Hat)	AMR	HIC	2000	Random	5-19	Ever diagnosed asthma

Author	Year Published	Country	Study site	WHO Region	WB Income Region	Investigation Date	Sampling Strategy	Age range	Group
Stout, J. W., et al.	2001	US	Tacoma, Washington and Alaska	AMR	HIC	1997-1998	Random	11-16	Ever diagnosed asthma
Chhabra, S. K., et al.	1998	India	Delhi	SEAR	LMIC	1997	Random	4-17	Current wheezing
Jain, A., et al.	2010	India	Manipal	SEAR	LMIC	2009	Random	6-15	Current wheezing
Yemaneberhan, H., et al.	1997	Ethiopia	Jimma	AFR	LMIC	1996	Random	0-70=/>	Current wheezing
Pite, H., et al.	2014	Portugal	Mainland Portugal	EUR	HIC	2008	Random	=/>65	Ever diagnosed asthma
Ponsonby, A. L., et al.	2008	Australia	Australian Capital Territory	WPR	HIC	1999-2005	Random	4-6	Ever diagnosed asthma
Gonzalez-Garcia, M., et al.	2015	Colombia	Barranquilla, Bucaramanga, Cali, Medellin and Bogota	AMR	LMIC	2015	Random	40-93	Ever wheezing
Teeratakulpisarn, J., et al.	2000	Thailand	Khon Kaen	SEAR	LMIC	2003	Random	6-7 & 13-14	Ever wheezing
Leung, R., et al.	1997	Hong Kong	Hong Kong	WPR	HIC	1994-1995	Random	13-14	Ever diagnosed asthma
Ding, Y. P., et al.	2012	China	Hainan	WPR	LMIC	2012	Random	2-90	Ever diagnosed asthma
Dennis, R. J., et al.	2012	Colombia	Barranquilla, Bogotá, Bucaramanga, Cali, Medellin, and San Andrés Island	AMR	LMIC	2009-2010	Random	1-59	Ever wheezing
Zhu, W. J., et al.	2015	China	Beijing	WPR	LMIC	2010-2011	Random	1-14	Ever diagnosed asthma

Faniran, A. O., et al.	1999	Australia	Sydney	WPR	HIC	1999	Random	8-11	Ever diagnosed asthma
Faniran, A. O., et al.	1999	Nigeria	Ojo	AFR	LMIC	1999	Random	8-11	Ever diagnosed asthma
Nordlund, B., et al.	2014	Sweden	Stockholm	EUR	HIC	1994-2008	Random	0-12	Ever diagnosed asthma
Nelson, K. A., et al.	2009	US	St. Louis, Missouri	AMR	HIC	1992 & 2004	Random	10-12	Current wheezing
Sole, D., et al.	2015	Brazil	Belem	AMR	LMIC	2011-2012	Random	13-14	Ever wheezing
Sole, D., et al.	2015	Brazil	Recife	AMR	LMIC	2011-2012	Random	13-14	Ever wheezing
Sole, D., et al.	2015	Brazil	Macelo	AMR	LMIC	2011-2012	Random	13-14	Ever wheezing
**Author**	**Year Published**	**Country**	**Study site**	**WHO Region**	**WB Income Region**	**Investigation Date**	**Sampling Strategy**	**Age range**	**Group**
Sole, D., et al.	2015	Brazil	Aracaju	AMR	LMIC	2011-2012	Random	13-14	Ever wheezing
Sole, D., et al.	2015	Brazil	Belo Horizonte	AMR	LMIC	2011-2012	Random	13-14	Ever diagnosed asthma
Sole, D., et al.	2015	Brazil	Sao Paulo	AMR	LMIC	2011-2012	Random	13-14	Ever diagnosed asthma
Sole, D., et al.	2015	Brazil	Curitiba	AMR	LMIC	2011-2012	Random	13-14	Ever diagnosed asthma
Zollner, I. K., et al.	2005	Germany	Baden-Wuerttemberg: Mannheim, Kehl and Aulendorf/Bad Waldsee	EUR	HIC	1992-2001	Random	9-11	Ever diagnosed asthma
Wanlapakorn, N., et al.	2014	Thailand	Bangkok	SEAR	LMIC	2012	Random	6-12	Ever wheezing

Demir, A. U., et al.	2010	Turkey	Ankara	EUR	LMIC	1992-2007	Random	7-12	Ever diagnosed asthma
Wang, H. Y., et al.	2008	China	Guangzhou, Beijing and Hong Kong,	WPR	LMIC	2001-2003	Random	13-14	Ever wheezing
Wang, H. Y., et al.	2008	Canada	Vancouver	AMR	HIC	2001-2003	Random	13-14	Ever wheezing
Pallasaho, P., et al.	1999	Finland	Helsinki	EUR	HIC	1996	Random	20-69	Current wheezing
Tarraf, H., et al.	2018	Egypt	Egypt	EMR	LMIC	2014-2016	Random	18 and above	Ever diagnosed asthma
Tarraf, H., et al.	2018	Gulf countries	Kuwait, Saudi Arabia, and the United Arab Emirates	EMR	LMIC	2014-2016	Random	18 and above	Ever diagnosed asthma
Tarraf, H., et al.	2018	Turkey	Turkey	EUR	LMIC	2014-2016	Random	18 and above	Ever diagnosed asthma
Al-Thamiri, D., et al.	2005	Iraq	Baghdad	EMR	LMIC	2000-2002	Random	6-15	Ever wheezing

**Author**	**Year Published**	**Country**	**Study site**	**WHO Region**	**WB Income Region**	**Investigation Date**	**Sampling Strategy**	**Age range**	**Group**
Roncada, C., et al.	2016	Brazil	Porto Alegre	AMR	LMIC	2013	Random	8-16	Current wheezing
Peat, J. K., et al.	1992	Australia	Busselton	WPR	HIC	1981 & 1990	Random	18-55	Ever diagnosed asthma
Ekici, A., et al. (2012)	2012	Turkey	Kirikkale	EUR	LMIC	2004	Random	NA	Ever diagnosed asthma
Palvo, F., et al.	2008	Brazil	Sa˜ o Jose´ do Rio Preto, Sa˜ o Paulo	AMR	LMIC	2003-2004	Random	6-7	Ever diagnosed asthma
Tang, S. P., et al.	2017	China	Fuzhou	WPR	LMIC	2010-2011	Random	0-14	Ever diagnosed asthma

Author	Year Published	Country	Study site	WHO Region	WB Income Region	Investigation Date	Sampling Strategy	Age range	Group
Musharrafieh, U., et al.	2009	Lebanon	Beirut, Bekaa, South & North	EMR	LMIC	2005	Random	13-14	Ever wheezing
Dales, R. E., et al.	1994	Canada	British Columbia, Saskatchewan, Ontario, Quebec, and Maritimes	AMR	HIC	1988	Random	5-8	Ever diagnosed asthma
Bartlett, E., et al.	2013	Bangladesh	Abhoynagar, Mirsarai & Kamalapur	SEAR	LMIC	2009	Random	25-65>	Ever diagnosed asthma
Perez-Perdomo, R., et al.	2003	US	Puerto Rico	AMR	HIC	2000	Random	18 and above	Ever diagnosed asthma
Nafti, S., et al.	2009	Algeria	Algeria	AFR	LMIC	2008	Random	16 and above	Newly diagnosed asthma
Nafti, S., et al.	2009	Morocco	Morocco	EMR	LMIC	2008	Random	16 and above	Newly diagnosed asthma
Nafti, S., et al.	2009	Tunisia	Tunisia	EMR	LMIC	2008	Random	16 and above	Newly diagnosed asthma
Manfreda, J., et al.	2001	Canada	Vancouver, Winnipeg, Hamilton, Montreal, Halifax and Prince Edward Island	AMR	HIC	1993-1994	Random	20–44	Current wheezing
Linehan, M. F., et al.	2009	UK	Manchester	EUR	HIC	2002-2004	Random	6-11	Current wheezing
Song, N., et al.	2014	China	Shijiazhuang in Hebei province	WPR	LMIC	2011	Random	6-18	Ever wheezing

Author	Year Published	Country	Study site	WHO Region	WB Income Region	Investigation Date	Sampling Strategy	Age range	Group
Sole, D., et al.	2014	Brazil	Brazil	AMR	LMIC	NA	Random	6-7 & 13-14	Current wheezing
Ronmark, E., et al.	2001	Sweden	Kiruna, Luleå, and Piteå in northern Sweden	EUR	HIC	1996 & 1997	Random	7-8	Ever diagnosed asthma
Brozek, G., et al.	2016	Belarus	Grodno	EUR	LMIC	NA	Random	7-13	Ever diagnosed asthma
Brozek, G., et al.	2016	Belarus	Grodno	EUR	LMIC	NA	Random	7-13	Ever diagnosed asthma
Brozek, G., et al.	2016	Ukraine	Ternopil	EUR	LMIC	NA	Random	7-13	Ever diagnosed asthma
Brozek, G., et al.	2016	Ukraine	Ternopil	EUR	LMIC	NA	Random	7-13	Ever diagnosed asthma
Brozek, G., et al.	2016	Poland	Katowice	EUR	HIC	NA	Random	7-13	Ever diagnosed asthma
Brozek, G., et al.	2016	Poland	Katowice	EUR	HIC	NA	Random	7-13	Ever diagnosed asthma
Portnov, B. A., et al.	2012	Israel	Haifa, Qiryat Tivon, Nesher, Qiryat Ata, Qiryat Motzkin, Qiryat Bialik and Qiryat Yam	EUR	HIC	2008-2009	Random	6-15	Ever diagnosed asthma
Chrischilles, E., et al.	2004	US	Iowa	AMR	HIC	1999-2002	Random	6-14	Ever wheezing
Anthracopoulos, M., et al.	2001	Greece	Patras	EUR	HIC	1978, 1991 & 1998	Random	8-10	Current wheezing
de Marco, R., et al.	2012	Italy	Ferrara, Pavia, Pisa, Sassari, Sassuolo, Siracusa, Turin, Udine and Verona.	EUR	HIC	1990-2010	Random	20-44	Newly diagnosed asthma

Author	Year Published	Country	Study site	WHO Region	WB Income Region	Investig ation Date	Sampling Strategy	Age range	Group
Muc, M., et al.	2014	Portugal	Coimbra	EUR	HIC	2012	Random	6-8	Ever wheezing
Tai, A., et al.	2009	Australia	South Australia	WPR	HIC	2006	Random	4-5	Current wheezing
von Maffei, J., et al.	2001	US	Virginia and Connecticut	AMR	HIC	1993-1996	Random	0-18	Ever diagnosed asthma
Tug, T. and Y. Acik	2002	Turkey	Elazig Region	EUR	LMIC	2002	Random	NA	Ever wheezing
Sy, D. Q., et al.	2007	Vietnam	Dalat Highlands	WPR	LMIC	2004	Random	NA	Ever diagnosed asthma
Erhabor, G. E., et al.	2016	Nigeria	Obafemi Awolowo University, IleIfe	AFR	LMIC	2012	Random	15-39 & 22-73	Current wheezing
Chang, H. J., et al.	2012	Canada	Canada	AMR	HIC	2006-2007	Random	6-14 & 15-64	Ever diagnosed asthma
Duhme, H., et al.	1998	Germany	Münster, western Germany, and Greifswald, eastern Ger-many	EUR	HIC	1994-1995	Random	5-8 & 12-15	Ever wheezing
Lin, J., et al.	2018	China	Mainland China (Beijing, Shanghai, Jiangsu, Guangdong, Liaoning, Sichuan, Henan and Shanxi)	WPR	LMIC	2010-2012	Random	15 & above	Ever diagnosed asthma
Lin, R., et al.	2014	China	Qingdao	WPR	LMIC	2010-2011	Random	1-14	Ever diagnosed asthma
Saraclar, Y., et al.	1997	Turkey	Ankara	EUR	LMIC	1991-1992	Random	6-13	Ever diagnosed asthma

Erhabor, G. E., et al.	2006	Nigeria	Ile-Ife	AFR	LMIC	NA	Random	15-35	Current wheezing
Sharma, B. S., et al.	2012	India	Jaipur	SEAR	LMIC	2008-2009	Random	5-15	Ever wheezing
Sole, D., et al.	1999	Brazil	Sao Paulo	AMR	LMIC	1995	Random	6-7 & 13-14	Ever wheezing
Kraai, S., et al.	2013	Venezuela	Warao	AMR	LMIC	2012	Random	2-10	Ever wheezing
Ormerod, L. P., et al.	1999	UK	Blackburn	EUR	HIC	1990-1991	Random	0 & above	Ever wheezing
Solet, J. L., et al.	2019	France	Reunion	EUR	HIC	2016	Random	18-44	Newly diagnosed asthma
Timonen, K. L., et al.	1995	Finland	Kuopio	EUR	HIC	1993-1994	Random	7-12	Newly diagnosed asthma
Saraclar, Y., et al.	1998	Turkey	Ankara	EUR	LMIC	1996	Random	7-14	Ever wheezing
Saraclar, Y., et al.	1998	Turkey	Ankara	EUR	LMIC	1996	Random	7-14	Ever diagnosed asthma
Saraclar, Y., et al.	1998	Turkey	Ankara	EUR	LMIC	1996	Random	7-14	Current wheezing
Saraclar, Y., et al.	1998	Turkey	Ankara	EUR	LMIC	1996	Random	7-14	Newly diagnosed asthma

Table S6. List of the included articles

1. Hedman, J., et al. (1999). "Prevalence of asthma, aspirin intolerance, nasal polyposis and chronic obstructive pulmonary disease in a population-based study." International Journal of Epidemiology 28(4): 717-722.

2. Salem, M. B., et al. (2002). "Prevalence of wheeze among preschool children in Basra governonate, southern Iraq." Eastern Mediterranean Health Journal 8(4-5): 503-508.

3. Obaseki, D. O., et al. (2014). "Low prevalence of asthma in sub Saharan Africa: a cross sectional community survey in a suburban Nigerian town." Respiratory Medicine 108(11): 1581-1588.

4. Horak, E., et al. (2014). "Prevalence of wheezing and atopic diseases in Austrian schoolchildren in conjunction with urban, rural or farm residence." Wiener Klinische Wochenschrift 126(17-18): 532-536.

5. Anderson, H. R., et al. (1994). "Trends in prevalence and severity of childhood asthma." BMJ 308(6944): 1600-1604.

6. Crain, E. F., et al. (1994). "An estimate of the prevalence of asthma and wheezing among inner-city children." Pediatrics 94(3): 356-362.

7. Kirenga, B. J., et al. (2019). "Prevalence and factors associated with asthma among adolescents and adults in Uganda: a general population based survey." BMC Public Health 19(1): 227.

8. Seneviratne, R. and N. S. Gunawardena (2018). "Prevalence and associated factors of wheezing illnesses of children aged three to five years living in under-served settlements of the Colombo Municipal Council in Sri Lanka: a cross-sectional study." BMC Public Health 18(1): 127.

9. Yawn, B. P., et al. (2002). "A longitudinal study of the prevalence of asthma in a community population of school-age children." Journal of Pediatrics 140(5): 576-581.

10. Bjorksten, B., et al. (1998). "Prevalence of childhood asthma, rhinitis and eczema in Scandinavia and Eastern Europe." European Respiratory Journal 12(2): 432-437.

11. Barry, D. M., et al. (1991). "Prevalence of asthma among 12 year old children in New Zealand and South Wales: a comparative survey." Thorax 46(6): 405-409.

12. Akkurt, I., et al. (2003). "Prevalence of asthma and related symptoms in Sivas, Central Anatolia." Journal of Asthma 40(5): 551-556.

13. Isoaho, R., et al. (1994). "Prevalence of asthma in elderly Finns." Journal of Clinical Epidemiology 47(10): 1109-1118.

14. Papageorgiou, N., et al. (1997). "Prevalence of asthma and asthma-like symptoms in Athens, Greece." Respiratory Medicine 91(2): 83-88

15. Brozek, G., et al. (2015). "Increasing prevalence of asthma, respiratory symptoms, and allergic diseases: Four repeated surveys from 1993-2014." Respiratory Medicine 109(8): 982-990.

16. Bechtold, P., et al. (2013). "Assessing paediatric asthma occurrence through dispensed prescription data and questionnaires." European Journal of Public Health 23(5): 873-878.

17. Nriagu, J., et al. (1999). "Prevalence of asthma and respiratory symptoms in south-central Durban, South Africa." European Journal of Epidemiology 15(8): 747-755

18. Anderson, H. R., et al. (2004). "Trends in prevalence of symptoms of asthma, hay fever, and eczema in 12-14 year olds in the British Isles, 1995-2002: questionnaire survey." BMJ 328(7447): 10521053.

19. Mallol, J., et al. (2010). "International prevalence of recurrent wheezing during the first year of life: variability, treatment patterns and use of health resources." Thorax 65(11): 1004-1009

20. Johnson, M., et al. (2005). "Asthma prevalence and severity in Arab American communities in the Detroit area, Michigan." Journal of Immigrant Health 7(3): 165-178.

21. Solis Soto, M. T., et al. (2014). "Prevalence of asthma, rhinitis and eczema symptoms in rural and urban school-aged children from Oropeza Province - Bolivia: a cross-sectional study." BMC Pulmonary Medicine 14: 40

22. Mattei, A., et al. (2007). "[Prevalence of asthma, respiratory symptoms and allergic disorders among adolescents in the province of Aquila]." Epidemiologia e Prevenzione 31(5): 247-252.

23. Lee, S. I., et al. (2001). "Prevalences of symptoms of asthma and other allergic diseases in korean children: a nationwide questionnaire survey." Journal of Korean Medical Science 16(2): 155-164.

24. Lima, W. L., et al. (2012). "[Asthma and associated factors in students 13 and 14 years of age in Sao Luis, Maranhao State, Brazil]." Cadernos de Saude Publica 28(6): 1046-1056

25. Hansen, E. F., et al. (2000). "Increase in prevalence and severity of asthma in young adults in Copenhagen." Thorax 55(10): 833-836.

26. Fukutomi, Y., et al. (2011). "Time trend in the prevalence of adult asthma in Japan: findings from population-based surveys in Fujieda City in 1985, 1999, and 2006." Allergology International 60(4): 443-448.

27. Al Ghobain, M. O., et al. (2012). "Asthma prevalence among 16- to 18-year-old adolescents in Saudi Arabia using the ISAAC questionnaire." BMC Public Health 12: 239.

28. Kausel, L., et al. (2013). "Childhood asthma and allergies in urban, semiurban, and rural residential sectors in Chile." Thescientificworldjournal 2013: 937935

29. von Mutius, E., et al. (1992). "Prevalence of asthma and allergic disorders among children in united Germany: a descriptive comparison." BMJ 305(6866): 1395-1399.

30. Alves, J., et al. (1994). "[Prevalence of asthma in the city of Porto]." Acta Medica Portuguesa 7(1): 21-24.

31. Liao, M. F., et al. (2005). "Prevalence of asthma, rhinitis, and eczema from ISAAC survey of schoolchildren in Central Taiwan." Journal of Asthma 42(10): 833-837.

32. Ehrlich, R. I., et al. (1995). "Prevalence and reliability of asthma symptoms in primary school children in Cape Town." International Journal of Epidemiology 24(6): 1138-1145.

33. Arnedo-Pena, A., et al. (2011). "Sunny hours and variations in the prevalence of asthma in schoolchildren according to the International Study of Asthma and Allergies (ISAAC) Phase III in Spain." International Journal of Biometeorology 55(3): 423-434.

34. Nugmanova, D., et al. (2018). "The prevalence, burden and risk factors associated with bronchial asthma in commonwealth of independent states countries (Ukraine, Kazakhstan and Azerbaijan): results of the CORE study." BMC Pulmonary Medicine 18(1): 110.

35. Cazzola, M., et al. (2011). "The prevalence of asthma and COPD in Italy: a practice-based study." Respiratory Medicine 105(3): 386-391.

36. Heinrich, J., et al. (2002). "Trends in prevalence of atopic diseases and allergic sensitization in children in Eastern Germany." European Respiratory Journal 19(6): 1040-1046.

37. James, A. L., et al. (2010). "Changes in the prevalence of asthma in adults since 1966: the Busselton health study." European Respiratory Journal 35(2): 273-278.

38. Ronchetti, R., et al. (2001). "Is the increase in childhood asthma coming to an end? Findings from three surveys of schoolchildren in Rome, Italy." European Respiratory Journal 17(5): 881-886.

39. Wang, X. S., et al. (2004). "The prevalence of asthma and allergies in Singapore; data from two ISAAC surveys seven years apart." Archives of Disease in Childhood 89(5): 423-426.

40. Bemanin, M. H., et al. (2015). "First report of asthma prevalence in Afghanistan using international standardized methods." Eastern Mediterranean Health Journal 21(3): 194-198.

41. Robertson, C. F., et al. (1991). "Prevalence of asthma in Melbourne schoolchildren: changes over 26 years." BMJ 302(6785): 1116-1118.

42. Asthma and its comorbidities in middle-aged and older adults; the Rotterdam Study

43. Toelle, B. G., et al. (2004). "Prevalence of asthma and allergy in schoolchildren in Belmont, Australia: three cross sectional surveys over 20 years." BMJ 328(7436): 386-387.

44. Brunner, W. M., et al. (2005). "Asthma among rural Minnesota adolescents." Journal of Asthma 42(9): 787-792.

45. Hasnain, S. M., et al. (2009). "Prevalence of asthma and allergic rhinitis among school children of Karachi, Pakistan, 2007." Journal of Asthma 46(1): 86-90.

46. Reis, G. G., et al. (2015). "Prevalence and risk factors for wheezing in Salvador, Brazil: a population-based study." Qjm 108(3): 213-218.

47. Nguyen, T., et al. (2015). "The prevalence of asthma risk and contributing factors in underserved Vietnamese children in Orange County, CA." Journal of Asthma 52(10): 1031-1037.

48. Huurre, T. M., et al. (2004). "Incidence and prevalence of asthma and allergic rhinitis: a cohort study of Finnish adolescents." Journal of Asthma 41(3): 311-317.

49. Ozdemir, N., et al. (2000). "The prevalence of asthma and allergy among university freshmen in Eskisehir, Turkey." Respiratory Medicine 94(6): 536-541.

50. Liao, M. F., et al. (2009). "Prevalence of allergic diseases of schoolchildren in central taiwan. From ISAAC surveys 5 years apart." Journal of Asthma 46(6): 541-545.

51. Strachan, D. P., et al. (1994). "A national survey of asthma prevalence, severity, and treatment in Great Britain." Archives of Disease in Childhood 70(3): 174-178.

52. Weiland, S. K., et al. (1999). "Prevalence of respiratory and atopic disorders among children in the East and West of Germany five years after unification." European Respiratory Journal 14(4): 862-870.

53. Ronmark, E., et al. (2002). "Incidence rates and risk factors for asthma among school children: a 2-year follow-up report from the obstructive lung disease in Northern Sweden (OLIN) studies." Respiratory Medicine 96(12): 1006-1013.

54. Saraclar, Y., et al. (1997). "The prevalence of self-reported asthma and respiratory symptoms in Ankara, Turkey." Respiratory Medicine 91(8): 461-463.

55. Nishima, S., et al. (2009). "Surveys on the prevalence of pediatric bronchial asthma in Japan: a comparison between the 1982, 1992, and 2002 surveys conducted in the same region using the same methodology." Allergology International 58(1): 37-53.

56. Lee, T., et al. (2003). "Asthma prevalence among inner-city Asian American schoolchildren." Public Health Reports 118(3): 215-220.

57. Vanfleteren, L. E., et al. (2012). "The prevalence of chronic obstructive pulmonary disease in Maastricht, the Netherlands." Respiratory Medicine 106(6): 871-874.

58. Renwick, D. S. and M. J. Connolly (1996). "Prevalence and treatment of chronic airways obstruction in adults over the age of 45." Thorax 51(2): 164-168.

59. Matsumoto, K., et al. (2015). "Prevalence of asthma with airflow limitation, COPD, and COPD with variable airflow limitation in older subjects in a general Japanese population: the Hisayama Study." Respiratory Investigation 53(1): 22-29.

60. Carlsson, A. C., et al. (2013). "High prevalence of diagnosis of diabetes, depression, anxiety, hypertension, asthma and COPD in the total population of Stockholm, Sweden - a challenge for public health." BMC Public Health 13: 670.

61. Maia, J. G., et al. (2004). "Prevalence of asthma and asthma symptoms among 13 and 14-year-old schoolchildren, Brazil." Revista de Saude Publica 38(2): 292-299.

62. Kiboneka, A., et al. (2016). "Prevalence of asthma among school children in Gaborone, Botswana." African Health Sciences 16(3): 809-816.

63. Hu, F. B., et al. (1997). "An epidemiological study of asthma prevalence and related factors among young adults." Journal of Asthma 34(1): 67-76.

64. Pakhale, S., et al. (2008). "Prevalence of asthma symptoms in 7th- and 8th-grade school children in a rural region in India." Journal of Asthma 45(2): 117-122.

65. Lawson, J. A., et al. (2017). "Childhood asthma, asthma severity indicators, and related conditions along an urban-rural gradient: a cross-sectional study." BMC Pulmonary Medicine 17(1): 4.

66. Rizwan, S., et al. (2004). "Trends in childhood and parental asthma prevalence in Merseyside, 1991-1998." Journal of Public Health 26(4): 337-342.

67. Freitas, M. S., et al. (2012). "Prevalence of asthma symptoms and associated factors in schoolchildren from Brazilian Amazon islands." Journal of Asthma 49(6): 600-605.

68. Findley, S., et al. (2003). "Elevated asthma and indoor environmental exposures among Puerto Rican children of East Harlem." Journal of Asthma 40(5): 557-569.

69. Simon, P. A., et al. (2003). "Prevalence of childhood asthma and associated morbidity in Los Angeles County: impacts of race/ethnicity and income." Journal of Asthma 40(5): 535-543.

70. Hoffmann, F. (2007). "Prevalence of asthma among German adults: analysis of the German National Telephone Survey." Journal of Asthma 44(6): 433-436.

71. Claudio, L., et al. (2006). "Prevalence of childhood asthma in urban communities: the impact of ethnicity and income." Annals of Epidemiology 16(5): 332-340.

72. Silva Rde, C., et al. (2013). "The prevalence of wheezing and its association with body mass index and abdominal obesity in children." Journal of Asthma 50(3): 267-273.

73. Bryant-Stephens, T., et al. (2012). "Asthma prevalence in Philadelphia: description of two community-based methodologies to assess asthma prevalence in an inner-city population." Journal of Asthma 49(6): 581-585.

74. Goh, D. Y., et al. (1996). "Prevalence and severity of asthma, rhinitis, and eczema in Singapore schoolchildren." Archives of Disease in Childhood 74(2): 131-135.

75. Carrillo, G., et al. (2018). "Asthma prevalence and school-related hazardous air pollutants in the US-Mexico border area." Environmental Research 162: 41-48.

76. Mallol, J., et al. (2010). "Regional variation in asthma symptom prevalence in Latin American children." Journal of Asthma 47(6): 644-650.

77. Romano-Zelekha, O., et al. (2007). "Trends in the prevalence of asthma symptoms and allergic diseases in Israeli adolescents: results from a national survey 2003 and comparison with 1997." Journal of Asthma 44(5): 365-369.

78. El Ftouh, M., et al. (2009). "Paediatric asthma in North Africa: the Asthma Insights and Reality in the Maghreb (AIRMAG) study." Respiratory Medicine 103 Suppl 2: S21-29.

79. Stojanovic-Ristic, S., et al. (2006). "[Prevalence of asthma in college student population]." Srpski Arhiv Za Celokupno Lekarstvo 134(9-10): 398-403.

80. Shamssain, M. H. and N. Shamsian (1999). "Prevalence and severity of asthma, rhinitis, and atopic eczema: the north east study." Archives of Disease in Childhood 81(4): 313-317.

81. Kim, S. Y., et al. (2013). "Increased prevalence of self-reported asthma among Korean adults: an analysis of KNHANES I and IV data." Lung 191(3): 281-288.

82. Romantowski, J., et al. (2015). "Asthma prevalence and risk factors analysis in Tricity university students' group." Pneumonologia i Alergologia Polska 83(5): 359-364.

83. Brugge, D., et al. (2008). "Community-level data suggest that asthma prevalence varies between U.S. and foreign-born black subpopulations." Journal of Asthma 45(9): 785-789.

84. Shimwela, M., et al. (2014). "Asthma prevalence, knowledge, and perceptions among secondary school pupils in rural and urban coastal districts in Tanzania." BMC Public Health 14: 387.

85. Senthilselvan, A., et al. (2016). "Prevalence and risk factors of asthma in First Nations children living on reserves in Canada." Canadian Journal of Public Health. Revue Canadienne de Sante Publique 106(8): e483-488.

86. Ziyab, A. H. (2017). "Prevalence and Risk Factors of Asthma, Rhinitis, and Eczema and Their Multimorbidity among Young Adults in Kuwait: A Cross-Sectional Study." BioMed Research International 2017: 2184193.

87. Kainu, A., et al. (2013). "Increase in prevalence of physician-diagnosed asthma in Helsinki during the Finnish Asthma Programme: improved recognition of asthma in primary care? A crosssectional cohort study." Primary Care Respiratory Journal 22(1): 64-71.

88. Gershon, A. S., et al. (2010). "Trends in asthma prevalence and incidence in Ontario, Canada, 1996-2005: a population study." American Journal of Epidemiology 172(6): 728-736.

89. Gershon, A. S., et al. (2010). "Trends in asthma prevalence and incidence in Ontario, Canada, 1996-2005: a population study." American Journal of Epidemiology 172(6): 728-736.

90. Singh, S., et al. (2016). "Prevalence and severity of asthma among Indian school children aged between 6 and 14 years: associations with parental smoking and traffic pollution." Journal of Asthma 53(3): 238-244.

91. Peroni, D. G., et al. (2009). "Preschool asthma in Italy: prevalence, risk factors and health resource utilization." Respiratory Medicine 103(1): 104-108.

92. Kaur, B., et al. (1998). "Prevalence of asthma symptoms, diagnosis, and treatment in 12-14 year old children across Great Britain (international study of asthma and allergies in childhood, ISAAC UK)." BMJ 316(7125): 118-124.

93. Kuehni, C. E., et al. (2000). "Prevalence of wheeze during childhood: retrospective and prospective assessment." European Respiratory Journal 16(1): 81-85.

94. Delmas, M. C., et al. (2009). "[Prevalence of asthma among children in France]." Archives de Pediatrie 16(9): 1261-1269.

95. Werneck, G., et al. (1999). "Prevalence of asthma and other childhood allergies in Brazilian schoolchildren." Journal of Asthma 36(8): 677-690.

96. Norzila, M. Z., et al. (2000). "Prevalence of childhood asthma and allergy in an inner city Malaysian community: intra-observer reliability of two translated international questionnaires." Medical Journal of Malaysia 55(1): 33-39.

97. Ngui, R., et al. (2011). "Prevalence of bronchial asthma among orang asli in peninsular malaysia." Medical Journal of Malaysia 66(1): 27-31.

98. El Burai Felix, S., et al. (2015). "Asthma prevalence among Hispanic adults in Puerto Rico and Hispanic adults of Puerto Rican descent in the United States - results from two national surveys." Journal of Asthma 52(1): 3-9.

99. Anthracopoulos, M. B., et al. (2007). "Prevalence of asthma among schoolchildren in Patras, Greece: four questionnaire surveys during 1978-2003." Archives of Disease in Childhood 92(3): 209212.

100. Lamnisos, D., et al. (2013). "Prevalence of asthma and allergies in children from the Greek-Cypriot and Turkish-Cypriot communities in Cyprus: a bi-communal cross-sectional study." BMC Public Health 13: 585.

101. Bishwajit, G., et al. (2017). "Burden of asthma, dyspnea, and chronic cough in South Asia." International Journal of Copd 12: 1093-1099.

102. Austin, J. B., et al. (1994). "Prevalence of asthma and wheeze in the Highlands of Scotland." Archives of Disease in Childhood 71(3): 211-216.

103. Moradi-Lakeh, M., et al. (2015). "Prevalence of asthma in Saudi adults: findings from a national household survey, 2013." BMC Pulmonary Medicine 15: 77.

104. Fedortsiv, O., et al. (2012). "Prevalence of childhood asthma, rhinitis, and eczema in the Ternopil region of Ukraine--results of BUPAS study." Advances in Medical Sciences 57(2): 282-289.

105. Trakultivakorn, M., et al. (2007). "Time trends of the prevalence of asthma, rhinitis and eczema in Thai children-ISAAC (International Study of Asthma and Allergies in Childhood) Phase Three." Journal of Asthma 44(8): 609-611.

106. Gupta, D., et al. (2001). "Prevalence of bronchial asthma and association with environmental tobacco smoke exposure in adolescent school children in Chandigarh, north India." Journal of Asthma 38(6): 501-507.

107. Musafiri, S., et al. (2011). "Prevalence of atopy, asthma and COPD in an urban and a rural area of an African country." Respiratory Medicine 105(11): 1596-1605.

108. Kim, J. L., et al. (2014). "Trends in the prevalence of asthma, rhinitis, and eczema in 15 year old adolescents over an 8 year period." Respiratory Medicine 108(5): 701-708.

109. Greenlee, R. T., et al. (2008). "Prevalence of asthma in a general population cohort of farm children: comparison of estimates based on parental report and medical record review." Journal of Agromedicine 13(4): 225-231.

110. Ma, Q. and T. Yang (2017). "[Prevalence and influential factors for asthma among adults in Chinese]." Zhong Nan da Xue Xue Bao. Yi Xue Ban = Journal of Central South University. Medical Sciences 42(9): 1086-1093.

111. Middleton, N., et al. (2014). "Prevalence of asthma and respiratory symptoms in 15-17 year-old Greek-Cypriots by proximity of their community of residence to power plants: Cyprus 2006-07." Public Health 128(3): 288-296.

112. Shakurnia, A. H., et al. (2010). "Prevalence of asthma among schoolchildren in Ahvaz, Islamic Republic of Iran." Eastern Mediterranean Health Journal 16(6): 651-656.

113. Jarvis, D., et al. (1994). "Prevalence of asthma and asthma-like symptoms in young adults living in three east Anglian towns." British Journal of General Practice 44(388): 493-497.

114. Glushkova, A. V. and A. M. Grjibovski (2008). "Prevalence and correlates of asthma among children in central St. Petersburg, Russia: cross-sectional study." Croatian Medical Journal 49(6): 741750.

115. Nystad, W., et al. (1997). "Changing prevalence of asthma in school children: evidence for diagnostic changes in asthma in two surveys 13 yrs apart." European Respiratory Journal 10(5): 10461051.

116. Rose, D., et al. (2006). "Asthma prevalence among US adults, 1998-2000: role of Puerto Rican ethnicity and behavioral and geographic factors." American Journal of Public Health 96(5): 880-888.

117. Jindal, S. K., et al. (2000). "Study of the prevalence of asthma in adults in North India using a standardized field questionnaire." Journal of Asthma 37(4): 345-351.

118. Peat, J. K., et al. (1994). "Changing prevalence of asthma in Australian children." BMJ 308(6944): 1591-1596. OBJECTIVE: To investigate whether prevalence of asthma in children increased in 10 years.

119. Gao, Z., et al. (2008). "Prevalence of asthma and risk factors for asthma-like symptoms in Aboriginal and non-Aboriginal children in the northern territories of Canada." Canadian Respiratory Journal 15(3): 139-145.

120. Jerning, C., et al. (2013). "Asthma and physical activity--a population based study results from the Swedish GA(2)LEN survey." Respiratory Medicine 107(11): 1651-1658.

121. Wehrmeister, F. C. and K. G. Peres (2010). "[Regional inequalities in the prevalence of asthma diagnosis in children: an analysis of the Brazilian National Household Sample Survey, 2003]." Cadernos de Saude Publica 26(9): 1839-1852.

122. Al Ghobain, M. O., et al. (2012). "Asthma prevalence among 16- to 18-year-old adolescents in Saudi Arabia using the ISAAC questionnaire." BMC Public Health 12: 239.

123. Barraza Villarreal, A., et al. (2003). "Risk factors for asthma in school children from Ciudad Juarez, Chihuahua." Journal of Asthma 40(4): 413-423.

124. Tan, T. N., et al. (2006). "Prevalence of asthma and comorbid allergy symptoms in Singaporean preschoolers." Asian Pacific Journal of Allergy & Immunology 24(4): 175-182.

125. Sole, D., et al. (2015). "Prevalence of asthma and related symptoms in adolescents: findings from 3 surveys." Journal of Investigational Allergology & Clinical Immunology 25(1): 73-74.

126. Kabesch, M., et al. (1999). "Lower prevalence of asthma and atopy in Turkish children living in Germany." European Respiratory Journal 13(3): 577-582.

127. Broms, K., et al. (2012). "A nationwide study of asthma incidence rate and its determinants in Swedish pre-school children." European Journal of Epidemiology 27(9): 695-703.

128. Chan-Yeung, M., et al. (2002). "The prevalence of asthma and asthma-like symptoms among adults in rural Beijing, China." European Respiratory Journal 19(5): 853-858.

129. Mvula, M., et al. (2005). "Prevalence of asthma and asthma-like symptoms in inner-city schoolchildren." Journal of Asthma 42(1): 9-16.

130. Joseph, C. L., et al. (1996). "Prevalence of possible undiagnosed asthma and associated morbidity among urban schoolchildren." Journal of Pediatrics 129(5): 735-742.

131. Pekkanen, J., et al. (1997). "Prevalence of asthma symptoms in video and written questionnaires among children in four regions of Finland." European Respiratory Journal 10(8): 1787-1794.

132. Habbick, B. F., et al. (1999). "Prevalence of asthma, rhinitis and eczema among children in 2 Canadian cities: the International Study of Asthma and Allergies in Childhood." CMAJ Canadian Medical Association Journal 160(13): 1824-1828.

133. Shohat, T., et al. (2000). "Prevalence of asthma in 13-14 yr-old schoolchildren across Israel." European Respiratory Journal 15(4): 725-729.

134. Leung, R., et al. (1994). "Prevalence of asthma and wheeze in Hong Kong schoolchildren: an international comparative study." European Respiratory Journal 7(11): 2046-2049.

135. Yeatts, K. B. and C. M. Shy (2001). "Prevalence and consequences of asthma and wheezing in African-American and White adolescents." Journal of Adolescent Health 29(5): 314-319.

136. Arif, A. A., et al. (2005). "A population-based study of asthma, quality of life, and occupation among elderly Hispanic and non-Hispanic whites: a cross-sectional investigation." BMC Public Health 5: 97.

137. Meren, M., et al. (2005). "Low prevalence of asthma in westernizing countries-myth or reality? Prevalence of asthma in Estonia--a report from the "FinEsS" study." Journal of Asthma 42(5): 357365

138. Larsson, L. (1995). "Incidence of asthma in Swedish teenagers: relation to sex and smoking habits." Thorax 50(3): 260-264.

139. Rona, R. J., et al. (1995). "Trends in the prevalence of asthma in Scottish and English primary school children 1982-92." Thorax 50(9): 992-993.

140. Mellinger-Birdsong, A. K., et al. (2003). "Prevalence and impact of asthma in children, Georgia, 2000." American Journal of Preventive Medicine 24(3): 242-248.

141. Dow, L., et al. (2001). "Prevalence of untreated asthma in a population sample of 6000 older adults in Bristol, UK." Thorax 56(6): 472-476.

142. Unlu, M., et al. (2002). "The prevalence of asthma among secondary school students in Afyon, Turkey." Asian Pacific Journal of Allergy & Immunology 20(1): 1-6.

143. Al Ghobain, M. O., et al. (2018). "Asthma prevalence among adults in Saudi Arabia." Saudi Medical Journal 39(2): 179-184.

144. McCann, D., et al. (2002). "The prevalence and management of asthma in primary-aged schoolchildren in the south of England." Health Education Research 17(2): 181-194.

145. Pallasaho, P., et al. (2002). "Prevalence and risk factors for asthma and chronic bronchitis in the capitals Helsinki, Stockholm, and Tallinn." Respiratory Medicine 96(10): 759-769.

146. Khan, A. A., et al. (2014). "Burden of asthma among children in a developing megacity: childhood asthma study, Pakistan." Journal of Asthma 51(9): 891-899.

147. Aberle, N., et al. (2018). "Allergic Diseases and Atopy Among Schoolchildren in Eastern Croatia." Acta Clinica Croatica 57(1): 82-90.

148. Valery, P. C., et al. (2001). "High prevalence of asthma in five remote indigenous communities in Australia." European Respiratory Journal 17(6): 1089-1096

149. Kurukulaaratchy, R. J., et al. (2002). "The prevalence of asthma and wheezing illnesses amongst 10-year-old schoolchildren." Respiratory Medicine 96(3): 163-169.

150. Leung, R. and P. Ho (1994). "Asthma, allergy, and atopy in three south-east Asian populations." Thorax 49(12): 1205-1210.

151. Letamo, G., et al. (2017). "Prevalence and correlates of self-reported chronic non-communicable diseases in Botswana: a cross-sectional study." International Health 9(1): 11-19.

152. Yoshihara, S., et al. (2016). "Prevalence of childhood asthma in Ulaanbaatar, Mongolia in 2009." Allergology International 65(1): 62-67.

153. Levesque, B., et al. (2004). "Asthma and allergic rhinitis in Quebec children." Canadian Respiratory Journal 11(5): 343-348.

154. Schuh, C., et al. (2015). "The prevalence of asthma and atopy in schoolchildren from Porto Alegre, Brazil, has plateaued." Respiratory Medicine 109(3): 308-311.

155. Cakir, E., et al. (2010). "The prevalence and risk factors of asthma and allergic diseases among working adolescents." Asian Pacific Journal of Allergy & Immunology 28(2-3): 122-129.

156. Zivkovic, Z., et al. (2010). "Prevalence of childhood asthma and allergies in Serbia and Montenegro." World Journal of Pediatrics 6(4): 331-336.

157. Hessel, P. A., et al. (2001). "The epidemiology of childhood asthma in Red Deer and Medicine Hat, Alberta." Canadian Respiratory Journal 8(3): 139-146.

158. Stout, J. W., et al. (2001). "Differences in asthma prevalence between samples of American Indian and Alaska Native children." Public Health Reports 116(1): 51-57.

159. Chhabra, S. K., et al. (1998). "Prevalence of bronchial asthma in schoolchildren in Delhi." Journal of Asthma 35(3): 291-296.

160. Jain, A., et al. (2010). "Prevalence of bronchial asthma in rural Indian children: a cross sectional study from South India." Indian Journal of Pediatrics 77(1): 31-35.
161. Yemaneberhan, H., et al. (1997). "Prevalence of wheeze and asthma and relation to atopy in urban and rural Ethiopia." Lancet 350(9071): 85-90.

162. Pite, H., et al. (2014). "Prevalence of asthma and its association with rhinitis in the elderly." Respiratory Medicine 108(8): 1117-1126.

163. Ponsonby, A. L., et al. (2008). "A temporal decline in asthma but not eczema prevalence from 2000 to 2005 at school entry in the Australian Capital Territory with further consideration of country of birth." International Journal of Epidemiology 37(3): 559-569.

164. Gonzalez-Garcia, M., et al. (2015). "Prevalence, risk factors and underdiagnosis of asthma and wheezing in adults 40 years and older: A population-based study." Journal of Asthma 52(8): 823-830.

165. Teeratakulpisarn, J., et al. (2000). "Survey of the prevalence of asthma, allergic rhinitis and eczema in schoolchildren from Khon Kaen, Northeast Thailand. an ISAAC study. International Study of Asthma and Allergies in Childhood." Asian Pacific Journal of Allergy & Immunology 18(4): 187-194.

166. Leung, R., et al. (1997). "Prevalence of asthma and allergy in Hong Kong schoolchildren: an ISAAC study." European Respiratory Journal 10(2): 354-360.

167. Ding, Y. P., et al. (2012). "An epidemiology study of bronchial asthma in the Li ethnic group in China." Asian Pacific Journal of Tropical Medicine 5(2): 157-161.

168. Dennis, R. J., et al. (2012). "Prevalence of asthma and other allergic conditions in Colombia 2009-2010: a cross-sectional study." BMC Pulmonary Medicine 12: 17.

169. Zhu, W. J., et al. (2015). "Prevalence and Treatment of Children's Asthma in Rural Areas Compared with Urban Areas in Beijing." Chinese Medical Journal 128(17): 2273-2277.

170. Faniran, A. O., et al. (1999). "Prevalence of atopy, asthma symptoms and diagnosis, and the management of asthma: comparison of an affluent and a non-affluent country." Thorax 54(7): 606-610.

171. Nordlund, B., et al. (2014). "Prevalence of severe childhood asthma according to the WHO." Respiratory Medicine 108(8): 1234-1237.

172. Nelson, K. A., et al. (2009). "Asthma prevalence in low-income urban elementary school students in St. Louis, 1992 and 2004." Journal of Pediatrics 154(1): 111-115.

173. Sole, D., et al. (2015). "Prevalence of asthma and allergic diseases in adolescents: nine-year follow-up study (2003-2012)." Jornal de Pediatria 91(1): 30-35.

174. Zollner, I. K., et al. (2005). "No increase in the prevalence of asthma, allergies, and atopic sensitisation among children in Germany: 1992-2001." Thorax 60(7): 545-548.

175. Wanlapakorn, N., et al. (2014). "Prevalence of asthma, level of control and factors associated with asthma control in Thai elementary school students in Bangkok." Asian Pacific Journal of Allergy & Immunology 32(4): 287-292.

176. Demir, A. U., et al. (2010). "Asthma and allergic diseases in school children from 1992 to 2007 with incidence data." Journal of Asthma 47(10): 1128-1135.

177. Wang, H. Y., et al. (2008). "Prevalence of asthma among Chinese adolescents living in Canada and in China." CMAJ Canadian Medical Association Journal 179(11): 1133-1142.

178. Wang, H. Y., et al. (2008). "Prevalence of asthma among Chinese adolescents living in Canada and in China." CMAJ Canadian Medical Association Journal 179(11): 1133-1142.

179. Pallasaho, P., et al. (1999). "Increasing prevalence of asthma but not of chronic bronchitis in Finland? Report from the FinEsS-Helsinki Study." Respiratory Medicine 93(11): 798-809

180. Tarraf, H., et al. (2018). "Prevalence of asthma among the adult general population of five Middle Eastern countries: results of the SNAPSHOT program." BMC Pulmonary Medicine 18(1): 68.

181. Al-Thamiri, D., et al. (2005). "Asthma prevalence and severity among primary-school children in Baghdad." Eastern Mediterranean Health Journal 11(1-2): 79-86.

182. Roncada, C., et al. (2016). "Burden of asthma among inner-city children from Southern Brazil." Journal of Asthma 53(5): 498-504.

183. Peat, J. K., et al. (1992). "Prevalence of asthma in adults in Busselton, Western Australia." BMJ 305(6865): 1326-1329

184. Ekici, A., et al. (2012). "Prevalence of self-reported asthma in urban and rural areas of Turkey." Journal of Asthma 49(5): 522-526.

185. Palvo, F., et al. (2008). "Risk factors of childhood asthma in Sao Jose do Rio Preto, Sao Paulo, Brazil." Journal of Tropical Pediatrics 54(4): 253-257.

186. Tang, S. P., et al. (2015). "Trends in prevalence and risk factors of childhood asthma in Fuzhou, a city in Southeastern China." Journal of Asthma 52(1): 10-15.

187. Musharrafieh, U., et al. (2009). "Prevalence of asthma, allergic rhinitis and eczema among Lebanese adolescents." Journal of Asthma 46(4): 382-387.

188. Dales, R. E., et al. (1994). "Prevalence of childhood asthma across Canada." International Journal of Epidemiology 23(4): 775-781.

189. Bartlett, E., et al. (2013). "Sources and prevalence of self-reported asthma diagnoses in adults in urban and rural settings of Bangladesh." Global Public Health 8(1): 79-89.

190. Perez-Perdomo, R., et al. (2003). "Prevalence and correlates of asthma in the Puerto Rican population: Behavioral Risk Factor Surveillance System, 2000." Journal of Asthma 40(5): 465-474.

191. Nafti, S., et al. (2009). "Prevalence of asthma in North Africa: the Asthma Insights and Reality in the Maghreb (AIRMAG) study." Respiratory Medicine 103 Suppl 2: S2-11.

192. Manfreda, J., et al. (2001). "Prevalence of asthma symptoms among adults aged 20-44 years in Canada." CMAJ Canadian Medical Association Journal 164(7): 995-1001.

193. Linehan, M. F., et al. (2009). "Prevalence of respiratory symptoms, features of asthma, and characteristics associated with respiratory disease, in 6-11 year olds in Manchester." Primary Care Respiratory Journal 18(1): 21-26.

194. Song, N., et al. (2014). "Prevalence, severity and risk factors of asthma, rhinitis and eczema in a large group of Chinese schoolchildren." Journal of Asthma 51(3): 232-242.

195. Sole, D., et al. (2014). "Asthma in children and adolescents in Brazil: contribution of the International Study of Asthma and Allergies in Childhood (ISAAC)." Revista Paulista de Pediatria 32(1): 114-125.

196. Ronmark, E., et al. (2001). "Incidence and remission of asthma in schoolchildren: report from the obstructive lung disease in northern Sweden studies." Pediatrics 107(3): E37.

197. Brozek, G., et al. (2016). "Childhood asthma prevalence and risk factors in three Eastern European countries--the Belarus, Ukraine, Poland Asthma Study (BUPAS): an international prevalence study." BMC Pulmonary Medicine 16: 11.

198. Portnov, B. A., et al. (2012). "High prevalence of childhood asthma in Northern Israel is linked to air pollution by particulate matter: evidence from GIS analysis and Bayesian Model Averaging." International Journal of Environmental Health Research 22(3): 249-269.

199. Chrischilles, E., et al. (2004). "Asthma prevalence and morbidity among rural Iowa schoolchildren." Journal of Allergy & Clinical Immunology 113(1): 66-71.

200. Anthracopoulos, M., et al. (2001). "Prevalence of asthma among schoolchildren in Patras, Greece: three surveys over 20 years." Thorax 56(7): 569-571.

201. de Marco, R., et al. (2012). "Trends in the prevalence of asthma and allergic rhinitis in Italy between 1991 and 2010." European Respiratory Journal 39(4): 883-892.

202. Muc, M., et al. (2014). "Prevalence of asthma and rhinitis symptoms among children living in Coimbra, Portugal." Revista Portuguesa de Pneumologia 20(4): 208-210.

203. Tai, A., et al. (2009). "Prevalence of asthma symptoms and atopic disorders in preschool children and the trend over a decade." Journal of Asthma 46(4): 343-346.

204. von Maffei, J., et al. (2001). "Risk factors for asthma prevalence among urban and nonurban African American children." Journal of Asthma 38(7): 555-564.

205. Tug, T. and Y. Acik (2002). "Prevalence of asthma, asthma-like and allergic symptoms in the urban and rural adult population in Eastern Turkey." Asian Pacific Journal of Allergy & Immunology 20(4): 209-215.

206. Sy, D. Q., et al. (2007). "Prevalence of asthma and asthma-like symptoms in Dalat Highlands, Vietnam." Singapore Medical Journal 48(4): 294-303.

207. Erhabor, G. E., et al. (2016). "Asthma in a university campus: a survey of students and staff of Obafemi Awolowo University, Ile-Ife, Nigeria." Journal of Asthma 53(1): 30-36.

208. Chang, H. J., et al. (2012). "Prevalence and risk factors of asthma in off-reserve Aboriginal children and adults in Canada." Canadian Respiratory Journal 19(6): e68-74.

209. Duhme, H., et al. (1998). "Asthma and allergies among children in West and East Germany: a comparison between Munster and Greifswald using the ISAAC phase I protocol. International Study of Asthma and Allergies in Childhood." European Respiratory Journal 11(4): 840-847.

210. Lin, J., et al. (2018). "Prevalence and risk factors of asthma in mainland China: The CARE study." Respiratory Medicine 137: 48-54.

211. Lin, R., et al. (2014). "Significant rise of the prevalence and clinical features of childhood asthma in Qingdao China: cluster sampling investigation of 10,082 children." BMC Public Health 14: 1002.

212. Saraclar, Y., et al. (1997). "Prevalence of allergic diseases and influencing factors in primary-school children in the Ankara Region of Turkey." Journal of Asthma 34(1): 23-30.

213. Erhabor, G. E., et al. (2006). "Prevalence of asthma symptoms among university students 15 to 35 years of age in Obafemi Awolowo University, Ile-Ife, Osun State." Journal of Asthma 43(2): 161164.

214. Sharma, B. S., et al. (2012). "Prevalence of asthma in urban school children in Jaipur, Rajasthan." Indian Pediatrics 49(10): 835-836.

215. Sole, D., et al. (1999). "Prevalence of asthma and related symptoms in school-age children in Sao Paulo, Brazil--International Study of Asthma and Allergies in Children (ISAAC)." Journal of Asthma 36(2): 205-212.

216. Kraai, S., et al. (2013). "High prevalence of asthma symptoms in Warao Amerindian children in Venezuela is significantly associated with open-fire cooking: a cross-sectional observational study." Respiratory Research 14: 76.

217. Ormerod, L. P., et al. (1999). "Prevalence of asthma and 'probable' asthma in the Asian population in Blackburn, U.K." Respiratory Medicine 93(1): 16-20.

218. Solet, J. L., et al. (2019). "A cross sectional survey to estimate prevalence and associated factors of asthma on Reunion Island, Indian Ocean." BMC Public Health 19(1): 663.

219. Timonen, K. L., et al. (1995). "Prevalence and characteristics of children with chronic respiratory symptoms in eastern Finland." European Respiratory Journal 8(7): 1155-1160.

220. Saraclar, Y., et al. (1998). "Prevalence of asthma symptoms in school children in Ankara, Turkey." Respiratory Medicine 92(2): 203-207.

Appendix 5. Estimation of the regional and national numbers of people with asthma in 2019

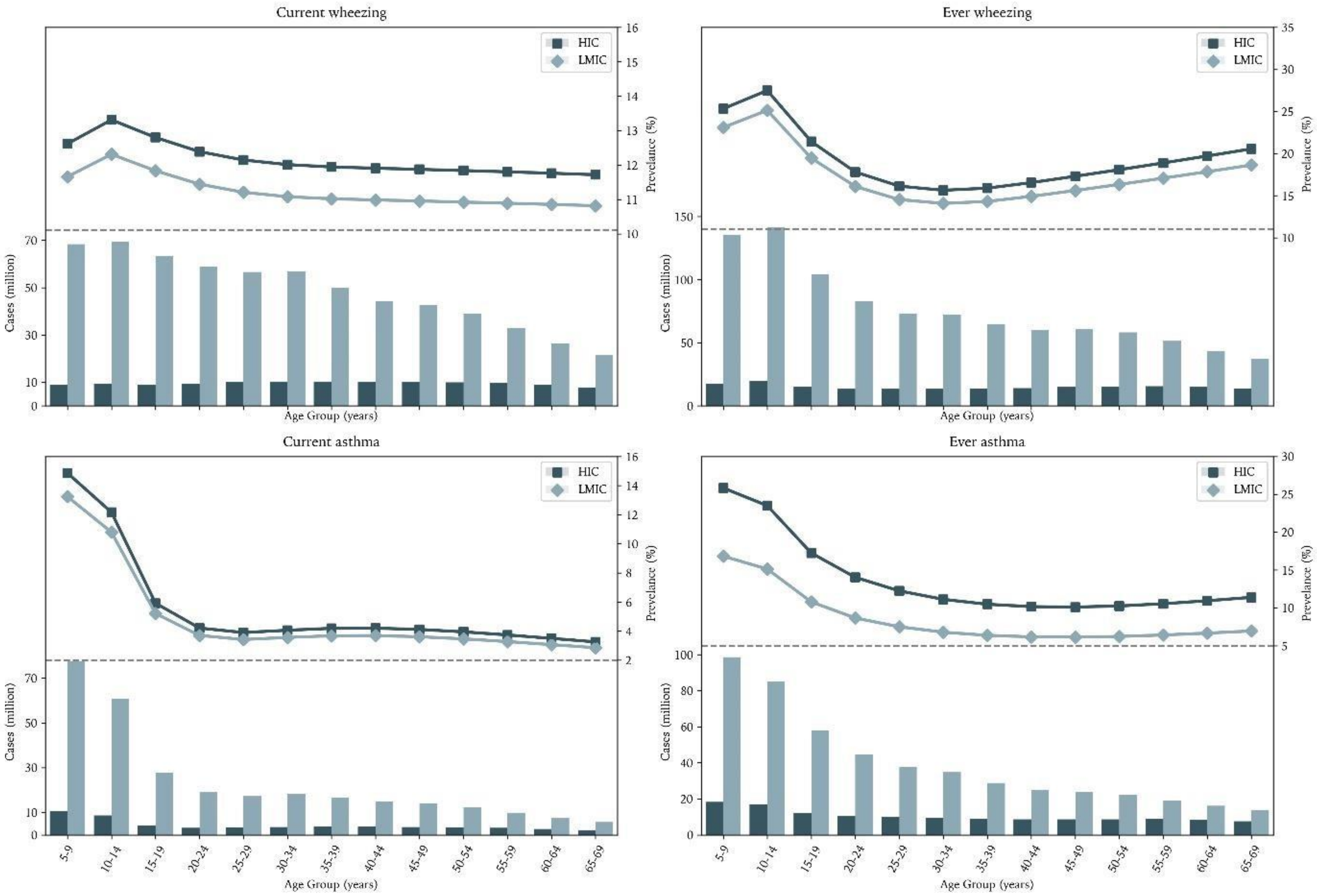

Figure S3. The age-specific prevalence (points) and number of cases (bar) of asthma in 2019, by income

Table S7. The estimated numbers of people with current wheezing, ever wheezing, current asthma and ever asthma in HICs, LMICs and worldwide in 2019, by age group and sex

Age group	Male			Female			Overall		
	HIC	LMIC	Worldwide	HIC	LMIC	Worldwide	HIC	LMIC	Worldwide
**Current wheezing (million, 95% CI)**									
5-9	4.76	36.90	41.67	4.15	31.54	35.69	8.91	68.44	77.36
years	(3.88-5.82)	(29.56-45.77)	(33.44-51.58)	(3.37-5.08)	(25.20-39.23)	(28.57-44.31)	(7.25-10.89)	(54.76-85.00)	(62.01-95.89)
10-14	5.10	37.42	42.52	4.44	31.89	36.33	9.54	69.31	78.85
years	(4.16-6.22)	(30.00-46.36)	(34.15-52.58)	(3.61-5.43)	(25.50-39.62)	(29.11-45.05)	(7.77-11.65)	(55.50-85.98)	(63.26-97.63)
15-19	4.84	34.23	39.06	4.21	29.11	33.32	9.05	63.33	72.38
years	(3.94-5.90)	(27.41-42.45)	(31.34-48.36)	(3.42-5.16)	(23.25-36.21)	(26.67-41.37)	(7.36-11.06)	(50.66-78.66)	(58.02-89.72)
20-24	5.04	31.79	36.83	4.33	27.21	31.54	9.37	59.00	68.37
years	(4.07-6.19)	(25.30-39.68)	(29.37-45.86)	(3.50-5.34)	(21.60-34.06)	(25.10-39.40)	(7.57-11.53)	(46.90-73.73)	(54.47-85.26)
25-29	5.51	30.36	35.87	4.62	26.26	30.88	10.12	56.62	66.75
years	(4.43-6.80)	(24.04-38.07)	(28.47-44.87)	(3.71-5.72)	(20.75-33.03)	(24.45-38.74)	(8.14-12.51)	(44.79-71.10)	(52.93-83.61)
30-34	5.61	30.24	35.85	4.71	26.63	31.35	10.32	56.88	67.20
years	(4.50-6.93)	(23.90-38.00)	(28.41-44.93)	(3.78-5.85)	(21.00-33.56)	(24.78-39.40)	(8.28-12.78)	(44.90-71.55)	(53.18-84.33)
35-39	5.56	26.37	31.93	4.79	23.50	28.29	10.35	49.86	60.21
years	(4.47-6.88)	(20.84-33.13)	(25.31-40.00)	(3.84-5.94)	(18.53-29.61)	(22.37-35.55)	(8.30-12.82)	(39.37-62.73)	(47.67-75.55)
40-44	5.45	23.30	28.76	4.75	20.97	25.72	10.20	44.27	54.47
years	(4.38-6.74)	(18.42-29.27)	(22.80-36.01)	(3.81-5.89)	(16.54-26.42)	(20.34-32.31)	(8.19-12.63)	(34.96-55.69)	(43.15-68.32)
45-49	5.44	22.32	27.76	4.79	20.22	25.01	10.23	42.54	52.76
years	(4.37-6.73)	(17.64-28.05)	(22.00-34.78)	(3.84-5.94)	(15.94-25.48)	(19.77-31.42)	(8.20-12.67)	(33.57-53.53)	(41.78-66.20)
50-54	5.27	20.29	25.57	4.72	18.67	23.39	10.00	38.96	48.96
years	(4.23-6.53)	(16.01-25.54)	(20.24-32.07)	(3.78-5.87)	(14.70-23.56)	(18.47-29.43)	(8.01-12.40)	(30.71-49.10)	(38.72-61.50)
55-59	5.11	16.96	22.06	4.68	15.82	20.50	9.78	32.77	42.56

years	(4.09-6.34)	(13.35-21.38)	(17.44-27.72)	(3.73-5.82)	(12.42-20.01)	(16.16-25.83)	(7.82-12.16)	(25.77-41.39)	(33.59-53.55)
60-64	4.57	13.45	18.03	4.35	12.92	17.27	8.93	26.37	35.30
years	(3.65-5.69)	(10.56-17.01)	(14.21-22.71)	(3.46-5.43)	(10.11-16.39)	(13.58-21.82)	(7.11-11.13)	(20.67-33.40)	(27.79-44.53)
	3.92	10.84	14.76	3.92	10.79	14.71	7.84	21.63	29.47

Age group	Male			Female			Overall		
	HIC	LMIC	Worldwide	HIC	LMIC	Worldwide	HIC	LMIC	Worldwide
65-69 years	(3.11-4.90)	(8.48-13.76)	(11.59-18.66)	(3.11-4.92)	(8.41-13.73)	(11.52-18.65)	(6.22-9.81)	(16.89-27.49)	(23.12-37.31)
Overall (5-69 years)	66.17 (53.27-81.66)	334.48 (265.50-418.47)	400.65 (318.77-500.13)	58.47 (46.94-72.38)	295.52 (233.95-370.90)	353.99 (280.90-443.28)	124.64 (100.21-154.04)	630.00 (499.46-789.37)	754.64 (599.67-943.41)
**Ever wheezing (million, 95% CI)**									
5-9 years	9.59 (7.76-11.67)	73.35 (59.57-89.14)	82.94 (67.33-100.82)	8.31 (6.68-10.18)	62.26 (50.29-76.15)	70.57 (56.97-86.34)	17.90 (14.44-21.85)	135.61 (109.86-165.30)	153.51 (124.30-187.15)
10-14 years	10.55 (8.59-12.76)	76.59 (62.52-92.55)	87.14 (71.11-105.31)	9.16 (7.41-11.15)	64.94 (52.70-79.01)	74.10 (60.11-90.16)	19.71 (16.00-23.92)	141.53 (115.22-171.56)	161.25 (131.22-195.47)
15-19 years	8.14 (6.51-10.03)	56.59 (45.49-69.59)	64.73 (52.00-79.62)	7.02 (5.58-8.71)	47.57 (38.05-58.82)	54.58 (43.64-67.53)	15.15 (12.09-18.74)	104.16 (83.54-128.41)	119.31 (95.64-147.15)
20-24 years	7.29 (5.75-9.13)	45.03 (35.72-56.17)	52.32 (41.47-65.30)	6.18 (4.85-7.78)	37.94 (29.98-47.57)	44.13 (34.83-55.36)	13.47 (10.60-16.91)	82.97 (65.70-103.74)	96.44 (76.30-120.65)
25-29 years	7.37 (5.76-9.31)	39.68 (31.23-49.93)	47.05 (36.99-59.24)	6.08 (4.73-7.72)	33.73 (26.44-42.64)	39.81 (31.17-50.36)	13.44 (10.49-17.03)	73.42 (57.67-92.57)	86.86 (68.16-109.60)
30-34 years	7.35 (5.73-9.32)	38.74 (30.40-48.90)	46.10 (36.13-58.22)	6.08 (4.72-7.75)	33.52 (26.20-42.50)	39.60 (30.92-50.25)	13.44 (10.45-17.07)	72.26 (56.60-91.39)	85.70 (67.05-108.47)
35-39 years	7.45 (5.82-9.44)	34.52 (27.10-43.54)	41.98 (32.92-52.98)	6.32 (4.91-8.04)	30.24 (23.65-38.31)	36.55 (28.55-46.35)	13.77 (10.72-17.48)	64.76 (50.75-81.85)	78.53 (61.47-99.33)
40-44 years	7.63 (5.97-9.64)	31.89 (25.08-40.12)	39.52 (31.05-49.76)	6.55 (5.10-8.31)	28.22 (22.11-35.68)	34.76 (27.21-43.99)	14.18 (11.06-17.95)	60.10 (47.20-75.79)	74.28 (58.26-93.75)

Age group	Male			Female			Overall		
	HIC	LMIC	Worldwide	HIC	LMIC	Worldwide	HIC	LMIC	Worldwide
45-49 years	7.98 (6.25-10.05)	32.04 (25.25-40.23)	40.02 (31.50-50.28)	6.93 (5.40-8.78)	28.57 (22.42-36.06)	35.50 (27.82-44.84)	14.90 (11.65-18.83)	60.62 (47.67-76.29)	75.52 (59.32-95.12)
50-54 years	8.11 (6.36-10.20)	30.56 (24.10-38.32)	38.66 (30.46-48.52)	7.16 (5.59-9.07)	27.70 (21.75-34.92)	34.86 (27.34-43.98)	15.27 (11.95-19.27)	58.26 (45.85-73.23)	73.53 (57.80-92.50)
55-59 years	8.22 (6.45-10.33)	26.77 (21.11-33.54)	34.99 (27.56-43.87)	7.44 (5.81-9.41)	24.63 (19.33-31.03)	32.07 (25.14-40.44)	15.66 (12.26-19.73)	51.40 (40.45-64.57)	67.05 (52.71-84.31)
60-64 years	7.71 (6.05-9.68)	22.25 (17.54-27.88)	29.96 (23.59-37.56)	7.25 (5.66-9.17)	21.10 (16.55-26.59)	28.35 (22.21-35.76)	14.96 (11.71-18.85)	43.35 (34.09-54.47)	58.31 (45.80-73.32)
65-69 years	6.91 (5.42-8.68)	18.79 (14.79-23.55)	25.70 (20.21-32.23)	6.85 (5.34-8.66)	18.47 (14.47-23.30)	25.32 (19.80-31.95)	13.76 (10.76-17.33)	37.26 (29.25-46.85)	51.02 (40.01-64.19)
Overall (5-69 years)	104.30 (82.42-130.25)	526.80 (419.89-653.46)	631.10 (502.31-783.71)	91.30 (71.78-114.72)	458.89 (363.94-572.58)	550.20 (435.72-687.30)	195.60 (154.20-244.97)	985.70 (783.83-1,226.04)	1,181.30 (938.03-1,471.01)
**Current asthma (million, 95% CI)**									
5-9 years	5.51 (4.10-7.30)	41.18 (24.26-67.07)	46.70 (28.36-74.37)	4.98 (3.70-6.61)	36.51 (21.44-59.76)	41.49 (25.14-66.38)	10.50 (7.80-13.92)	77.69 (45.70-126.83)	88.19 (53.50-140.75)
10-14 years	4.59 (3.40-6.12)	32.30 (18.81-53.55)	36.88 (22.21-59.66)	4.13 (3.05-5.52)	28.49 (16.55-47.43)	32.62 (19.61-52.95)	8.72 (6.45-11.64)	60.79 (35.37-100.97)	69.50 (41.82-112.61)
15-19 years	2.20 (1.61-3.00)	14.86 (8.49-25.56)	17.06 (10.09-28.56)	1.98 (1.44-2.70)	13.04 (7.44-22.48)	15.02 (8.88-25.18)	4.18 (3.05-5.71)	27.90 (15.93-48.03)	32.08 (18.98-53.74)
20-24 years	1.69 (1.21-2.35)	10.13 (5.72-17.72)	11.82 (6.92-20.07)	1.50 (1.07-2.08)	8.94 (5.04-15.67)	10.44 (6.11-17.75)	3.18 (2.28-4.43)	19.07 (10.76-33.39)	22.26 (13.03-37.82)
25-29 years	1.74 (1.24-2.44)	9.14 (5.14-16.07)	10.89 (6.37-18.52)	1.51 (1.07-2.11)	8.16 (4.58-14.36)	9.66 (5.65-16.47)	3.25 (2.31-4.56)	17.30 (9.72-30.43)	20.55 (12.02-34.99)
30-34 years	1.87 (1.32-2.62)	9.59 (5.38-16.85)	11.45 (6.71-19.47)	1.62 (1.15-2.27)	8.71 (4.89-15.33)	10.33 (6.03-17.61)	3.49 (2.47-4.89)	18.29 (10.27-32.18)	21.78 (12.74-37.08)

35-39 years	1.92 (1.36-2.70)	8.67 (4.87-15.25)	10.60 (6.23-17.95)	1.71 (1.21-2.40)	7.98 (4.47-14.05)	9.68 (5.68-16.45)	3.63 (2.57-5.10)	16.65 (9.34-29.30)	20.28 (11.91-34.40)
40-44 years	1.90 (1.34-2.67)	7.71 (4.32-13.57)	9.61 (5.67-16.24)	1.70 (1.20-2.40)	7.16 (4.01-12.62)	8.86 (5.22-15.02)	3.60 (2.55-5.06)	14.87 (8.34-26.19)	18.47 (10.88-31.25)
45-49 years	1.86 (1.31-2.61)	7.25 (4.06-12.77)	9.11 (5.37-15.38)	1.69 (1.19-2.37)	6.78 (3.79-11.95)	8.46 (4.98-14.33)	3.54 (2.50-4.99)	14.02 (7.86-24.72)	17.57 (10.36-29.71)
50-54 years	1.73 (1.23-2.44)	6.34 (3.55-11.18)	8.08 (4.78-13.62)	1.60 (1.13-2.25)	6.02 (3.37-10.63)	7.62 (4.50-12.89)	3.33 (2.36-4.69)	12.37 (6.92-21.82)	15.70 (9.28-26.51)
55-59 years	1.59 (1.13-2.24)	5.03 (2.82-8.87)	6.62 (3.94-11.12)	1.50 (1.06-2.12)	4.84 (2.71-8.55)	6.34 (3.77-10.67)	3.10 (2.19-4.36)	9.87 (5.52-17.43)	12.96 (7.71-21.79)
	1.34	3.75	5.09	1.31	3.71	5.03	2.65	7.46	10.11

Age group	Male			Female			Overall		
	HIC	LMIC	Worldwide	HIC	LMIC	Worldwide	HIC	LMIC	Worldwide
60-64 years	(0.95-1.89)	(2.10-6.62)	(3.04-8.51)	(0.93-1.85)	(2.08-6.57)	(3.00-8.42)	(1.88-3.74)	(4.17-13.19)	(6.05-16.93)
65-69 years	1.07 (0.76-1.51)	2.82 (1.58-5.00)	3.90 (2.34-6.51)	1.11 (0.78-1.56)	2.90 (1.62-5.13)	4.00 (2.40-6.69)	2.18 (1.54-3.08)	5.72 (3.20-10.13)	7.90 (4.74-13.20)
Overall (5-69 years)	29.02 (20.95-39.90)	158.77 (91.10-270.08)	187.79 (112.05-309.97)	26.34 (18.99-36.27)	143.23 (81.99-244.53)	169.56 (100.98-280.80)	55.35 (39.93-76.16)	302.00 (173.09-514.61)	357.35 (213.03-590.77)
**Ever asthma (million, 95% CI)**									
5-9 years	8.94 (7.34-10.76)	48.52 (38.95-59.90)	57.45 (46.29-70.66)	9.31 (7.68-11.13)	50.19 (40.45-61.68)	59.50 (48.14-72.81)	18.25 (15.02-21.89)	98.71 (79.40-121.58)	116.95 (94.43-143.47)
10-14 years	8.24 (6.73-9.98)	41.89 (33.50-51.95)	50.13 (40.23-61.94)	8.60 (7.06-10.35)	43.27 (34.73-53.43)	51.86 (41.79-63.78)	16.84 (13.79-20.33)	85.16 (68.23-105.38)	102.00 (82.02-125.71)
15-19 years	5.94 (4.78-7.31)	28.34 (22.45-35.57)	34.28 (27.23-42.88)	6.25 (5.05-7.66)	29.38 (23.33-36.75)	35.63 (28.39-44.41)	12.19 (9.83-14.97)	57.72 (45.78-72.32)	69.91 (55.62-87.28)
20-24 years	5.19 (4.15-6.45)	21.8 (17.19-27.52)	26.99 (21.34-33.97)	5.42 (4.35-6.70)	22.82 (18.03-28.72)	28.24 (22.37-35.42)	10.61 (8.50-13.15)	44.62 (35.22-56.24)	55.23 (43.71-69.39)

25-29 years	5.05 (4.02-6.30)	18.39 (14.46-23.28)	23.43 (18.48-29.58)	5.15 (4.11-6.40)	19.48 (15.34-24.60)	24.62 (19.46-31.00)	10.19 (8.13-12.70)	37.86 (29.80-47.88)	48.06 (37.93-60.58)
30-34 years	4.72 (3.75-5.90)	16.74 (13.14-21.24)	21.46 (16.89-27.14)	4.83 (3.85-6.03)	18.07 (14.21-22.87)	22.90 (18.06-28.90)	9.55 (7.60-11.93)	34.81 (27.35-44.11)	44.36 (34.95-56.04)
35-39 years	4.42 (3.51-5.55)	13.76 (10.79-17.47)	18.18 (14.30-23.02)	4.65 (3.69-5.81)	15.04 (11.82-19.06)	19.68 (15.51-24.87)	9.07 (7.20-11.35)	28.80 (22.61-36.54)	37.87 (29.81-47.89)
40-44 years	4.22 (3.34-5.29)	11.81 (9.26-15.01)	16.03 (12.60-20.30)	4.48 (3.56-5.61)	13.04 (10.24-16.53)	17.52 (13.80-22.14)	8.70 (6.90-10.90)	24.84 (19.50-31.54)	33.54 (26.40-42.44)
45-49 years	4.19 (3.32-5.26)	11.27 (8.84-14.33)	15.47 (12.16-19.59)	4.51 (3.58-5.64)	12.53 (9.84-15.89)	17.03 (13.42-21.53)	8.70 (6.90-10.90)	23.80 (18.67-30.21)	32.50 (25.58-41.11)
50-54 years	4.14 (3.28-5.19)	10.42 (8.17-13.25)	14.56 (11.45-18.43)	4.52 (3.59-5.65)	11.77 (9.24-14.92)	16.28 (12.83-20.57)	8.65 (6.87-10.84)	22.19 (17.42-28.16)	30.84 (24.28-39.00)
55-59 years	4.13 (3.27-5.17)	8.99 (7.05-11.42)	13.12 (10.33-16.59)	4.61 (3.67-5.76)	10.29 (8.08-13.04)	14.90 (11.75-18.80)	8.74 (6.94-10.93)	19.28 (15.14-24.45)	28.01 (22.07-35.38)
**Age group**	**Male**			**Female**			**Overall**		
	**HIC**	**LMIC**	**Worldwide**	**HIC**	**LMIC**	**Worldwide**	**HIC**	**LMIC**	**Worldwide**
60-64 years	3.84 (3.05-4.81)	7.42 (5.83-9.42)	11.27 (8.88-14.23)	4.45 (3.55-5.56)	8.74 (6.87-11.07)	13.20 (10.42-16.63)	8.30 (6.60-10.37)	16.17 (12.70-20.50)	24.46 (19.3-30.87)
65-69 years	3.43 (2.73-4.29)	6.25 (4.91-7.92)	9.68 (7.63-12.22)	4.18 (3.33-5.21)	7.62 (5.99-9.64)	11.80 (9.33-14.85)	7.61 (6.06-9.50)	13.87 (10.90-17.57)	21.48 (16.96-27.07)
Overall (5-69 years)	66.45 (53.27-82.26)	245.61 (194.54-308.28)	312.05 (247.81-390.53)	70.95 (57.07-87.50)	262.21 (208.18-328.20)	333.17 (265.25-415.70)	137.40 (110.34-169.75)	507.82 (402.72-636.47)	645.22 (513.06-806.23)

Table S8. Estimated numbers of people with current wheezing or ever asthma in World Bank income and World Health Organization combined regions in 2019, by age group

Age group	AFR	AMR	SEAR	EUR	EMR	WPR
			Current wheezing (million, 95% CI)			
5-9 years	20.38 (16.31-25.32)	7.57 (6.10-9.34)	20.05 (16.04-24.90)	6.85 (5.53-8.43)	8.76 (7.01-10.86)	14.67 (11.76-18.19)
10-14 years	19.00 (15.21-23.57)	8.13 (6.55-10.02)	22.16 (17.74-27.49)	7.02 (5.67-8.63)	8.27 (6.63-10.25)	15.03 (12.06-18.61)
15-19 years	15.72 (12.58-19.53)	7.94 (6.40-9.80)	21.48 (17.18-26.68)	6.34 (5.12-7.80)	7.23 (5.79-8.97)	14.12 (11.32-17.51)
20-24 years	13.03 (10.35-16.28)	7.88 (6.31-9.79)	20.24 (16.09-25.30)	6.30 (5.06-7.80)	6.66 (5.30-8.32)	14.42 (11.49-17.99)
25-29 years	10.97 (8.68-13.78)	7.87 (6.27-9.81)	18.87 (14.92-23.69)	6.92 (5.53-8.62)	6.47 (5.13-8.11)	15.57 (12.34-19.52)
30-34 years	9.35 (7.38-11.76)	7.47 (5.94-9.33)	17.71 (13.98-22.28)	7.64 (6.08-9.53)	6.16 (4.88-7.74)	18.66 (14.75-23.43)
35-39 years	7.87 (6.21-9.90)	7.05 (5.60-8.81)	16.64 (13.14-20.94)	7.57 (6.03-9.44)	5.41 (4.28-6.79)	15.38 (12.17-19.31)
40-44 years	6.38 (5.04-8.03)	6.49 (5.16-8.10)	14.80 (11.69-18.62)	7.36 (5.87-9.17)	4.51 (3.57-5.66)	14.64 (11.58-18.37)
45-49 years	5.12 (4.04-6.44)	6.03 (4.80-7.54)	13.24 (10.45-16.66)	7.23 (5.76-9.00)	3.67 (2.90-4.61)	17.22 (13.62-21.62)
50-54 years	4.05 (3.19-5.10)	5.74 (4.56-7.18)	11.61 (9.15-14.63)	7.07 (5.64-8.82)	3.04 (2.40-3.83)	17.18 (13.57-21.61)
55-59 years	3.22 (2.53-4.06)	5.50 (4.36-6.89)	9.84 (7.74-12.42)	7.09 (5.63-8.86)	2.46 (1.94-3.10)	14.12 (11.13-17.79)
60-64 years	2.48 (1.94-3.14)	4.84 (3.83-6.08)	7.97 (6.25-10.10)	6.42 (5.09-8.05)	1.91 (1.50-2.41)	11.35 (8.92-14.34)
65-69 years	1.82 (1.42-2.31)	4.01 (3.16-5.05)	5.96 (4.66-7.58)	5.55 (4.38-6.99)	1.36 (1.06-1.73)	10.52 (8.24-13.34)
Overall (569 years)	119.39 (94.88-149.24)	86.53 (69.05-107.74)	200.57 (159.02-251.29)	89.36 (71.39-111.14)	65.91 (52.39-82.37)	192.88 (152.94-241.64)
			Ever asthma (million, 95% CI)			
5-9 years	27.91 (22.45-34.38)	14.63 (11.90-17.79)	27.76 (22.33-34.20)	11.63 (9.48-14.11)	13.03 (10.5-16.02)	22.48 (18.15-27.58)
10-14 years	22.17 (17.77-27.44)	13.48 (10.92-16.49)	26.14 (20.94-32.35)	10.27 (8.33-12.51)	10.50 (8.42-12.97)	19.69 (15.83-24.28)
15-19 years	13.62 (10.80-17.06)	9.90 (7.91-12.28)	18.79 (14.90-23.54)	7.02 (5.63-8.69)	6.81 (5.41-8.52)	13.86 (11.03-17.31)
20-24 years	9.36 (7.39-11.80)	8.23 (6.54-10.28)	14.69 (11.60-18.52)	5.86 (4.67-7.30)	5.22 (4.12-6.56)	11.85 (9.38-14.89)
25-29 years	6.97 (5.49-8.82)	7.34 (5.81-9.20)	12.11 (9.53-15.31)	5.7 (4.52-7.14)	4.48 (3.53-5.66)	11.31 (8.92-14.26)
30-34 years	5.43 (4.27-6.88)	6.39 (5.05-8.03)	10.40 (8.17-13.18)	5.74 (4.55-7.21)	3.91 (3.08-4.95)	12.27 (9.66-15.51)
35-39 years	4.32 (3.39-5.48)	5.69 (4.50-7.17)	9.23 (7.24-11.71)	5.41 (4.28-6.81)	3.25 (2.55-4.11)	9.80 (7.71-12.40)

40-44 years	3.40 (2.67-4.32)	5.11 (4.04-6.44)	7.97 (6.26-10.12)	5.15 (4.07-6.48)	2.63 (2.07-3.33)	9.15 (7.20-11.58)
45-49 years	2.72 (2.13-3.45)	4.76 (3.76-6.00)	7.11 (5.58-9.03)	5.07 (4.01-6.37)	2.13 (1.67-2.7)	10.65 (8.38-13.48)
50-54 years	2.19 (1.72-2.78)	4.65 (3.67-5.85)	6.34 (4.98-8.05)	5.08 (4.02-6.39)	1.79 (1.41-2.27)	10.74 (8.45-13.60)

Age group	AFR	AMR	SEAR	EUR	EMR	WPR
55-59 years	1.8 (1.41-2.28)	4.65 (3.68-5.85)	5.55 (4.36-7.04)	5.23 (4.14-6.57)	1.5 (1.18-1.89)	9.24 (7.28-11.69)
60-64 years	1.44 (1.13-1.83)	4.31 (3.41-5.41)	4.69 (3.68-5.94)	4.94 (3.91-6.20)	1.2 (0.95-1.53)	7.87 (6.2-9.94)
65-69 years	1.11 (0.87-1.40)	3.74 (2.97-4.69)	3.66 (2.88-4.64)	4.47 (3.55-5.61)	0.9 (0.71-1.14)	7.64 (6.02-9.64)
Overall (569 years)	102.44 (81.49-127.91)	92.88 (74.16-115.5)	154.45 (122.45-193.64)	81.57 (65.16-101.39)	57.34 (45.59-71.65)	156.55 (124.2-196.14)

Table S9. Estimated prevalence and number of people aged 0-79 years living with current wheezing or ever asthma in 201 countries and territories in 2019

Country	Current wheezing		Ever asthma	
	Number of cases (thousand, 95% CI)	Prevalence (%)	Number of cases (thousand, 95% CI)	Prevalence (%)
Antigua and Barbuda	9.13 (7.34-11.27)	10.85 (8.73-13.40)	3.14 (2.53-3.87)	3.74 (3.01-4.61)
Aruba	9.81 (7.89-12.13)	10.82 (8.70-13.37)	12.66 (10.17-15.63)	13.95 (11.21-17.23)
Bahamas	32.21 (25.94-39.75)	9.36 (7.53-11.55)	52.36 (42.14-64.53)	15.21 (12.24-18.75)
Barbados	20.75 (16.69-25.65)	8.69 (6.99-10.73)	7.99 (6.42-9.86)	3.34 (2.69-4.13)
Curaçao	14.66 (11.78-18.12)	10.86 (8.73-13.42)	20.56 (16.53-25.36)	15.23 (12.25-18.79)
Guadeloupe	35.13 (28.25-43.41)	10.87 (8.74-13.43)	50.33 (40.48-62.07)	15.58 (12.53-19.21)
Martinique	32.48 (26.10-40.16)	10.84 (8.71-13.40)	44.73 (35.93-55.24)	14.92 (11.99-18.43)
Puerto Rico	259.90 (208.94-321.24)	10.81 (8.69-13.36)	304.32 (244.66-375.48)	12.66 (10.18-15.62)
Trinidad and Tobago	130.19 (104.72-160.83)	10.79 (8.68-13.34)	69.99 (56.29-86.32)	5.8 (4.67-7.16)
United States Virgin Islands	9.07 (7.30-11.21)	10.84 (8.72-13.39)	13.09 (10.53-16.13)	15.64 (12.58-19.27)
Argentina	4,220.30 (3,399.07-5,207.02)	11.22 (9.03-13.84)	621.98 (501.08-765.54)	1.65 (1.33-2.03)
Chile	2,501.78 (2,012.75-3,090.04)	15.37 (12.36-18.98)	1,222.74 (983.20-1,508.36)	7.51 (6.04-9.27)
French Guiana	27.00 (21.77-33.29)	10.92 (8.81-13.46)	41.29 (33.33-50.69)	16.7 (13.48-20.50)
Uruguay	320.00 (257.52-395.14)	11.22 (9.03-13.86)	14.16 (11.39-17.46)	0.50 (0.40-0.61)
Canada	2,973.83 (2,389.96-3,676.91)	9.66 (7.76-11.95)	5,508.26 (4,422.56-6,806.78)	17.9 (14.37-22.11)
United States of America	29,757.10 (23,931.59-36,767.14)	10.92 (8.79-13.50)	40,956.46 (32,927.11-50,533.52)	15.03 (12.09-18.55)
Cyprus	159.93 (128.66-197.54)	15.74 (12.66-19.44)	138.73 (111.40-171.42)	13.65 (10.96-16.87)
Israel	924.55 (745.02-1,140.1)	13.25 (10.68-16.34)	798.09 (643.63-981.13)	11.44 (9.23-14.06)
Czechia	1,220.59 (980.84-1,509.28)	14.11 (11.34-17.44)	1,113.78 (893.81-1,377.16)	12.87 (10.33-15.92)
Hungary	1,099.25 (883.22-1,359.42)	13.85 (11.13-17.13)	689.36 (552.94-852.85)	8.69 (6.97-10.75)
Poland	4,153.21 (3,337.10-5,136.02)	13.25 (10.65-16.39)	2,951.86 (2,368.28-3,650.93)	9.42 (7.56-11.65)
Slovakia	664.72 (534.18-821.90)	14.47 (11.63-17.89)	473.91 (380.22-586.15)	10.32 (8.28-12.76)
Channel Islands	19.95 (16.03-24.66)	14.05 (11.29-17.37)	17.10 (13.72-21.15)	12.04 (9.66-14.89)
Denmark	534.56 (429.77-660.69)	11.60 (9.33-14.34)	839.76 (674.43-1,037.38)	18.23 (14.64-22.52)
Estonia	147.90 (118.87-182.85)	13.83 (11.11-17.10)	137.47 (110.38-169.86)	12.85 (10.32-15.88)

Finland	494.95 (397.84-611.87)	11.32 (9.10-13.99)	655.44 (526.44-809.61)	14.99 (12.04-18.51)

Country	Current wheezing		Ever asthma	
	Number of cases (thousand, 95% CI)	Prevalence (%)	Number of cases (thousand, 95% CI)	Prevalence (%)
Iceland	29.45 (23.69-36.38)	10.42 (8.38-12.87)	47.23 (37.98-58.26)	16.71 (13.43-20.61)
Ireland	514.57 (414.03-635.49)	12.59 (10.13-15.55)	663.28 (533.60-817.76)	16.22 (13.05-20)
Latvia	231.59 (186.10-286.35)	15.26 (12.26-18.87)	173.98 (139.62-215.12)	11.46 (9.20-14.18)
Lithuania	286.98 (230.60-354.89)	12.98 (10.43-16.05)	294.74 (236.54-364.41)	13.33 (10.70-16.48)
Norway	505.51 (406.51-624.64)	11.47 (9.22-14.17)	896.24 (719.99-1,106.81)	20.33 (16.33-25.11)
Sweden	878.33 (706.21-1,085.49)	11.09 (8.92-13.71)	1,315.69 (1,057.14-1,624.46)	16.61 (13.35-20.51)
United Kingdom	7,659.82 (6,159.39-9,465.51)	14.09 (11.33-17.41)	7,333.47 (5,892.47-9,054.36)	13.49 (10.84-16.65)
Croatia	502.87 (404.02-621.93)	15.04 (12.08-18.6)	300.46 (241.09-371.57)	8.99 (7.21-11.11)
Greece	1,320.80 (1,061.43-1,633.08)	15.90 (12.78-19.66)	774.83 (621.61-958.39)	9.33 (7.48-11.54)
Italy	5,834.37 (4,687.11-7,216.27)	12.25 (9.84-15.16)	4,452.15 (3,570.52-5,509.13)	9.35 (7.50-11.57)
Malta	44.56 (35.81-55.11)	12.66 (10.17-15.65)	34.95 (28.04-43.24)	9.93 (7.96-12.28)
Portugal	1,027.72 (825.74-1,270.97)	12.67 (10.18-15.66)	334.75 (268.47-414.21)	4.13 (3.31-5.11)
Slovenia	200.98 (161.46-248.59)	11.93 (9.58-14.76)	212.21 (170.27-262.45)	12.6 (10.11-15.58)
Spain	4,922.36 (3,955.48-6,086.72)	13.03 (10.47-16.11)	2,500.16 (2,005.88-3,092.29)	6.62 (5.31-8.18)
Austria	966.92 (776.97-1,195.66)	13.37 (10.74-16.53)	984.54 (789.72-1218.00)	13.61 (10.92-16.84)
Belgium	1,178.35 (947.34-1,456.40)	12.65 (10.17-15.64)	1,390.48 (1,116.95-1,717.32)	14.93 (11.99-18.44)
France	7,424.13 (5,969.67-9,174.56)	14.34 (11.53-17.72)	6,915.35 (5,558-8,535.42)	13.36 (10.74-16.49)
Germany	8,560.49 (6,877.24-10,588.01)	12.95 (10.40-16.01)	12,089.65 (9,696.24-14,958.61)	18.28 (14.66-22.62)
Luxembourg	63.10 (50.72-77.99)	12.11 (9.73-14.97)	95.70 (76.79-118.36)	18.37 (14.74-22.71)
Netherlands	1,726.23 (1,387.58-2,133.92)	12.5 (10.05-15.45)	2,434.39 (1,954.59-3,008.23)	17.63 (14.15-21.78)
Switzerland	879.56 (706.82-1,087.56)	12.68 (10.19-15.68)	1,463.27 (1,173.95-1,809.85)	21.09 (16.92-26.09)
Bahrain	198.04 (159.41-244.45)	13.08 (10.53-16.15)	85.62 (68.73-105.84)	5.66 (4.54-6.99)
Kuwait	513.06 (413.00-633.25)	13.28 (10.69-16.39)	462.30 (371.55-570.67)	11.97 (9.62-14.77)
Oman	448.07 (360.82-552.84)	10.04 (8.09-12.39)	334.71 (268.99-413.17)	7.50 (6.03-9.26)
Qatar	314.42 (253.03-388.16)	11.74 (9.45-14.49)	265.60 (212.73-329.16)	9.91 (7.94-12.29)
Saudi Arabia	3,430.72 (2,763.67-4,231.63)	11.18 (9.01-13.79)	2,835.21 (2,282.20-3,493.28)	9.24 (7.44-11.38)

Country	Current wheezing		Ever asthma	
	Number of cases (thousand, 95% CI)	Prevalence (%)	Number of cases (thousand, 95% CI)	Prevalence (%)
State of Palestine	507.17 (409.68-623.93)	12.05 (9.73-14.82)	434.81 (351.87-532.36)	10.33 (8.36-12.64)
United Arab Emirates	1,142.90 (919.75-1,410.99)	12.41 (9.99-15.32)	1,180.48 (946.05-1,461.95)	12.82 (10.27-15.87)
China, Hong Kong SAR	776.13 (623.39-960.08)	12.57 (10.1-15.55)	969.75 (776.71-1201.76)	15.71 (12.58-19.46)
China, Macao SAR	71.88 (57.75-88.88)	12.80 (10.28-15.82)	89.01 (71.32-110.25)	15.85 (12.7-19.63)
China, Taiwan Province of China	2,654.01 (2,133.09-3,280.85)	13.01 (10.46-16.08)	3,150.75 (2,524.88-3,902.21)	15.45 (12.38-19.13)
Japan	11,341.30 (9,114.00-14,022.33)	12.02 (9.66-14.86)	15,220.2 (12,207.53-18,831.26)	16.13 (12.93-19.95)
Republic of Korea	5,605.77 (4,505.54-6,929.77)	12.77 (10.27-15.79)	6,807.92 (5,456.36-8,430.27)	15.51 (12.43-19.21)
Brunei Darussalam	44.77 (36.06-55.23)	11.51 (9.27-14.20)	64.44 (51.85-79.44)	16.57 (13.33-20.42)
Singapore	577.07 (463.62-713.65)	11.27 (9.05-13.93)	755.21 (604.76-936.09)	14.74 (11.81-18.27)
Australia	2,042.65 (1,643.20-2,523.12)	9.88 (7.95-12.20)	2,696.05 (2,167.44-3,326.63)	13.04 (10.48-16.09)
New Zealand	389.32 (313.20-480.86)	9.88 (7.95-12.21)	523.60 (421.06-645.84)	13.29 (10.69-16.40)
New Caledonia	32.38 (26.08-39.94)	13.19 (10.62-16.26)	41.35 (33.27-50.95)	16.84 (13.55-20.75)
Guam	18.90 (15.23-23.31)	13.25 (10.68-16.34)	15.82 (12.74-19.48)	11.09 (8.93-13.66)
Burundi	1,357.82 (1,079.39-1,696.80)	14.43 (11.47-18.03)	1,236.02 (983.65-1,542.57)	13.14 (10.45-16.40)
Comoros	102.19 (81.17-127.79)	14.27 (11.34-17.85)	90.97 (72.31-113.70)	12.70 (10.10-15.88)
Eritrea	394.63 (313.59-493.32)	13.55 (10.77-16.93)	389.53 (309.83-486.45)	13.37 (10.64-16.70)
Ethiopia	12,596.34 (10,009.66-15,746.94)	13.49 (10.72-16.87)	11,723.08 (9,321.54-14,645.66)	12.56 (9.98-15.69)
Kenya	6,212.09 (4,935.81-7,766.74)	13.80 (10.96-17.25)	5,591.49 (4,444.97-6,987.52)	12.42 (9.87-15.52)
Madagascar	3,487.12 (2,770.90-4,359.40)	15.48 (12.3-19.35)	3,120.46 (2,480.87-3,899.06)	13.85 (11.01-17.31)
Malawi	2,217.43 (1,762.89-2,770.78)	14.30 (11.37-17.87)	2,009.52 (1,598.85-2,508.60)	12.96 (10.31-16.18)
Mauritius	97.11 (76.85-121.88)	8.75 (6.93-10.99)	87.42 (69.14-109.93)	7.88 (6.23-9.91)
Mayotte	30.34 (24.10-37.93)	13.54 (10.76-16.93)	29.40 (23.38-36.74)	13.12 (10.43-16.40)
Mozambique	3,612.44 (2,872.03-4,513.77)	14.52 (11.55-18.15)	3,497.35 (2,782.93-4,365.35)	14.06 (11.19-17.55)
Réunion	100.14 (79.30-125.62)	13.32 (10.55-16.71)	99.34 (78.69-124.69)	13.21 (10.47-16.58)
Rwanda	1,528.14 (1,213.89-1,911.03)	14.44 (11.47-18.05)	1,320.00 (1,049.33-1,649.57)	12.47 (9.91-15.58)
Seychelles	7.27 (5.76-9.12)	8.56 (6.78-10.73)	7.37 (5.84-9.25)	8.67 (6.87-10.88)

Country	Current wheezing		Ever asthma	
	Number of cases (thousand, 95% CI)	Prevalence (%)	Number of cases (thousand, 95% CI)	Prevalence (%)
South Sudan	1,359.67 (1,080.56-1,699.57)	14.85 (11.80-18.57)	1,198.61 (953.25-1,497.09)	13.09 (10.41-16.35)
Uganda	5,159.79 (4,103.5-6,445.28)	14.22 (11.31-17.77)	4,902.04 (3,902.20-6,115.75)	13.51 (10.76-16.86)
United Republic of Tanzania	6,329.01 (5,030.79-7,909.69)	13.26 (10.54-16.58)	6,160.42 (4,901.18-7,690.97)	12.91 (10.27-16.12)
Zambia	2,045.30 (1,626.30-2,555.28)	13.82 (10.99-17.27)	2,015.66 (1,603.94-2,515.85)	13.62 (10.84-17.00)
Zimbabwe	1,577.03 (1,253.52-1,970.96)	12.79 (10.17-15.98)	1,530.98 (1,218.03-1,911.37)	12.41 (9.88-15.50)
Angola	2,993.75 (2,380.58-3,740.02)	11.59 (9.21-14.47)	3,477.04 (2,767.77-4,338.10)	13.46 (10.71-16.79)
Cameroon	2,708.09 (2,152.25-3,384.99)	12.60 (10.02-15.75)	2,861.41 (2,275.95-3,573.42)	13.32 (10.59-16.63)
Central African Republic	588.18 (467.74-734.76)	14.90 (11.85-18.61)	576.98 (459.14-720.12)	14.61 (11.63-18.24)
Chad	1,844.82 (1,467.16-2,304.4)	14.31 (11.38-17.87)	1,773.38 (1,411.7-2,212.41)	13.75 (10.95-17.16)
Congo	565.36 (449.23-706.81)	12.56 (9.98-15.71)	611.11 (486-763.33)	13.58 (10.8-16.96)
Democratic Republic of the Congo	10,258.98 (8,156.54-12,818.17)	14.66 (11.66-18.32)	10,018.30 (7,973.71-12,501.19)	14.32 (11.4-17.87)
Equatorial Guinea	151.49 (120.30-189.50)	13.22 (10.49-16.53)	149.19 (118.5-186.64)	13.02 (10.34-16.28)
Gabon	159.30 (126.47-199.33)	8.78 (6.97-10.99)	207.28 (164.67-259.25)	11.42 (9.08-14.29)
Sao Tome and Principe	22.91 (18.21-28.64)	12.71 (10.10-15.89)	25.75 (20.49-32.16)	14.28 (11.36-17.84)
Botswana	230.21 (182.72-288.13)	11.63 (9.23-14.56)	237.21 (188.34-296.86)	11.99 (9.52-15.00)
Eswatini	127.82 (101.55-159.82)	13.08 (10.39-16.35)	114.67 (91.15-143.32)	11.73 (9.33-14.66)
Lesotho	242.04 (192.12-302.90)	13.37 (10.61-16.73)	203.18 (161.27-254.39)	11.22 (8.91-14.05)
Namibia	251.01 (199.33-314.01)	11.88 (9.44-14.87)	249.32 (198.10-311.75)	11.80 (9.38-14.76)
South Africa	4,869.11 (3,861.24-6,099.23)	9.55 (7.57-11.96)	5,189.59 (4,116.14-6,503.29)	10.17 (8.07-12.75)
Benin	1,340.88 (1,065.57-1,676.19)	13.79 (10.96-17.24)	1,349.57 (1,073.40-1,685.46)	13.88 (11.04-17.34)
Burkina Faso	2,361.05 (1,877.14-2,950.09)	14.15 (11.25-17.68)	2,273.09 (1,808.73-2,837.31)	13.62 (10.84-17)
Cabo Verde	45.30 (35.93-56.75)	9.37 (7.43-11.74)	50.83 (40.31-63.71)	10.52 (8.34-13.18)
Côte d'Ivoire	2,743.77 (2,180.57-3,429.61)	12.88 (10.23-16.1)	2,811.06 (2,235.54-3,511.22)	13.19 (10.49-16.48)
Gambia	281.84 (224.05-352.20)	14.70 (11.69-18.37)	285.59 (227.22-356.54)	14.90 (11.85-18.6)
Ghana	3,085.53 (2,450.15-3,860.00)	11.95 (9.49-14.95)	3,190.98 (2,535.51-3,989.95)	12.36 (9.82-15.46)
Guinea	1,558.08 (1,238.64-1,946.97)	14.79 (11.76-18.48)	1,484.50 (1,181.02-1,853.38)	14.09 (11.21-17.59)

Country	Current wheezing		Ever asthma	
	Number of cases (thousand, 95% CI)	Prevalence (%)	Number of cases (thousand, 95% CI)	Prevalence (%)
Guinea-Bissau	235.62 (187.25-294.54)	14.77 (11.73-18.46)	225.22 (179.13-281.28)	14.11 (11.23-17.63)
Liberia	587.27 (466.63-734.23)	14.24 (11.32-17.81)	580.85 (461.86-725.64)	14.09 (11.20-17.60)
Mali	2,348.81 (1,868.04-2,933.84)	14.77 (11.75-18.45)	2,359.42 (1,878.37-2,943.25)	14.84 (11.81-18.51)
Mauritania	446.43 (354.64-558.26)	11.83 (9.40-14.79)	466.11 (370.53-582.49)	12.35 (9.82-15.44)
Niger	2,606.78 (2,073.59-3,255.48)	14.18 (11.28-17.71)	2,538.88 (2,022.1-3,165.47)	13.81 (11.00-17.22)
Nigeria	21,888.51 (17,396.47-27,358.82)	13.26 (10.54-16.57)	22,382.08 (17,806.27-27,944.38)	13.56 (10.79-16.93)
Senegal	1,714.85 (1,362.86-2,143.52)	12.73 (10.12-15.91)	1,785.64 (1,420.49-2,229.58)	13.26 (10.54-16.55)
Sierra Leone	1,004.39 (798.15-1,255.57)	15.33 (12.18-19.16)	928.14 (738.02-1,159.50)	14.17 (11.26-17.70)
Togo	918.84 (730.07-1,148.78)	13.58 (10.79-16.97)	896.87 (713.18-1,120.40)	13.25 (10.54-16.55)

Country	Current wheezing		Ever asthma	
	Number of cases (thousand, 95% CI)	Prevalence (%)	Number of cases (thousand, 95% CI)	Prevalence (%)
Algeria	2,923.38 (2,317.66-3,662.84)	8.05 (6.38-10.08)	3,546.38 (2,813.30-4,443.24)	9.76 (7.74-12.23)
Western Sahara	69.53 (55.12-87.11)	13.42 (10.64-16.81)	67.76 (53.72-84.98)	13.08 (10.37-16.40)
Cuba	969.28 (766.57-1,217.40)	10.18 (8.05-12.78)	753.44 (595.73-947.77)	7.91 (6.26-9.95)
Dominican Republic	827.57 (656.12-1,036.90)	8.96 (7.11-11.23)	798.49 (633.27-1,000.74)	8.65 (6.86-10.84)
Grenada	8.94 (7.08-11.21)	9.32 (7.38-11.69)	6.80 (5.39-8.54)	7.09 (5.62-8.90)
Haiti	1,259.15 (999.31-1,576.08)	13.03 (10.34-16.3)	1,045.92 (830.44-1,309.02)	10.82 (8.59-13.54)
Jamaica	235.88 (186.89-295.73)	9.28 (7.35-11.64)	195.36 (154.78-245.13)	7.69 (6.09-9.65)
Saint Lucia	14.24 (11.27-17.86)	8.94 (7.08-11.22)	9.78 (7.74-12.30)	6.14 (4.86-7.72)
Saint Vincent and the Grenadines	8.68 (6.88-10.89)	9.06 (7.18-11.37)	7.10 (5.62-8.91)	7.40 (5.86-9.29)
Belize	32.98 (26.17-41.30)	9.72 (7.71-12.16)	27.45 (21.78-34.39)	8.09 (6.42-10.13)
Costa Rica	376.32 (297.98-472.10)	8.63 (6.83-10.82)	340.32 (269.44-427.40)	7.80 (6.18-9.80)
El Salvador	491.98 (390.08-616.38)	8.93 (7.08-11.19)	463.66 (367.68-581.15)	8.42 (6.68-10.55)
Guatemala	1,734.00 (13,76.68-2,169.65)	11.56 (9.18-14.46)	1,459.61 (1,159.17-1,826.24)	9.73 (7.73-12.18)
Honduras	930.09 (738.14-1,164.20)	11.01 (8.74-13.78)	795.87 (631.66-996.53)	9.42 (7.48-11.80)
Mexico	10,458.76 (8,290.24-13,107.03)	9.46 (7.50-11.85)	9,536.92 (7,560.55-11,958.10)	8.62 (6.84-10.81)
Nicaragua	647.07 (513.22-810.41)	11.40 (9.04-14.28)	537.29 (426.28-673.04)	9.47 (7.51-11.86)
Panama	317.60 (251.74-398.04)	8.77 (6.95-10.99)	288.93 (229.08-362.22)	7.98 (6.32-10.00)
Bolivia (Plurinational State of)	943.84 (748.86-1,181.73)	9.67 (7.67-12.11)	861.25 (683.56-1078.39)	8.82 (7.00-11.05)
Brazil	16,200.03 (12,828.26-20,322.03)	8.81 (6.98-11.06)	14,822.16 (11,734.61-18,615.77)	8.06 (6.38-10.13)
Colombia	3,781.47 (2,995.64-4,741.76)	8.64 (6.84-10.83)	3,520.98 (2,788.81-4,419.68)	8.04 (6.37-10.10)
Ecuador	1,360.13 (1,078.40-1,704.09)	9.13 (7.24-11.44)	1,202.46 (953.58-1,507.14)	8.07 (6.4-10.12)
Guyana	65.83 (52.20-82.46)	9.74 (7.73-12.2)	50.62 (40.15-63.43)	7.49 (5.94-9.39)
Paraguay	617.79 (490.03-773.69)	10.20 (8.09-12.78)	531.53 (421.68-665.90)	8.78 (6.96-11.00)
Peru	2,619.08 (2,075.10-3,283.69)	9.39 (7.44-11.77)	2,343.69 (1,857.15-2,940.35)	8.40 (6.66-10.54)
Suriname	46.07 (36.52-57.73)	9.15 (7.25-11.47)	40.63 (32.21-50.93)	8.07 (6.40-10.12)
Venezuela (Bolivarian Republic of)	2,227.91 (1,766.10-,2791.86)	9.00 (7.14-11.28)	2,157.37 (1,711.16-2,703.42)	8.72 (6.92-10.93)
Bangladesh	19,266.07 (15,281.52-24,127.90)	13.46 (10.67-16.85)	14,302.13 (11,340.35-17,929.00)	9.99 (7.92-12.52)

Country	Current wheezing		Ever asthma	
	Number of cases (thousand, 95% CI)	Prevalence (%)	Number of cases (thousand, 95% CI)	Prevalence (%)
Bhutan	66.74 (52.92-83.59)	9.99 (7.92-12.51)	56.48 (44.77-70.85)	8.45 (6.70-10.60)
India	134,560.31 (106,691.80-168,578.76)	11.22 (8.90-14.06)	105,727.37 (83,817.84-132,567.25)	8.82 (6.99-11.06)
Maldives	42.37 (33.58-53.12)	8.76 (6.95-10.99)	34.46 (27.26-43.32)	7.13 (5.64-8.96)
Nepal	3,234.17 (2,566.37-4,048.81)	12.96 (10.29-16.23)	2,428.48 (1,926.97-3,041.59)	9.73 (7.72-12.19)
Sri Lanka	2,252.07 (1,783.85-2,824.26)	12.39 (9.82-15.54)	1,547.57 (1,226.23-1,941.69)	8.52 (6.75-10.69)
Dem. People's Republic of Korea	2,529.46 (2,002.80-3,173.27)	11.40 (9.03-14.30)	2,000.09 (1,582.91-2,513.07)	9.02 (7.14-11.33)
Indonesia	26,111.82 (20,701.16-32,716.52)	11.00 (8.72-13.78)	21,045.78 (16,681.51-26,394.47)	8.87 (7.03-11.12)
Myanmar	6,269.54 (4,970.17-7,856.05)	13.13 (10.41-16.45)	4,536.62 (3,595.94-5,689.37)	9.50 (7.53-11.91)
Thailand	6,064.30 (4,797.69-7,613.92)	10.07 (7.96-12.64)	4,660.74 (3,685.53-5,862.01)	7.74 (6.12-9.73)
Timor-Leste	168.88 (134.18-211.16)	15.59 (12.38-19.49)	124.17 (98.67-155.26)	11.46 (9.11-14.33)
Armenia	239.40 (189.48-300.45)	9.45 (7.48-11.85)	160.67 (127.18-201.82)	6.34 (5.02-7.96)
Azerbaijan	804.93 (637.48-1,009.59)	9.09 (7.20-11.40)	549.93 (435.54-690.37)	6.21 (4.92-7.79)
Georgia	348.08 (275.44-436.93)	10.46 (8.28-13.13)	219.59 (173.82-275.83)	6.60 (5.22-8.29)
Turkey	7,129.77 (5,649.62-8,937.89)	9.88 (7.83-12.39)	5,021.33 (3,979.07-6,299.38)	6.96 (5.51-8.73)
Kazakhstan	1,489.44 (1,180.22-1,867.16)	9.42 (7.47-11.81)	1,041.85 (826.33-1,305.65)	6.59 (5.23-8.26)
Kyrgyzstan	586.19 (465.00-734.07)	10.64 (8.44-13.33)	384.42 (305.15-481.26)	6.98 (5.54-8.74)
Tajikistan	820.95 (651.70-1,027.33)	10.47 (8.31-13.10)	548.11 (435.47-685.45)	6.99 (5.55-8.74)
Turkmenistan	486.13 (385.51-608.95)	9.46 (7.50-11.85)	343.44 (272.53-430.14)	6.68 (5.30-8.37)
Uzbekistan	2,653.96 (2,104.07-3,325.36)	9.22 (7.31-11.55)	1,892.73 (1,501.04-2,372.21)	6.57 (5.21-8.24)
Belarus	747.80 (591.21-939.54)	9.34 (7.38-11.74)	507.23 (401.12-637.93)	6.34 (5.01-7.97)
Bulgaria	582.54 (460.43-732.09)	10.33 (8.17-12.99)	363.75 (287.53-457.71)	6.45 (5.10-8.12)
Republic of Moldova	346.66 (274.13-435.45)	9.73 (7.69-12.22)	203.02 (160.48-255.46)	5.70 (4.50-7.17)
Romania	1,660.09 (1,312.53-2,085.62)	10.41 (8.23-13.08)	987.04 (780.37-1,241.73)	6.19 (4.90-7.79)
Russian Federation	12,082.34 (9,553.96-15,177.64)	9.88 (7.81-12.41)	7,942.89 (6,283.04-9,986.17)	6.49 (5.14-8.16)
Ukraine	3,494.12 (2,762.51-4,389.87)	9.44 (7.46-11.86)	2,282.30 (1,804.55-2,870.96)	6.17 (4.87-7.76)

Country	Current wheezing		Ever asthma	
	Number of cases (thousand, 95% CI)	Prevalence (%)	Number of cases (thousand, 95% CI)	Prevalence (%)
Albania	258.23 (204.36-324.11)	10.61 (8.40-13.32)	161.01 (127.37-202.42)	6.61 (5.23-8.32)
Bosnia and Herzegovina	358.00 (283.00-449.86)	12.80 (10.12-16.08)	192.57 (152.22-242.31)	6.88 (5.44-8.66)
Montenegro	61.29 (48.49-76.95)	11.58 (9.16-14.54)	37.22 (29.45-46.78)	7.03 (5.57-8.84)
North Macedonia	175.48 (138.79-220.38)	9.83 (7.78-12.35)	108.63 (85.89-136.64)	6.09 (4.81-7.66)
Serbia	857.23 (677.82-1076.89)	11.79 (9.32-14.81)	486.23 (384.45-611.62)	6.69 (5.29-8.41)
Djibouti	121.58 (96.44-152.24)	14.33 (11.37-17.95)	104.10 (82.56-130.45)	12.27 (9.73-15.38)
Somalia	1,827.27 (1,453.17-2,282.49)	14.67 (11.67-18.32)	1,724.45 (1,372.64-2,151.56)	13.84 (11.02-17.27)
Egypt	7,879.56 (6,252.45-9,864.08)	9.30 (7.38-11.64)	7,320.26 (5,812.31-9,161.38)	8.64 (6.86-10.81)
Libya	644.56 (511.09-807.46)	10.79 (8.55-13.51)	601.46 (476.93-753.96)	10.07 (7.98-12.62)
Morocco	2,766.99 (2,192.91-3,468.12)	8.76 (6.95-10.98)	2,776.05 (2,200.93-3,480.52)	8.79 (6.97-11.02)
Sudan	4,268.90 (3,391.90-5,337.08)	11.95 (9.49-14.94)	3,809.51 (3,028.85-4,759.75)	10.66 (8.48-13.32)
Tunisia	937.46 (742.50-1,175.72)	9.30 (7.36-11.66)	874.21 (692.55-1,097.14)	8.67 (6.87-10.88)
Iraq	3,047.51 (2,420.65-3,811.31)	9.14 (7.26-11.43)	3,290.46 (2,615.04-4,113.39)	9.87 (7.85-12.34)
Jordan	809.25 (642.43-1,012.61)	9.19 (7.29-11.5)	892.14 (708.43-1,116.38)	10.13 (8.04-12.68)
Lebanon	701.94 (556.30-879.81)	11.76 (9.32-14.74)	628.89 (498.43-788.80)	10.53 (8.35-13.21)
Syrian Arab Republic	1,350.63 (1,071.50-1,691.15)	9.17 (7.28-11.49)	1,321.10 (1,048.19-1,654.84)	8.97 (7.12-11.24)
Yemen	2,736.49 (2,174.13-3,421.53)	11.09 (8.81-13.87)	2,572.52 (2,045.01-3,214.85)	10.43 (8.29-13.03)
Afghanistan	4,088.76 (3,250.73-5,108.80)	12.79 (10.17-15.99)	3,614.11 (2,874.88-4,512.92)	11.31 (9.00-14.12)
Iran (Islamic Republic of)	6,176.24 (4,891.90-7,745.82)	8.56 (6.78-10.73)	6,336.54 (5,019.16-7,953.51)	8.78 (6.95-11.02)
Pakistan	21,999.25 (17,465.24-27,526.45)	11.99 (9.52-15.01)	19,131.56 (15,194.1-23,936.22)	10.43 (8.28-13.05)
China	141,265.12 (111,759.24-177,360.43)	11.28 (8.93-14.17)	93,753.42 (74,139.29-117,913.23)	7.49 (5.92-9.42)
Mongolia	336.35 (266.69-421.39)	12.11 (9.60-15.17)	251.04 (199.16-314.51)	9.04 (7.17-11.32)
Cambodia	1,852.55 (1,469.67-2,319.69)	12.96 (10.28-16.22)	1,242.88 (986.32-1,556.50)	8.69 (6.90-10.89)
Lao People's Democratic Republic	912.43 (724.19-1,141.96)	14.68 (11.65-18.38)	590.27 (468.54-738.98)	9.50 (7.54-11.89)
Malaysia	2,563.28 (2,031.39-3,212.85)	9.16 (7.26-11.49)	2,101.81 (1,664.88-2,638.02)	7.51 (5.95-9.43)

Country	Current wheezing		Ever asthma	
	Number of cases (thousand, 95% CI)	Prevalence (%)	Number of cases (thousand, 95% CI)	Prevalence (%)
Philippines	11,537.20 (9,153.92-14,444.37)	12.25 (9.72-15.34)	8,206.83 (6,512.75-10,277.55)	8.71 (6.92-10.91)
Viet Nam	9,441.48 (7,478.34-11,840.28)	11.24 (8.90-14.09)	6,128.99 (4,853.68-7,695.03)	7.29 (5.78-9.16)
Fiji	103.96 (82.43-130.23)	13.46 (10.67-16.86)	71.29 (56.55-89.33)	9.23 (7.32-11.57)
Papua New Guinea	11,06.39 (878.54-1,384.10)	14.69 (11.66-18.37)	708.41 (562.67-886.22)	9.40 (7.47-11.76)
Solomon Islands	86.97 (69.10-108.74)	15.69 (12.46-19.61)	57.57 (45.77-71.93)	10.38 (8.26-12.97)
Vanuatu	36.12 (28.69-45.18)	14.29 (11.35-17.88)	24.64 (19.58-30.8)	9.75 (7.75-12.19)
Kiribati	16.87 (13.39-21.11)	16.89 (13.41-21.13)	11.17 (8.87-13.97)	11.19 (8.89-13.99)

Country	Current wheezing		Ever asthma	
	Number of cases (thousand, 95% CI)	Prevalence (%)	Number of cases (thousand, 95% CI)	Prevalence (%)
Micronesia (Fed. States of)	11.38 (9.03-14.24)	11.44 (9.08-14.32)	7.59 (6.03-9.51)	7.63 (6.06-9.56)
French Polynesia	27.68 (21.92-34.71)	11.33 (8.97-14.21)	19.43 (15.38-24.39)	7.95 (6.30-9.99)
Samoa	21.87 (17.37-27.37)	13.30 (10.56-16.64)	14.30 (11.36-17.88)	8.70 (6.91-10.87)
Tonga	11.12 (8.83-13.91)	12.55 (9.97-15.70)	7.49 (5.95-9.37)	8.45 (6.72-10.57)

Table S10. Pooled odds ratios of associated factors of current wheezing and ever asthma by World Bank region

Factor	WB region	Current wheezing				Ever asthma			
		Data points	Odds ratio	Lower CI	Upper CI	Data points	Odds ratio	Lower CI	Upper CI
**Age <7**	Global	6	0.773	0.613	0.975	11	1.078	0.927	1.254
	HIC	3	0.980	0.897	1.071	8	1.071	0.916	1.251
	LMIC	3	0.630	0.425	0.931	3	1.210	0.754	1.941
**Age 8-14**	Global	6	0.973	0.877	1.080	6	1.110	0.987	1.248
	HIC	3	0.930	0.896	0.965	3	1.056	0.963	1.158
	LMIC	3	1.025	0.810	1.296	3	1.411	0.875	2.276
**Age 15+**	Global	3	0.960	0.865	1.066	9	1.341	1.109	1.623
	HIC	3	0.960	0.865	1.066	6	1.201	0.979	1.472
	LMIC	-	-	-	-	3	1.933	1.591	2.348
**Age 50+**	Global	3	1.066	0.727	1.562	6	1.118	0.964	1.298
	HIC	3	1.066	0.727	1.562	3	1.193	0.983	1.447
	LMIC	-	-	-	-	3	1.042	1.032	1.053
**Allergic rhinitis**	Global	3	3.070	2.549	3.698	6	6.485	3.628	11.59
	HIC	3	3.070	2.549	3.698	3	3.885	3.038	4.968
	LMIC	-	-	-	-	3	11.962	3.105	46.083
**Atopy/Allergies**	Global	6	2.445	1.935	3.09	15	4.089	3.399	4.92
	HIC	3	2.147	1.756	2.625	11	4.294	3.516	5.245
	LMIC	3	3.210	2.550	4.040	4	3.567	2.171	5.859
**Bedroom sharing**	Global	3	0.975	0.709	1.340	-	-	-	-
	HIC	3	0.975	0.709	1.340	-	-	-	-
	LMIC	-	-	-	-	-	-	-	-
**Biomass**	Global	10	1.135	1.053	1.351	6	1.219	1.079	1.518
	HIC	6	1.010	0.892	1.145	3	1.065	0.831	1.366
	LMIC	4	1.506	1.089	2.522	3	1.295	1.053	1.760
**Birth weight <2.5 Kg**	Global	3	1.766	0.963	3.240	-	-	-	-
	HIC	3	1.766	0.963	3.240	-	-	-	-
	LMIC	-	-	-	-	-	-	-	-
**BMI <18.5 Kg/m²**	Global	-	-	-	-	3	1.270	0.781	2.068
	HIC	-	-	-	-	3	1.270	0.781	2.068
	LMIC	-	-	-	-	-	-	-	-
**BMI 25-29.9 Kg/m²**	Global	4	1.169	0.678	2.015	10	0.993	0.863	1.144
	HIC	4	1.169	0.678	2.015	10	0.993	0.863	1.144
	LMIC	-	-	-	-	-	-	-	-
**BMI 30+ Kg/m²**	Global	3	1.468	0.888	2.427	17	1.368	1.151	1.627
	HIC	3	1.468	0.888	2.427	13	1.438	1.207	1.712
	LMIC	-	-	-	-	4	1.184	0.856	1.638

Factor	WB region	Current wheezing				Ever asthma			
		Data points	Odds ratio	Lower CI	Upper CI	Data points	Odds ratio	Lower CI	Upper CI
**BMI >85th percentile**	Global	3	1.410	0.840	2.368	3	1.459	0.897	2.374
	HIC	-	-	-	-	3	1.459	0.897	2.374
	LMIC	3	1.410	0.840	2.368	-	-	-	-
**Carpets**	Global	3	0.925	0.501	1.708	6	1.029	0.862	1.229
	HIC	-	-	-	-	6	1.029	0.862	1.229
	LMIC	3	0.925	0.501	1.708	-	-	-	-
**Central heating**	Global	6	0.931	0.437	1.983	4	1.112	0.932	1.326
	HIC	3	1.290	1.162	1.432	4	1.112	0.932	1.326
	LMIC	3	0.590	0.313	1.111	-	-	-	-
**Cockroaches**	Global	6	1.216	0.549	2.69	-	-	-	-
	HIC	3	0.800	0.566	1.131	-	-	-	-
	LMIC	3	1.800	1.48	2.190	-	-	-	-
**Current smoker**	Global	7	2.164	1.647	2.844	14	1.205	1.075	1.351
	HIC	3	2.721	1.696	4.368	8	1.190	1.060	1.336
	LMIC	4	1.683	1.476	1.919	6	1.267	1.040	1.708
**Ever smoker**	Global	7	2.385	1.883	3.021	7	1.261	1.010	1.575
	HIC	4	2.084	1.781	2.438	4	1.102	1.065	1.258
	LMIC	3	4.483	2.985	6.733	3	1.578	1.242	2.005
**Ex smoker**	Global	3	1.604	1.222	2.105	8	1.242	1.069	1.443
	HIC	3	1.604	1.222	2.105	8	1.242	1.069	1.443
	LMIC	-	-	-	-	-	-	-	-
**Environmental tobacco smoke/ Second-hand smoke**	Global	10	1.039	0.833	1.295	14	1.234	1.063	1.431
	HIC	3	0.860	0.668	1.107	8	1.103	1.039	1.296
	LMIC	7	1.161	0.873	1.545	6	1.525	1.111	2.095
**Eczema**	Global	7	2.823	1.98	4.027	8	2.817	1.866	4.252
	HIC	4	2.395	1.533	3.741	4	2.606	2.216	3.065
	LMIC	3	3.761	1.709	8.281	4	2.816	1.073	7.390
**Exclusive breastfeeding**	Global	7	1.030	0.783	1.354	6	0.834	0.606	1.146
	HIC	3	1.266	0.974	1.644	3	1.098	0.796	1.514
	LMIC	4	0.795	0.593	1.065	3	0.546	0.334	0.893
**Family history of allergy**	Global	9	2.314	1.803	2.969	6	4.712	1.823	12.176
	HIC	6	2.272	1.706	3.025	3	3.010	2.246	4.033
	LMIC	3	2.460	1.672	3.620	3	5.702	0.729	44.633
**Family history of asthma**	Global	14	2.844	2.342	3.454	18	3.209	2.429	4.240
	HIC	5	2.306	1.936	2.746	12	2.876	2.450	3.376

	LMIC	9	3.310	2.443	4.484	6	4.529	2.435	8.422
**Family history atopy**	Global	7	1.458	1.153	1.842	10	1.981	1.430	2.746
	HIC	3	1.800	1.436	2.256	7	1.978	1.500	2.608
	LMIC	4	1.239	1.013	1.515	3	1.707	0.516	5.654
**Farming**	Global	3	0.846	0.636	1.125	-	-	-	-
	HIC	3	0.846	0.636	1.125	-	-	-	-
	LMIC	-	-	-	-	-	-	-	-
**Female**	Global	14	1.029	0.846	1.252	25	0.972	0.854	1.106
	HIC	8	1.086	0.838	1.407	22	0.967	0.840	1.113
	LMIC	6	0.941	0.692	1.279	3	1.007	0.757	1.338
**Male**	Global	15	1.176	0.984	1.406	20	1.159	0.981	1.369
	HIC	11	1.143	1.025	1.413	13	1.327	1.128	1.562
	LMIC	4	1.294	0.930	1.801	7	0.862	0.483	1.541
**Factor**	**WB region**	**Current wheezing**				**Ever asthma**			
		**Data points**	**Odds ratio**	**Lower CI**	**Upper CI**	**Data points**	**Odds ratio**	**Lower CI**	**Upper CI**
**Health insurance**	Global	-	-	-	-	3	1.057	0.974	1.148
	HIC	-	-	-	-	3	1.057	0.974	1.148
	LMIC	-	-	-	-	-	-	-	-
**High income**	Global	3	0.836	0.688	1.015	3	1.219	0.731	2.036
	HIC	3	0.836	0.688	1.015	3	1.219	0.731	2.036
	LMIC	-	-	-	-	-	-	-	-
**Low income**	Global	-	-	-	-	3	1.034	0.649	1.646
	HIC	-	-	-	-	3	1.034	0.649	1.646
	LMIC	-	-	-	-	-	-	-	-
**Hypertension**	Global	-	-	-	-	3	1.055	0.848	1.311
	HIC	-	-	-	-	3	1.055	0.848	1.311
	LMIC	-	-	-	-	-	-	-	-
**College or higher**	Global	6	1.068	0.918	1.244	8	0.883	0.703	1.111
	HIC	3	1.100	0.938	1.289	5	0.913	0.639	1.305
	LMIC	3	0.780	0.462	1.317	3	0.800	0.717	0.893
**Middle school or lower**	Global	3	1.066	0.727	1.562	8	1.021	0.849	1.228
	HIC	3	1.066	0.727	1.562	5	1.009	0.724	1.406
	LMIC	-	-	-	-	3	1.351	0.629	2.902
**Dampness**	Global	10	1.411	1.170	1.701	7	1.250	1.028	1.521
	HIC	7	1.391	1.171	1.651	4	1.301	1.075	1.574
	LMIC	3	1.537	0.862	2.742	3	1.071	0.523	2.191
**Mould**	Global	3	2.000	1.225	3.266	5	1.792	1.098	3.217
	HIC	3	2.000	1.225	3.266	5	1.792	1.098	3.217
	LMIC	-	-	-	-	-	-	-	-

**Indoor water leaking**	Global	6	1.352	1.051	1.740	6	1.470	0.918	2.354
	HIC	3	1.600	0.687	3.728	3	1.567	0.687	3.578
	LMIC	3	1.330	1.021	1.732	3	1.300	1.038	1.627
**Overcrowding**	Global	3	1.041	0.858	1.263	6	0.891	0.687	1.156
	HIC	-	-	-	-	3	0.962	0.690	1.343
	LMIC	3	1.041	0.858	1.263	3	0.736	0.514	1.056
**Parents high education**	Global	6	0.928	0.853	1.010	6	1.277	1.035	1.574
	HIC	3	0.946	0.851	1.051	3	1.210	0.964	1.520
	LMIC	3	0.888	0.760	1.037	3	1.780	1.083	2.927
**Parents' low education**	Global	6	1.130	0.792	1.612	8	0.938	0.748	1.177
	HIC	3	1.338	1.058	1.692	8	0.938	0.748	1.177
	LMIC	3	0.750	0.461	1.221	-	-	-	-
**Parents' low income**	Global	-	-	-	-	4	1.406	0.891	2.219
	HIC	-	-	-	-	4	1.406	0.891	2.219
	LMIC	-	-	-	-	-	-	-	-
**Parents' smoking**	Global	7	1.295	1.120	1.497	24	1.383	1.218	1.571
	HIC	4	1.296	1.066	1.574	18	1.207	1.085	1.343
	LMIC	3	1.300	1.026	1.646	6	1.862	1.431	2.424
**Parents' unemployed**	Global	3	0.980	0.789	1.218	3	1.804	1.119	2.907
	HIC	-	-	-	-	-	-	-	-
**Factor**	**WB region**	**Current wheezing**				**Ever asthma**			
		**Data points**	**Odds ratio**	**Lower CI**	**Upper CI**	**Data points**	**Odds ratio**	**Lower CI**	**Upper CI**
	LMIC	3	0.980	0.789	1.218	3	1.804	1.119	2.907
**Pets**	Global	19	1.059	0.964	1.163	21	1.212	0.979	1.502
	HIC	9	0.955	0.858	1.062	15	1.187	0.921	1.530
	LMIC	10	1.179	1.040	1.336	6	1.311	0.898	1.913
**Previous respiration infection**	Global	6	2.044	1.095	3.814	3	1.890	0.947	3.774
	HIC	3	1.537	0.930	2.538	3	1.890	0.947	3.774
	LMIC	3	3.600	2.830	4.580	-	-	-	-
**Rural**	Global	6	0.839	0.568	1.240	4	1.190	0.787	1.801
	HIC	3	0.924	0.582	1.467	4	1.190	0.787	1.801
	LMIC	3	0.630	0.438	0.906	-	-	-	-
**Urban**	Global	6	1.016	0.883	1.168	12	1.227	1.036	1.453
	HIC	3	1.003	0.822	1.223	7	1.093	0.896	1.335
	LMIC	3	1.057	0.821	1.360	5	1.456	1.151	1.842
**Siblings 2+**	Global	6	0.920	0.657	1.287	6	0.845	0.72	0.992
	HIC	3	0.826	0.671	1.016	3	0.822	0.677	0.999
	LMIC	3	1.300	0.365	4.624	3	0.950	0.669	1.349

**Traffic pollution**	Global	-	-	-	-	6	1.709	1.246	2.342
	HIC	-	-	-	-	3	1.914	0.582	6.297
	LMIC	-	-	-	-	3	1.611	1.426	1.819

CI – confidence interval, WB – World Bank.
There are 49 associated factors in total (45 Asthma ever and 43 Current wheeze). Blank spaces are those not reported or with less than 3 data points. HIC and LMIC datapoints add up to give the total.
*Meta-ORs included in the regional and national-level estimates.
ORs for binary variable associated factors are compared with those without the factor.

A total of 43 factors were explored for current wheezing and 45 were for ever asthma. Significant associated factors of both "current wheezing" and "ever asthma" globally included having history of allergic rhinitis, atopy or allergies for adults, and family history of asthma, atopy or allergies for children. This was also applicable for all categories of smoking (current, former, and ever) for adults, and having one or both parents smoking for children. In addition, having eczema was significantly associated with "current wheezing" and "ever asthma" in both adults and children globally. However, environmental tobacco smoke (second-hand tobacco smoke) was only statistically significant associated factor of "ever asthma" across all settings. Dampness, mould and indoor water leaking were major associated factors in LMICs. The effects of sex on "current wheezing" and "ever asthma" were still unclear. However, being a male was slightly associated with a higher risk in HICs. Obesity was also a major associated factor of "ever asthma" in HICs, while biomass exposures and traffic pollution were significant associated factors in LMICs

Additional notes on associated factors of asthma

The contributions of climate, weather and environmental factors to asthma in different settings are still subject to further research. For example, the high prevalence of asthma in some tropical settings may not explain similar or higher prevalence rates observed in nontropical settings in the EUR. This suggests an interplay of several contextual factors, including environmental, genetic, socioeconomic, and demographic factors and perhaps seasonal variations in the onset of respiratory infections, a known risk of chronic respiratory diseases.(4) Indeed, this complex interplay of risk factors plays important roles in the global pattern of asthma symptoms with important differences within and between countries.(5)

We could not directly account for indoor and outdoor air pollution, however, exposures to products of biomass combustion and traffic pollution are leading risk factors for asthma in LMICs, particularly in several cities in China and South-east Asia, where limited restrictions on traffic and industrial pollution have continued to have huge impacts on their large populations.(6, 7) For instance, in a recent global study, about 50% of emergency rooms visits attributable to poor air quality occurred in China, India, Pakistan and Bangladesh.(8) In emerging economies like Nigeria, Egypt and Ethiopia, the prevalent use of solid fuels like wood and charcoal for domestic purposes particularly in urban slums and rural areas, and harvesting and threshing in rural agriculture, are additional risks besides those from factory fumes, contributing to indoor air pollution and other unfavorable household conditions.(9, 10) Besides, there is a need to equally consider poor and vulnerable populations in HICs who are also exposed to adverse living conditions, air pollution, and damp environments.

Studies have shown that in genetically susceptible persons, being obese or overweight tends to increase the risk of asthma.(11, 12) In this study, obesity was only a significant risk factor for asthma in HICs, with our findings in LMICs rather inconclusive due to limited datasets reporting this. Other than a chronic inflammatory response, the mechanisms of the relationship between obesity and asthma across different contexts remain largely unknown.(12) There are speculations of a lack of differentiation between breathlessness in obese individuals and actual wheeze due to asthma.(13) Indeed, this association needs to be investigated further in large global studies, as many studies reporting the association appear not well powered or with low-quality evidence.(14) However, weight loss in obese asthma patients has been associated with significant improvements in asthma symptoms, exacerbations, exercise intolerance and response to some medications,(15) further suggesting a need for research to better understand the mechanism of this interaction to guide new therapies and specific management options. While we could not account specifically for age-sex differences in asthma risks across settings as most studies did not report this, hormonal factors have been indicated in the pattern of asthma observed in both sexes, with asthma common among boys before attaining puberty and girls having a higher risk thereafter.(7, 16, 17)

Meanwhile, the effects of exclusive breastfeeding in early life against asthma are still debated, we however found this to be modestly protective for asthma ever only in LMICs, albeit. The findings of a longitudinal study in Tasmania did show that exclusive breastfeeding may be protective in children before attaining 7 years but associated with increased risk in later life.(18) In a Canadian birth cohort, wheezing was reduced by 62% and 37% among infants with exclusive breastfeeding or partial breastfeeding at 6 months.(19)

References

1. United Nations Department of Economic and Social Affairs. 2018 Revision of World Urbanization Prospects. Available from: https://population.un.org/wup/. Accessed: 18 April 2021.
2. World Health Organization. The Global Health Observatory 2021. Available from: https://www.who.int/data/gho. Accessed: 18 April 2021.
3. Global Burden of Disease Collaborative Network. Global Burden of Disease Study 2019 (GBD 2019) Socio-Demographic Index (SDI) 1950–2019. Available from:

http://ghdx.healthdata.org/record/ihme-data/gbd-2019-socio-demographic-index-sdi-1950-2019. Accessed: 18 April 2021.
4. Baena-Cagnani CE. The global burden of asthma and allergic diseases: the challenge for the new century. Current Allergy & Asthma Reports. 2001;1(4):297-8.
5. Global Asthma Network. The Global Asthma Report 2018. Available from: http://www.globalasthmareport.org/resources/global_asthma_report_2018.pdf. Accessed: 18 April 2021.
6. India State-Level Disease Burden Initiative CRDC. The burden of chronic respiratory diseases and their heterogeneity across the states of India: the Global Burden of Disease Study 19902016. The Lancet Global Health. 2018;6(12):e1363-e74.
7. Li X, Song P, Zhu Y, Lei H, Chan KY, Campbell H, et al. The disease burden of childhood asthma in China: a systematic review and meta-analysis. Journal of global health. 2020;10(1):010801-
.
8. Anenberg SC, Henze DK, Tinney V, Kinney PL, Raich W, Fann N, et al. Estimates of the Global Burden of Ambient PM2.5, Ozone, and NO2 on Asthma Incidence and Emergency Room Visits. Environmental Health Perspectives. 2018;126(10):107004.
9. van Gemert F, van der Molen T, Jones R, Chavannes N. The impact of asthma and COPD in sub-Saharan Africa. Primary Care Respiratory Journal. 2011;20(3):240-8.
10. Adeloye D, Chan KY, Rudan I, Campbell H. An estimate of asthma prevalence in Africa: a systematic analysis. Croatian Medical Journal. 2013;54(6):519-31.
11. Peters U, Dixon AE, Forno E. Obesity and asthma. J Allergy Clin Immunol. 2018;141(4):1169-79.
12. Lv N, Xiao L, Camargo CA, Jr., Wilson SR, Buist AS, Strub P, et al. Abdominal and general adiposity and level of asthma control in adults with uncontrolled asthma. Annals of the American Thoracic Society. 2014;11(8):1218-24.
13. Global Initiative for Asthma. Global strategy for asthma management and prevention. https://ginasthmaorg/wp-content/uploads/2020/04/GINA-2020-full-report_-final-_wmspdf. Accessed: 20 April 2021.
14. Beuther DA, Sutherland ER. Overweight, obesity, and incident asthma: a meta-analysis of prospective epidemiologic studies. American Journal of Respiratory & Critical Care Medicine. 2007;175(7):661-6.
15. Juel CT, Ali Z, Nilas L, Ulrik CS. Asthma and obesity: does weight loss improve asthma control? a systematic review. Journal of asthma and allergy. 2012;5:21-6.
16. Dharmage SC, Perret JL, Custovic A. Epidemiology of Asthma in Children and Adults. Front Pediatr. 2019;7(246).
17. Guo X, Li Z, Ling W, Long J, Su C, Li J, et al. Epidemiology of childhood asthma in mainland China (1988-2014): A meta-analysis. Allergy & Asthma Proceedings. 2018;39(3):15-29. 18. Matheson MC, Erbas B, Balasuriya A, Jenkins MA, Wharton CL, Tang ML, et al. Breastfeeding and atopic disease: a cohort study from childhood to middle age. J Allergy Clin Immunol. 2007;120(5):1051-7.
19. Azad MB, Vehling L, Lu Z, Dai D, Subbarao P, Becker AB, et al. Breastfeeding, maternal asthma and wheezing in the first year of life: a longitudinal birth cohort study. The European respiratory journal. 2017;49(5).
